# Exploring the impact of aging on motor imagery abilities: a systematic review with meta-analysis

**DOI:** 10.3389/fpubh.2024.1405791

**Published:** 2025-01-23

**Authors:** José Fierro-Marrero, Mario González-Iglesias, Alberto Melis-Romeu, Javier Andrés López-Vidal, Alba Paris-Alemany, Roy La Touche

**Affiliations:** ^1^Departamento de Fisioterapia, Centro Superior de Estudios Universitarios La Salle, Universidad Autónoma de Madrid, Madrid, Spain; ^2^Motion in Brains Research Group, Centro Superior de Estudios Universitarios La Salle, Universidad Autónoma de Madrid, Madrid, Spain; ^3^PhD Program in Medicine and Surgery, Doctoral School, Universidad Autónoma de Madrid, Madrid, Spain; ^4^Department of Radiology, Rehabilitation and Physiotherapy, Faculty of Nursery, Physiotherapy and Podiatry, Complutense University of Madrid, Madrid, Spain; ^5^Instituto de Dolor Craneofacial y Neuromusculoesquelético (INDCRAN), Madrid, Spain

**Keywords:** motor imagery, movement imagery, mental representation, older adults, aging, geriatrics, systematic review, meta-analysis

## Abstract

**Objective:**

Explore motor imagery (MI) abilities in healthy older adults compared with healthy younger adults.

**Methods:**

A systematic review with meta-analysis.

**Results:**

Twenty-seven cross-sectional studies were included. Meta-analyses explored MI abilities between healthy older and younger adults for the ability to generate kinesthetic (60–70 years: *g* = −0.24, 95%CI = −1.61, 1.13; 70–80 years: *g* = −1.29, 95%CI = −2.75, 0.17), and visual modality (*g* = −0.08, 95%CI = −0.71, 0.86); vividness in kinesthetic (*g* = 0.14, 95%CI = −0.13, 0.41), IV (*g* = 0.11, 95%CI = −0.16, 0.38), and EV modalities (*g* = 0.05, 95%CI = −0.15, 0.24); mental chronometry in timed-up and go (seconds = 0.63, 95%CI = −0.02, 1.27), and linear walk (seconds = 0.75, 95%CI = −0.55, 2.06); and MI-execution time congruence (performance overestimation) in linear walk (*g* = −0.02, 95%CI = −0.73, 0.69). Mental chronometry in upper limb movements was analyzed visually in forest plot indicating tendencies of greater time in older adults. Hand recognition in hand laterality judgment task visual analysis revealed a poorer accuracy, greater response time and lower efficiency in older adults.

**Conclusion:**

Vividness of MI in kinesthetic and visual modalities appears to be preserved in older adults. Tendencies for greater time in mental chronometry were observed in older adults in TUG, linear walk and upper limb tasks. Implicit MI assessed with hand laterality showed older adults have lower accuracy, longer response times and lower efficiency. The ability to generate MI in kinesthetic and visual modalities presented imprecise results, and no clear conclusions could be drawn on MI-execution temporal congruence due to imprecision. Further research is needed to potentially clarify these findings.

**Systematic review registration:**

PROSPERO: CRD42023384916.

## Highlights

- The ability to generate motor imagery shows inconclusive results regarding how it varies with aging.
- Vividness during motor imagery remains preserved with healthy aging.
- Mental chronometry tends to be greater in older adults.
- Results from MI-execution temporal congruence are inconclusive.
- Implicit motor imagery, through hand recognition tasks, declines with aging, loosing accuracy, with greater response times, deriving into a lower efficiency.

## 1 Introduction

Aging is an intrinsic process of the human life cycle, in which physiological function declines, impacting cognitive (1), emotional (2–4), physical (5–7), and social spheres (8), affecting quality of life (9–11). Many sectors are influenced by aging, generating a high economic burden (12–14).

Physical functioning is a key component for healthy aging. It relies on the confluence of multiple integrated systems, including cognitive, emotional, sensory, musculoskeletal and cardiovascular systems. Among these, the proper functioning of motor-control-related components in the central and peripheral nervous system, alongside the integrity of musculoskeletal structures, is particularly critical. Several changes have been reported in literature to occur with aging. Notable changes include a reduction in the volume of several encephalic regions, such as the hippocampus, caudate nucleus, lateral prefrontal cortex, and the cerebellum, while other areas like the primary visual and entorhinal remain relatively unchanged (15). Additionally, white matter hyperintensities in the brain increase exponentially with age, doubling approximately every 10 years (16). In addition to these volumetric changes, aging is associated with reduced cerebral blood flow perfusion, particularly in cerebral white matter and certain cortical regions (17). On a peripheral level, aging affects secondary motor neurons through decreased depolarization frequencies, reduced persistent inward currents, and structural axonal changes (18). Despite age-related changes in the nervous system, older adults can adapt and learn new movements, showing neuroplasticity and improved cerebral efficiency. A recent review, pointed that skill training often reduces brain activation, reflecting greater neural efficiency in older adults. However, cortical hyperactivation remains common compared to younger adults (19).

MI is a mental process where the subject mentally simulates actions without its overt execution (20). MI can be subclassified into explicit and implicit MI (21). Explicit MI involves the mental performance of actions (22), whereas implicit MI entails the projection and manipulation of the body schema (23).

Explicit MI can be practiced using different strategies, which include 3 modalities: external visual (EV), internal visual (IV) and kinesthetic (KI). In the EV modality, subjects imagine their body movement from a third-person perspective, as though observing themselves from the outside. In contrast, the IV modality involves imagining body movements from a first person perspective, as if looking through its eyes. Lastly, in the KI modality, the focus is on the sensory experiences of the imagined movement, including tactile, proprioceptive and kinetic sensations.

A subject’s performance during explicit MI can be evaluated across its 3 modalities through various domains, which include: the ability to generate MI, vividness, mental chronometry, and MI-execution temporal congruence (24). The ability to generate MI refers to how challenging is for an individual to construct the MI process (25). This capacity is closely related to vividness, which refers to the realism of the MI experience (26). Depending on the modality employed (visual or kinesthetic) specific aspects can be assessed. For instance, in visual modalities, this includes the visual clarity of the imagined movement, while in KI modality, it refers to the intensity of the KI experience (26, 27). There is, however, some terminological ambiguity in the literature regarding the terms to describe the time taken to imagine an action and how closely this duration couples with the time required to physically execute it. In this review, we will use “mental chronometry” to refer to the time needed to imagine a movement and “MI-execution temporal congruence” to describe the degree of coupling between MI and execution durations.

It is important to note that explicit MI always involves an implicit evocation of the body schema (implicit MI). Therefore, the actual performance on explicit MI relies on the evocation of the body schema and other parameters related to the generation of its movement. To date, the closest method for assessing the quality of implicit MI relies on voluntarily evoking, manipulating and recognizing the body schema. This approach is typically evaluated through tasks that measure accuracy and response time in body recognition exercises, such as determining laterality (left or right side) of specific body parts (24) or whole-body images (28).

MI has been extensively studied as an intervention for motor learning, demonstrating its effectiveness in improving physical performance both as an isolated intervention (29), and in combination with physical practice. Several theories have proposed mechanisms to explain how MI facilitates motor learning without physical practice. These mechanisms include long-term potentiation (on the overlapping neural correlates with physical execution), the reorganization and refinement of motor plans, facilitation of movement encoding, and the anticipation to sensory stimuli (30).

The benefits of MI have been explored across various populations. In children and adolescents, it has been shown to enhance movement learning (31), and improving motor skills in healthy adults (32). Furthermore, its benefits extend to older adults (33), with evidence pointing that MI results in more pronounced strength gains in older than younger adults (34). Beyond healthy individuals, MI improves physical functioning in patients with neurological and musculoskeletal disorders (35, 36), and reduces pain perception (37).

Evidence from prior research has shown significant similarities in the central nervous system substrates involved in overt movement execution and MI in healthy subjects (38). In younger subjects, it has been observed that MI neural substrates vary across the employed modality. EV modality is primarily associated with spatial and temporal aspects of movement, activating brain areas related to visual perception, planning, and memory, particularly involving areas in the ventral stream (39). IV modality activates regions involved in movement planning and execution, such as parietal, frontal and occipital brain regions (39). This modality plays a relevant role in motor learning by integrating perception, action and memory. Among the visual modalities, the internal perspective appears to rely more heavily on motor system substrates (40). Lastly, KI modality activates subcortical brain regions, such as the bilateral caudate, along with cerebellum, primary and secondary somatosensory cortices, and temporal lobe areas (41). These regions are associated with sensory perception and motor control, and their activity closely mirrors the networks involved in overt movement, more so than either of the visual modalities (39, 42).

The shared neural regions suggest that age-related changes in motor control may not only lead to declines in physical function, as previously mentioned, but also may impair MI performance. The effects of aging could vary across different MI modalities, as even in younger individuals, these domains present slightly different neural correlates. This is a relevant question to address, as current literature points that older adults can be benefitted from MI interventions for improving motor performance. However, their basal abilities across the different MI domains may play a critical role for the effectiveness of MI interventions.

Various original studies have explored changes in MI abilities through aging, with previous literature reviews analyzing the tendency of these results. These reviews already detected a lower performance with aging in the ability to generate MI (43), mental chronometry, MI-execution temporal congruence, especially in complex tasks, and implicit MI (44). Conversely, outcomes such as MI vividness may be preserved (43, 44).

The objective with this systematic review relies on gathering the existing literature exploring differences in MI abilities between older (≥60 years) and younger (<60 years) healthy adults. Meta-analyses would be conducted to summarize the result of those studies.

## 2 Methodology

We followed Preferred Reporting Guidelines for Systematic Reviews and Meta-Analyses (45) during this systematic review. The protocol is listed as CRD42023384916 in the International Prospective Register of Systematic Reviews.

### 2.1 PICOS strategy

#### 2.1.1 Cases and controls

The case participants selected for study were healthy older adults (≥60 years), compared with healthy younger adults (18–59 years).

#### 2.1.2 Outcome measures

The outcome measures of interest included the following:

- Ability to generate MI: This variable explores the difficulty to construct MI. Procedures can vary, but they usually request the subject to overtly generate the movement, and imagine it afterwards, asking about the difficulty to generate that mental representation. Instruments such as the MIQ and others evaluate this phenomenon. Eligible outcome measures would include the ability to generate MI from KI, and visual (internal and external modalities grouped).
- Vividness of MI: The concept of vividness refers to the realism of the MI experience. It can also be explored in terms of visual clarity or KI intensity across in the respective modalities. Instruments such as the VMIQ assess this variable. Eligible outcome measures would include vividness of MI from EV, IV an KI modalities.
- Temporal features of MI: MI can be assessed with temporal features, such as the time or speed required to imagine an action. Eligible outcome measures would include the timed-up-and go (TUG), linear walk, and upper limb (UL) tasks.
- MI-execution temporal congruence: Temporal features of MI can be contrasted with the actual temporal features of the overtly executed task. This is usually conducted calculating the difference and/or ratio between MI and execution temporal features. This variable can be computed as “performance overestimation” when the subject imagines with a better performance than its actual execution $\left[\begin{array}{l} \mathrm{Executio}n_{\mathrm{time}}-Mi_{\mathrm{time}}\;\mathrm{or}\;\frac{\mathrm{Executio}n_{\mathrm{time}}}{MI_{\mathrm{time}}};\\ MI_{\mathrm{speed}}-\mathrm{Executio}n_{\mathrm{speed}}\;\mathrm{or}\;\frac{MI_{\mathrm{speed}}}{\mathrm{Executio}n_{\mathrm{speed}}}\; \end{array}\right]$ , or as “performance underestimation” when the subject imagines with a poorer performance than its actual execution $\left[\begin{array}{l} MI_{\mathrm{time}}-\mathrm{Executio}n_{\mathrm{time}}\;\mathrm{or}\;\frac{MI_{\mathrm{time}}}{\mathrm{Executio}n_{\mathrm{time}}};\\ \mathrm{Executio}n_{\mathrm{speed}}-MI_{\mathrm{speed}}\;\mathrm{or}\;\frac{\mathrm{Executio}n_{\mathrm{speed}}}{MI_{\mathrm{speed}}}\; \end{array}\right]$ . These two forms of estimating MI-execution temporal congruence would be eligible, exclusively for the TUG, linear walk, and UL tasks.
- Hand recognition: This outcome measure is an indicator of implicit MI through the recognition and manipulation of the body schema. The eligible tasks for this outcome would include the hand laterality judgment (HLJ) task. This task explores the ability of a subject to recognize left and right hands in different rotations and views (palm and/or back). Eligible measures would include accuracy, response time, and efficiency (the capacity to provide a correct response within its response time).

#### 2.1.3 Study design

Observational studies were eligible for inclusion.

### 2.2 Data sources and searches

Two independent reviewers employed the same search equations for MEDLINE (PubMed), EBSCO, Web of Science, SciELO, ScienceDirect, Scopus, and Google Scholar, in January 2023. Manual searches were performed until October 2023. Free terms, descriptors, and Boolean operators were used in the English searches, as well as Spanish terms (in Google Scholar). No language, population, study design, or time filter was used.

### 2.3 Selection process

The reviewers independently carried out screening and eligibility. This process was performed using the Rayyan AI tool (46), analyzing Title-Abstract and Full-Text. If there was insufficient information for inclusion, researchers would contact the corresponding authors for additional information.

Non-scientific articles, study protocols, and articles without full text were excluded. No language restrictions were applied. Additionally, in case of disagreement on selection, during the final eligibility phase a third reviewer was employed to reach a consensus.

### 2.4 Data extraction

Study information on authors, publication date, design, inclusion and exclusion criteria, population characteristics, groups, and sample size were extracted. Only groups meeting the age criteria (≥60 and < 60 years) were extracted. The following outcome measures were included: ability to generate KI or visual MI; vividness of MI in KI, IV, or EV modalities; mental chronometry or mental speed in TUG, linear walk, and UL movements; performance over/underestimation coefficients in TUG, linear walk, and UL movements; and accuracy, response time, and efficiency in HLJ task.

Instructions for testing temporal MI features, including modality, eye status, posture, and other varying difficulty constraints were recorded. Measurement tools were extracted, and the results were synthesized.

### 2.5 Methodological quality assessment

One reviewer assessed the methodological quality of studies with the Johanna Briggs Institute Critical Appraisal Checklist (JBI) for analytical cross-sectional studies [(47), p. 7]. The scale includes 8 items, with 4 response options (Yes; No; Unclear; and Not applicable). Authors provided a punctuation of 1 point for “Yes” responses, and 0 point for “No,” “Unclear” and “Not applicable” responses, accounting for a total score between 0 and 8 points. Greater punctuations would indicate a greater methodological quality.

### 2.6 Meta-analyses

The sample size, mean, and SD of outcomes were extracted for meta-analyses. Data were extracted from tables/text, and from graphics using the PDF X-Change Editor ruler.

Median and quartile data were converted to mean and SD using equations n°14 and 15 proposed by Wan et al. (48). Standard errors of the mean and confidence intervals were also transformed to SD following the Cochrane Handbook for Systematic Reviews of Interventions section 6.5.2.2 (49). Performance underestimation coefficients were transformed into overestimation coefficients.

Meta-analyses were conducted if (1) 3 or more studies explored the same outcome measure; (2) studies presented similar age groups; (3) the sample size, mean, and SD were available; and (4) they presented moderate or good methodological quality. In cases where only 1 or 2 studies explored a specific outcome, forest plots were included to provide a visual representation of effect sizes and trends. This exploratory approach was aimed to (1) offer preliminary insights into the direction and magnitude of effects between older and younger adults, even when data were sparse; (2) facilitate identification of potential patterns that could inform future studies or highlight gaps in the literature; and (3) ensure transparency in presenting all available evidence, minimizing the risk of selective reporting.

Random effect meta-analyses were conducted employing the Hedges’ model with a 95%CI. Pooled results were displayed as the raw mean difference (MD) if studies presented the same measurement instrument and unit. If not, data were treated with the Hedges’ *g* as standardized mean differences (50). The Hedges’ *g* is standardized mean difference (Cohen’s d) adjusted by the sample size, to prevent its overestimation. It will be interpreted following the criteria by Cohen (51): “very small” < 0.20; “small,” if 0.20–0.49; “medium” if 0.5–0.79, and “large” ≥ 0.8.

Heterogeneity was examined with Cochran’s *Q* test, the Inconsistency index (I^2^) and Tau squared (τ^2^). Cochran’s *Q* test presents limitations of underpower for meta-analyses with a low amount of studies or sample sizes; therefore, a *p-*value threshold of <0.1 would be used for considering heterogeneity across studies (52). Heterogeneity would be considered significant if either Cochran’s *Q* test *p-*value was <0.1, or *I*^2^ > 75%.

Funnel plots were employed for spotting outliers exceeding the 95%CI. Publication and selection bias was assessed with Egger’s Regression test (53), and Doi plot’s LFK index, with its threshold for detecting publication and selection bias if <−1 or > 1 (54). Publication and selection bias would be confirmed if any of the employed tests resulted positive.

Sensitivity analyses were explored with the Leave-One-Out Test (55) for meta-analyses with 4 or more studies. A significant influence would be confirmed if the extraction of any study would significantly modify the pooled result (generating a change over *p <* 0.05 or *p >* 0.05).

These procedures were conducted in R Studio software version 2023.06.0 + 421, employing R version 4.3.1 (56). MD, Hedges’ *g* calculations, random effect meta-analyses, and heterogeneity and sensitivity analyses were performed with the package “metafor” version 3.8.2 (57). Doi plots and LFK index were generated with the package “metasens” version 1.5–2 (58).

## 3 Results

### 3.1 Selection process

A total of 27 cross-sectional studies were included in the review (25, 59–84). The studies of Watanabe and Tani (82) and Kotegawa et al. (64) were included after the age range data were provided by the corresponding authors. Watanabe and Tani (82) additionally provided their data of mental chronometry.

**Table 1:** Quality assessment of analytical cross-sectional studies with Johanna-Briggs Institute Critical Appraisal Checklist.

Study	1. Inclusion criteria	2. Subjects and setting	3. Exposure validity and reliability	4. Exposure standardization	5. Confounding factors identified	6. Confounding factors controlled	7. Outcome measures validity and reliability	8. Statistical analysis	Total
Beauchet et al., 2018 (59)	Yes	Yes	Not applicable	Yes	Yes	Yes	No	Yes	6
Cacola et al., 2013 (60)	Yes	No	Not applicable	Unclear	Yes	Yes	No	Yes	4
Devlin and Wilson, 2010 (61)	Yes	No	Not applicable	No	No	No	No	Yes	2
Dommes et al., 2013 (62)	Yes	No	Not applicable	Yes	Unclear	Unclear	No	Yes	3
Kanokwan et al., 2019 (63)	Yes	No	Not applicable	Yes	Yes	Yes	No	Yes	5
Kotegawa et al., 2021 (64)	Yes	No	Not applicable	Yes	Yes	Yes	No	No	4
Liu et al., 2019 (65)	Yes	No	Not applicable	Yes	Yes	Yes	No	Yes	5
Malouin et al., 2010 (66)	Yes	Yes	Not applicable	No	Yes	Yes	No	Yes	5
Mitra et al., 2016 (67)	Yes	Yes	Not applicable	No	Yes	Yes	No	Yes	5
Mulder et al., 2007 (68)	No	No	Not applicable	No	No	No	No	Yes	1
Muto et al., 2022 (69)	Yes	Yes	Not applicable	Yes	Yes	Yes	No	Yes	6
Nagashima et al., 2021 (70)	Yes	Yes	Not applicable	Unclear	Yes	Yes	No	Yes	5
Naveteur et al., 2013 (71)	Yes	Yes	Not applicable	Yes	Yes	Yes	No	Yes	6
Raimo et al., 2021 (72)	Yes	Yes	Not applicable	Yes	Yes	Yes	No	Yes	6
Robin et al., 2021 (25)	Yes	No	Not applicable	No	No	No	No	Yes	4
Rulleau et al., 2018 (73)	Yes	No	Not applicable	No	No	No	No	Yes	2
Saimpont et al., 2009 (76)	Yes	Yes	Not applicable	Yes	Yes	Yes	No	Yes	6
Saimpont et al., 2012 (74)	Yes	Yes	Not applicable	Yes	Yes	Yes	No	Yes	6
Saimpont et al., 2015 (75)	Yes	No	Not applicable	Yes	Yes	Yes	No	Yes	5
Schott et al., 2012 (77)	Yes	Yes	Not applicable	Yes	Yes	Yes	No	Yes	6
Schott et al., 2013 (78)	Yes	Yes	Not applicable	Yes	Yes	Yes	No	Yes	6
Schott and Munzert, 2007 (79)	Yes	No	Not applicable	Yes	Yes	Yes	No	Yes	5
Skoura et al., 2005 (80)	Yes	No	Not applicable	Unclear	Yes	Yes	No	Yes	4
Wang et al., 2020 (81)	Unclear	No	Not applicable	No	Unclear	Unclear	No	Yes	1
Watanabe and Tani, 2022 (82)	Yes	No	Not applicable	Yes	Yes	Yes	No	Yes	5
Zhuang et al., 2020 (83)	Unclear	No	Not applicable	No	No	No	No	Yes	1
Zito et al., 2015 (84)	Yes	Yes	Not applicable	Yes	Yes	Yes	No	Yes	6

**Figure 1:**
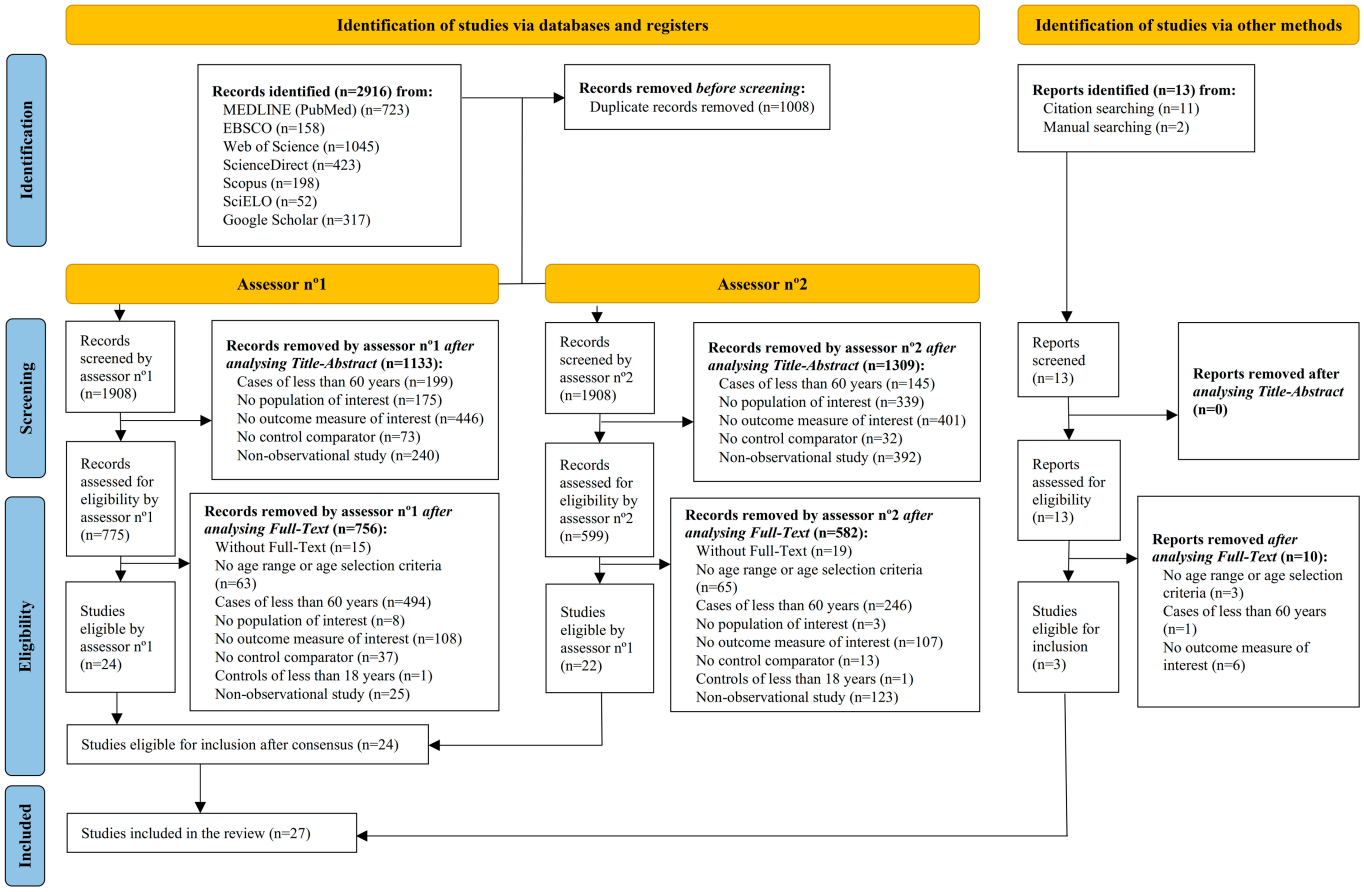
Flow chart synthesising the selection process of articles.

See Supplementary material for details of the search engines, databases, number of searches, and equations with their retrievals. Figure 1 represents the selection process via a flow-chart.

### 3.2 Methodological quality assessment

Studies presented an average methodological quality of 4.44 ± 1.69 (1–6 points). Nine studies presented 6 points (59, 69, 71, 72, 74, 76–78, 84), eight studies presented 5 points (63, 65–67, 70, 75, 79, 82), four studies reported 4 points (25, 60, 64, 80), one study 3 points (62), and two studies 2 points (61, 73) (see Table 1).

### 3.3 Data extraction

A total of 1,160 older adults (60–93 years) were compared to 1,345 younger adults (18–60 years). Among these participants, there were a total of 556 female older and 560 younger adults, with no sex information reported in 3 studies (25, 70, 84). See Table 2 for further details of demographic information.

**Table 2:** Summary information from the included studies.

Study	Selection	OA and YA groups	MI assessment domain		Task	Measurement tool	MI procedure		Results		
							Modality; Eyes; Posture	Other difficulty parameters	Summary	Overall	With varying difficulty
Beauchet et al., 2018 (59)	Inclusion: ≥20 years. Exclusion: Neurological, psychiatric, MMSE≤29, severe or acute medical conditions, and inability to walk 15 min unassisted.	OA group (*n* = 30): 75 ± 4.4 (70–87 years); F (*n* = 12). YA group (*n* = 30): 26.6 ± 7.4 (20–58 years); F (*n* = 17).	Explicit MI	Temporal features	iTUG	Mental chronometry (s)	Unspecified; As preferred; Same and different	Posture: sitting, standing and supine	No differences appeared in iTUG in any posture between groups.	OA ≈ YA	OA ≈ YA
			Synchrony	Perf. overest.	TUG	$\frac{\left(\mathrm{Ex}e_{\mathrm{time}}-MI_{\mathrm{time}}\right)\times 100}{\left(\mathrm{Ex}e_{\mathrm{time}}+MI_{\mathrm{time}}\right)/2}$	Unspecified; As preferred; Same and different	Posture: sitting, standing and supine	Performance overestimation did not differ between groups in any posture.	OA ≈ YA	OA ≈ YA
Caçola et al., 2013 (60)	Inclusion: Healthy participants of 18–93 years. Exclusion: Visual impairment, daily function impairment, neurological disorders, cognitive decline, low endurance and inability to maintain stance while seated.	OA group (*n* = 33): 74.52 ± 6.69 (65–93 years); F (*n* = 18). YA group (*n* = 33): 22.3 ± 2.72 (18–32 years); F (*n* = 16).	Explicit MI	Temporal features	Finger tapping task	Mental chronometry (s)	KI; Unspecified; Same	Number of body regions involved: Finger tapping with 3, 4 and 5 fingers	Older adults imagined slower than controls. MI time did not differ between groups across varying task difficulties.	OA > YA	OA ≈ YA
Devlin and Wilson 2010 (61)	Inclusion: Living independent and self–reported good health. Exclusion: Major neurological condition.	OA group (*n* = 18): 74.5 (65–87 years); F (*n* = 10). YA group (*n* = 19): 20.6 (19–24 years); F (*n* = 10).	Implicit MI	Hand recognition	HLJ task	Accuracy (error %)	–	Image rotation: Only back of the hand visible, 0°–330° in 30° increase	Older adults presented similar accuracy than controls. Only in some rotations, older adults presented less accuracy than controls.	OA ≈ YA	OA > YA
						Response time (ms)	–	Image rotation: Only back of the hand visible, 0°–330° in 30° increase	Older adults were slower than controls. Older adults were slower with increasing rotations	OA > YA	OA > YA
Dommes et al., 2013 (62)	Inclusion: Normal or corrected visual acuity, self–reported good health; living on their own and independently mobile. Exclusion: Confirmed cognitive decline through MMSE.	OA group 1 (*n* = 18): 76.9 ± 4.4 (70–84 years); F (*n* = 10). OA group 2 (*n* = 17): 62.8 ± 2.4 (60–67 years); F (*n* = 10). YA group (*n* = 16): 28.3 ± 4.3 (20–35 years); F (*n* = 8).	Synchrony	Perf. overest.	6 m linear walk	$\frac{MI_{\mathrm{speed}}\times 100\;}{\mathrm{Ex}e_{\mathrm{speed}}}$	Unspecified; Unspecified; Same	–	The oldest participants overestimated more their performance than younger old, and controls. No differences were observed between younger old and controls.	OA_1_ > YA OA_2_ ≈ YA	–
Kanokwan et al., 2019 (63)	Exclusion: inability to stand up from sitting, significant medical history, balance or motor function problems, mental disorders, MMSE<22, and MIQ–R < 20.	OA group (*n* = 19): 62.8 ± 2.4 (60–69 years); F (*n* = 10). YA group (*n* = 20): 28.3 ± 4.3 (20–29 years); F (*n* = 8).	Explicit MI	Capacity to generate MI – *KI modality*	MIQ–R	10–70 points	–	–	Authors did not analyze this outcome measure	–	–
				Capacity to generate MI – *Visual modalities (perspective unspecified)*	MIQ–R	10–70 points	–	–	Authors did not analyze this outcome measure	–	–
Kotegawa et al., 2021 (64) *Exp. nº1*	Inclusion: Healthy participants, independent walk Exclusion: ≤26 MMSE	OA group (*n* = 20): 74.5 ± 3.3 (70–82 years); F (*n* = 12). YA group (*n* = 15) from Kotegawa et al., 2020: 21.7 ± 4.4 (18–30 years); F (*n* = 7).	Explicit MI	Temporal features	5 m linear walk	Mental chronometry (s)	KI; Unspecified; Unspecified	Path width: 15, 25 and 50 cm	Authors did not analyze this outcome measure (but included in the meta–analysis with data from Kotegawa et al., 2020)	–	–
			Synchrony	Perf. overest.	5 m linear walk	$\frac{\left(\mathrm{Ex}e_{\mathrm{time}}-MI_{\mathrm{time}}\right)\times 100}{\mathrm{Ex}e_{\mathrm{time}}}$	KI; Unspecified; Unspecified	Path width: 15, 25 and 50 cm	No differences were observed between groups in any path–width condition.	OA ≈ YA	OA ≈ YA
Liu et al., 2019 (65) *Age range provided by authors*	Inclusion: Healthy through self–report. Exclusion: Attention <6, comprehension <6, and short–term memory <10, in Cognistat tool. Participant characteristics: No recent knee surgery or knee dysfunction impacting performance.	OA group (*n* = 20): 69.55 ± 7.0 (62–89 years); F (*n* = 9). YA group 1 (*n* = 43): 46.37 ± 8.6 (36–60 years); F (*n* = 29). YA group 2 (*n* = 31): 26.52 ± 5.8 (18–35 years); F (*n* = 22).	Explicit MI	Vividness – *KI modality*	VMIQ–2	12–60 points	–	–	No differences were observed between groups	OA ≈ YA	–
				Vividness – *IV modality*	VMIQ–2	12–60 points	–	–	No differences were observed between groups	OA ≈ YA	–
				Vividness – *EV modality*	VMIQ–2	12–60 points	–	–	No differences were observed between groups	OA ≈ YA	–
Malouin et al., 2010 (66)	Exclusion: pathological conditions, sensory impairments, immobilization in the last 6 months, medication affecting attention and alertness. Participant characteristics: Physically active and in good physical and mental health.	OA group (*n* = 19): 67.6 ± 4.6 (60–77 years); F (*n* = 10). YA group 1 (*n* = 15): 53.6 ± 5.4 (40–59 years); F (*n* = 9). YA group 2 (*n* = 46): 26 ± 5 (19–37 years); F (*n* = 23).	Explicit MI	Vividness – *KI modality*	KVIQ–10	10–50 points	–	–	No differences were observed between groups	OA ≈ YA	–
				Vividness – *IV modality*	KVIQ–10	10–50 points		–	No differences were observed between groups	OA ≈ YA	–
Mitra et al., 2016 (67)	Inclusion: Self–report normal or corrected to normal vision. Exclusion: Balance or neurological disorders.	OA group (*n* = 44): 70.9 ± 4.1 (65–80 years); F (*n* = 27). YA group (*n* = 41): 20.7 ± 2.4 (18–30 years); F (*n* = 20).	Explicit MI	Temporal features	Arm elevation	Mental chronometry (s)	Unspecified; Closed; Same	Direction: Forward or lateral Distance: 4 increasing distances	No differences were observed across groups or tasks	OA ≈ YA	OA ≈ YA
Mulder et al., 2007 (68)	Inclusion: Healthy participants. Exclusion criteria: NI.	OA group (*n* = 119): 74.2 ± 5.38 (67–93 years); F (*n* = 61). YA group (*n* = 143): 23.99 ± 3.01 (19–29 years); F (*n* = 71).	Explicit MI	Vividness – *EV modality*	VMIQ	24–120 points	–	–	No differences were observed between groups	OA ≈ YA	–
Muto et al., 2022 (69)	Inclusion: Normal or corrected vision, right–handed. Exclusion: history of neurological disorders, difficulty when moving limbs or walking, psychoactive drug treatment, or < 26 MMSE.	OA group (*n* = 71): 73.5 (60–87 years); F (*n* = 55). YA group (*n* = 28): 22.2 (20–27 years); F (*n* = 20).	Implicit MI	Hand recognition	HLJ task	Response time (ms)	–	Image rotation: 45°, 90°, 135°, 225°, 270°, 315º	Older adults presented greater response times than controls Older adults presented greater response times with increasing rotations	OA > YA	OA > YA
Nagashima et al., 2021 (70)	Inclusion: Absence of neurological diseases, mental disorders, UL disfunction, or visual impairment through a self–administered questionnaire.	OA group (*n* = 74): 73.2 ± 7.34 (60–88 years); F (NI). YA group (*n* = 68): 42.9 ± 8.23 (30–59 years); F (NI).	Implicit MI	Hand recognition	HLJ task	Accuracy (correct %)	–	–	No differences were observed between groups	OA ≈ YA	–
						Response time (s)	–	–	No differences were observed between groups	OA ≈ YA	–
						Efficiency (Response time / Accuracy)	–	–	No differences were observed between groups	OA ≈ YA	–
Naveteur et al., 2013 (71) *Exp. nº1*	Inclusion criteria: self–reported good health, >26 MMSE and visual acuity within normal limits or corrected. Exclusion criteria: other walking disability different from normal aging, diabetes, neurological and cardiac diseases.	OA group 1 (*n* = 12): 81.92 ± 4.85 (74–91 years); F (*n* = 12). OA group 2 (*n* = 12): 67.58 ± 3.37 (64–73 years); F (*n* = 12). YA group (*n* = 12): 26.17 ± 2.92 (22–31 years); F (*n* = 12).	Explicit MI	Temporal features	8 m linear walk	Mental chronometry (s)	Unspecified; Opened; Same	Obstacles: with and without curbs	No differences were observed between groups, nor with or without curbs	OA_1_ ≈ YA OA_2_ ≈ YA	OA_1_ ≈ YA OA_2_ ≈ YA
			Synchrony	Perf. underest.	8 m linear walk	$\left(MI_{\mathrm{time}}-\mathrm{Ex}e_{\mathrm{time}}\right)$	Unspecified; Opened; Same	Obstacles: with and without curbs	No differences were observed between groups, nor with or without curbs	OA_1_ ≈ YA OA_2_ ≈ YA	OA_1_ ≈ YA OA_2_ ≈ YA
Raimo et al., 2021 (72)	Inclusion: Normal scores in MMSE adjusted for education and age. Exclusion: Depression or anxiety diagnosed identified through DSM–5, cognitive impairment, deficit in abstract reasoning.	OA group (*n* = 37): 69.27 ± 8.53 (61–84 years); F (*n* = 25). YA group 1 (*n* = 50): 48.14 ± 4.16 (41–60 years); F (*n* = 30). YA group 2 (*n* = 49): 33.98 ± 4.26 (18–40 years); F (*n* = 25).	Implicit MI	Hand recognition	HLJ task	Accuracy (correct %)	–	–	No differences were observed between groups	OA ≈ YA	–
Robin et al., 2021 (25) *Age range provided by authors*	Inclusion: Healthy status. Exclusion: Under 18–year participants, musculoskeletal or neurological disorders.	OA group 1 (*n* = 30): 74.97 (71–82 years); F (NI). OA group 2 (*n* = 35): 64.77 (60–70 years); F (NI). YA group 1 (*n* = 32): 50.84 (45–59 years); F (NI). YA group 2 (*n* = 129): 31.35 (20–44 years); F (NI). YA group 3 (*n* = 45): 18.60 (18–19 years); F (NI).	Explicit MI	Capacity to generate MI –*KI modality*	MIQ–3sf	1–7 points	–	–	Both case groups presented a lower capacity compared only to control group nº2	OA_1_ < YA_2_ OA_2_ < YA_2_	–
				Capacity to generate MI – *IV modality*	MIQ–3sf	1–7 points	–	–	Both case groups presented a lower capacity compared only to control group nº2	OA_1_ < YA_2_ OA_2_ < YA_2_	–
				Capacity to generate MI – *EV modality*	MIQ–3sf	1–7 points	–	–	No differences were observed between groups	OA_1_ ≈ YA OA_2_ ≈ YA	–
Rulleau et al., 2018 (73) *Age ranges provided by authors*	Inclusion: NI. Exclusion: NI. Participant characteristics: Healthy and without current or history of nervous or muscular disorders	OA group (*n* = 32): 73.1 ± 4.6 (66–82 years); F (*n* = 19). YA group (*n* = 44): 19.4 ± 2 (18–25 years); F (*n* = 14).	Explicit MI	Vividness – *EV modality*	VMIQ	1–5 points	–	–	No differences were observed between groups	OA ≈ YA	–
Saimpont et al., 2009 (76)	Inclusion criteria: good health, right–handed, physical activity ≥2 days per week and at least one cognitive activity per day, visual span above the mean of their age range. Exclusion criteria: Abnormal or uncorrected vision, history of neurological or motor disorders, cognitive impairment (MMSE ≤26).	OA group (*n* = 19): 78.3 ± 4.5 (75–87 years); F (*n* = 12). YA group (*n* = 20): 23.9 ± 2.8 (20–30 years); F (*n* = 11).	Implicit MI	Hand recognition	HLJ task	Accuracy (correct %)	–	Rotations: 0°–270° in 90° increases	Older adults presented lower accuracy rate. Older adults presented lower accuracy with increasing rotations compared to controls	OA > YA	OA > YA
						Response time (ms)	–	Rotations: 0°–270° in 90° increases	Older adults presented greater response times. Older adults presented greater response times with increasing rotations compared to controls	OA > YA	OA > YA
Saimpont et al., 2012 (74)	Inclusion: ≥2/5 in KVIQ–10, ≥28 MMSE. Exclusion: Psychiatric, neurologic, or musculoskeletal disorders, balance or walking problems, use of a walking aid, chronic pain, medication affecting the level of vigilance, and uncorrected visual impairment.	OA groups (*n* = 26): 72.7 ± 5.5 (65–81 years); F (*n* = 18). YA group (*n* = 26): 23.2 ± 2.4 (19–28 years); F (*n* = 21).	Explicit MI	Vividness – *KI modality*	KVIQ–10	1–5 points	–	–	No differences were observed between groups	OA ≈ YA	–
				Vividness – *IV modality*	KVIQ–10	1–5 points	–	–	No differences were observed between groups	OA ≈ YA	–
				Temporal features	3 and 6 m linear walk	Mental chronometry (s)	KI and IV simultaneously; Closed; Same and different	Posture: sitting and standing Distance: 3 and 6 m	No differences were observed between groups neither with varying postures or walking distances	OA ≈ YA	OA ≈ YA
			Synchrony	Perf. underest.	3 and 6 m linear walk	$\frac{MI_{\mathrm{time}}}{\mathrm{Ex}e_{\mathrm{time}}}$	KI and IV simultaneously; Closed; Same and different	Posture: sitting and standing Distance: 3 and 6 m	No differences were observed between groups neither with varying postures or walking distances	OA ≈ YA	OA ≈ YA
Saimpont et al., 2015 (75)	Inclusion: Students, workers and active retirees, being in good health, good cognitive status (≥25 MMSE) and normal or corrected vision. Exclusion: Psychiatric, neurological or musculoskeletal disorders, or chronic pain.	OA group (*n* = 28): 72.4 ± 5.5 (65–81 years); F (*n* = 20). YA group (*n* = 30): 22.9 ± 2.7 (19–28 years); F (*n* = 25).	Explicit MI	Vividness – *KI modality*	KVIQ–10	1–5 points	–	–	No differences were observed between groups	OA ≈ YA	–
				Vividness – *IV modality*	KVIQ–10	1–5 points	–	–	No differences were observed between groups	OA ≈ YA	–
Schott et al., 2012 (77)	Inclusion: Regular physical activity and healthy cognitive status (≥26 MMSE). Exclusion criteria: Neurological disease, psychoactive or vasoactive medication consumption, or arthritis.	OA group 1 (*n* = 39): 83.08 ± 2.76 (≥80 years); F (*n* = 20). OA group 2 (*n* = 39): 74.56 ± 2.75 (70–79 years); F (*n* = 20). OA group 3 (*n* = 39): 63.74 ± 2.54 (60–69 years); F (*n* = 18). YA group (*n* = 40): 23.87 ± 2.5 (20–30 years); F (*n* = 20).	Explicit MI	Capacity to generate MI – *KI modality*	MIQ–R	1–7 points	–	–	Only ≥80, and of 70–79–year older adults, presented less capacity than controls. Older adults of 60–69 years did not differ from controls	OA_1_ < YA OA_2_ < YA OA_3_ ≈ YA	–
				Capacity to generate MI – *Visual modalities (perspective unspecified)*	MIQ–R	1–7 points	–	–	Only ≥80, and of 70–79–year older adults, presented less capacity than controls. Older adults of 60–69 years did not differ from controls	OA_1_ < YA OA_2_ < YA OA_3_ ≈ YA	–
				Temporal features	iTUG	Mental chronometry (s)	KI and IV simultaneously; Closed; Unspecified	–	No differences were observed between cases and controls	OA ≈ YA	–
					7, 10, 13, 16, 19, 22, 25 and 40 m linear walk	Mental chronometry (s)	KI and IV simultaneously; Closed; Unspecified	Distance: 7, 10, 13, 16, 19, 22, 25 and 40 m	No differences were observed between cases and controls. No differences between groups appeared with increasing path length	OA ≈ YA	OA ≈ YA
Schott et al., 2013 (78)	Inclusion: Cognitively unimpaired (≥26 MMSE), independency during daily living activities and low medication intake (≤4). Exclusion: Severe health condition, physical limitations, and use of walking aids.	OA group 1 (*n* = 43): 82.95 ± 2.33 (≥80 years); F (*n* = 21). OA group 2 (*n* = 44): 73.66 ± 2.57 (70–79 years); F (*n* = 23). OA group 3 (*n* = 45): 64.62 ± 3.28 (60–69 years); F (*n* = 23). YA group (*n* = 63): 23.25 ± 3.41 (20–30 years); F (*n* = 26).	Explicit MI	Capacity to generate MI – *KI modality*	MIQ–RS	1–7 points	–	–	No differences were observed between groups	OA_1_ ≈ YA OA_2_ ≈ YA OA_3_ ≈ YA	–
				Capacity to generate MI – *Visual modalities (perspective unspecified)*	MIQ–RS	1–7 points	–	–	No differences were observed between groups	OA_1_ ≈ YA OA_2_ ≈ YA OA_3_ ≈ YA	–
				Temporal features	iTUG	Mental chronometry (s)	Unspecified; Closed; Unspecified	–	No differences were observed between groups	OA_1_ ≈ YA OA_2_ ≈ YA OA_3_ ≈ YA	–
			Synchrony	Perf. overest.	TUG	$\frac{\mathrm{Ex}e_{\mathrm{time}}-MI_{\mathrm{time}}}{\mathrm{Ex}e_{\mathrm{time}}}$	Unspecified; Closed; Unspecified	–	Older adults greatly overestimated their performance compared to controls	OA > YA	–
Schott and Munzert 2007 (79)	Inclusion: Independent in BADL and participating in recreational programs, and cognitively healthy (≥26 MMSE). Exclusion: Substance abuse, brain surgery, CVD or cardiovascular disease, brain damage, psychiatric disorder or serious health problems. Population characteristics: MMSE 26–30.	OA group 1 (*n* = 12): 86.4 ± 3.2 (≥80 years); F (*n* = 12). OA group 2 (*n* = 10): 73.9 ± 3.1 (70–79 years); F (*n* = 10). YA group (*n* = 12): 21.5 ± 2.91 (19–32 years); F (*n* = 12).	Explicit MI	Vividness – *EV modality*	VMIQ	1–5 points	–	–	No differences were observed between groups	OA_1_ ≈ YA OA_2_ ≈ YA	–
				Temporal features	7, 10, 13, 16, 19, 22 and 25 m linear walk	Mental chronometry (s)	KI; Closed; Unspecified	Distance: 7, 10, 13, 16, 19, 22 and 25 m	Cases did not differ from controls in terms of mental chronometry across linear walk distances.	OA_1_ ≈ YA OA_2_ ≈ YA	OA_1_ ≈ YA OA_2_ ≈ YA
			Synchrony	Perf. underest.	7, 10, 13, 16, 19, 22 and 25 m linear walk	$MI_{\mathrm{time}}-\mathrm{Ex}e_{\mathrm{time}}$	KI; Closed; Unspecified	Distance: 7, 10, 13, 16, 19, 22 and 25 m	The oldest case group greatly overestimated its performance compared to controls. The younger older adults did not differ from cases. In both case groups performance overestimation increased with path distance, while this did not happen in controls	OA_1_ > YA OA_2_ ≈ YA	OA_1_ > YA OA_2_ > YA
Skoura et al., 2005 (80) *Exp. nº1*	Inclusion: Good health, normal or corrected vision. Exclusion: Nervous, muscular or cognitive disorder. Population characteristics: Cases were retired, performed regular physical activity, and daily cognitive activities. MMSE: 28.4 ± 0.9.	OA group 1 (*n* = 8): 73.4 ± 1.3 (72–75 years); F (*n* = 4). OA group 2 (*n* = 8) 66.2 ± 1.6 (64–68 years); F (*n* = 4). YA group (*n* = 8): 22.5 ± 1.4 (19–23 years); F (*n* = 4)	Explicit MI	Temporal features	8 m linear walk	Mental chronometry (s)	–	–	No differences were observed between groups	OA_1_ ≈ YA OA_2_ ≈ YA	–
					Sit–to–stand	Mental chronometry (s)	KI; Opened; Same	–	No differences were observed between groups	OA_1_ ≈ YA OA_2_ ≈ YA	–
					Arm–point task	Mental chronometry (s)	KI; Opened; Same	–	No differences were observed between groups	OA_1_ ≈ YA OA_2_ ≈ YA	–
*Exp. nº2*	Inclusion: Good health, normal or corrected vision. Exclusion: Nervous, muscular or cognitive disorder. Population characteristics: Cases were retired, performed regular physical activity, and daily cognitive activities. MMSE: 28.6 ± 0.7.	OA group 1 (*n* = 8): 73.2 ± 1.7 (71–75 years); F (*n* = 5). OA group 2 (*n* = 8): 64.8 ± 2 (62–67 years); F (*n* = 5). YA group (*n* = 8): 22 ± 1.9 (19–25 years); F (*n* = 4)	Explicit MI	Temporal features	Arm–point task	Mental chronometry (s)	KI; Opened; Same	Target size: 0.25, 1, 2.25, 4 cm^2^	No differences were observed between groups neither with increasing task difficulty	OA_1_ ≈ YA OA_2_ ≈ YA	OA_1_ ≈ YA OA_2_ ≈ YA
Wang et al., 2020 (81)	Inclusion: Normal or corrected vision. Exclusion: Movement difficulties related to any neurological or comorbid condition.	OA group 1 (*n* = 20): 74.25 ± 3.68 (70–81 years); F (*n* = 10). OA group 2 (*n* = 20): 64.10 ± 2.83 (60–69 years): F (*n* = 10). YA group 1 (*n* = 20): 54.75 ± 3.35 (50–59 years); F (*n* = 10). YA group 2 (*n* = 21): 44.14 ± 3.28 (40–49 years); F (*n* = 10). YA group 3 (*n* = 20): 35.3 ± 2.77 (30–39 years); F (*n* = 10). YA group 4 (*n* = 21): 25.14 ± 2.95 (20–29 years); F (*n* = 11).	Implicit MI	Hand recognition	HLJ task	Accuracy (correct %)	–	Rotation: 0–315° with 45° increases	No differences were observed between groups in overall. Increasing rotations generated a greater decrease in accuracy in cases compared to controls	OA_1_ ≈ YA OA_2_ ≈ YA	OA_1_ < YA OA_2_ < YA
						Response time (ms)	–	Image rotation: 0–315° with 45° increases	Only the oldest case group presented greater response times compared only to control group nº2. Older adults presented greater increases in response time with rotations, compared to controls	OA_1_ > YA OA_2_ ≈ YA	OA_1_ > YA OA_2_ > YA
Watanabe and Tani 2022 (82)	Inclusion: No experience using crutches or assistive devices. Exclusion: musculoskeletal, neurological or cognitive disorders (MMSE <24).	OA group (*n* = 39): 71.3 ± 2.9 (66–76 years); F (*n* = 13). YA group (*n* = 99): 20.2 ± 1.0 (19–21 years); F (*n* = 39).	Explicit MI	Temporal features	10 m linear walk	Mental chronometry (s)	Unspecified; Unspecified; Same	Crutches: with and without crutches	Authors did not analyze this outcome measure	–	–
			Synchrony	Perf. overest.	10 m linear walk	$\frac{\mathrm{Ex}e_{\mathrm{time}}-MI_{\mathrm{time}}}{\mathrm{Ex}e_{\mathrm{time}}}$	Unspecified; Unspecified; Same	Crutches: with and without crutches	Older adults presented less overestimation than younger adults with no crutches Older adults presented greater overestimations when using crutches compared to controls.	OA < YA	OA > YA
Zhuang et al., 2020 (83)	Inclusion: Normal or corrected normal vision. Exclusion: Motor impairments.	OA group (*n* = 27): 67.9 ± 4.92 (60–77 years); F (*n* = 14). YA group (*n* = 30): 20.9 ± 1.48 (18–24 years); F (*n* = 16).	Explicit MI	Temporal features	6.5, 13 and 19 m linear walk	Mental chronometry (s)	Unspecified; Opened; Unspecified	Distance: 6.5, 13 and 19 m	Authors did not specify the results of this analysis	–	–
			Synchrony	Perf. underest.	6.5, 13 and 19 m linear walk	$\frac{MI_{\mathrm{time}}}{\mathrm{Ex}e_{\mathrm{time}}}$	Unspecified; Opened; Unspecified	Distance: 6.5, 13 and 19 m	Older adults presented less underestimation (greater overestimation) of their crossing time compared to controls This underestimation was further reduced (overestimation was greatly increased) in older adults with longer distances	OA < YA	OA < YA
Zito et al., 2015 (84)	Inclusion: MoCA >26. Exclusion: Severely impaired motor abilities, inability to stand for about 1 h, or restricted visual field.	OA group (*n* = 18): 70.22 ± 4.11 (65–79 years); F (NI). YA group (*n* = 18): 25 ± 1.78 (23–28 years); F (NI)	Explicit MI	Temporal features	12 m linear walk	Mental speed (m/s)	Unspecified; Unspecified; Same	–	No differences were observed between groups	OA ≈ YA	–

BADL, basic activities of daily living; CVD, cerebrovascular disease; EV, external visual; Exe, executed; F, female; IV, internal visual; KI, kinesthetic; KVIQ-10, kinesthetic and visual imagery questionnaire; MI, motor imagery; MIQ-R, movement imagery questionnaire revised version; MIQ-3sf, movement imagery questionnaire 3 s French version; MoCA, Montreal cognitive assessment; MMSE, mini-mental state examination; NI, no information; OA, older adults; Overest, overestimation; Perf, performance; Underest, underestimation; VMIQ, vividness of movement imagery questionnaire; VMIQ-2, vividness of movement imagery questionnaire 2^nd^ version; YA; younger adults.

All studies included participants that were self-reported or considered healthy by the researcher, additionally excluding participants with physical or musculoskeletal impairments in 23 studies (25, 59, 60, 62–67, 69–71, 73–78, 80–84), neurological or mental conditions in 18 studies (59–61, 63, 67, 69–77, 79–82), and diminished cognitive functions in 17 studies (59, 62–65, 69, 71, 72, 74–80, 82, 84). See Table 2 for further details of studies’ eligibility criteria.

#### 3.3.1 Explicit MI

Nineteen studies explored explicit MI domains (25, 59, 60, 63–68, 71, 73–75, 77–80, 82, 83).

##### 3.3.1.1 Ability to generate MI – kinesthetic modality

Four studies explored the ability to generate KI MI (25, 63, 77, 78). The instruments included MIQ-3sf (25), MIQ-R (63, 77), and MIQ-RS (78).

##### 3.3.1.2 Ability to generate MI – visual modalities

Three studies explored this outcome measure, not specifying the perspective (first or third) (63, 77, 78), 1 study from IV (25), and 1 study from EV modalities (25). The instruments included MIQ-3sf (25), MIQ-R (63, 77), and MIQ-RS (78).

##### 3.3.1.3 Vividness – kinesthetic modality

Four studies explored vividness during MI from a KI modality (65, 66, 74, 75). Assessment tools included the Vividness of Movement Imagery Questionnaire revised version (VMIQ-2) (65), and the Kinesthetic and Visual Imagery Questionnaire (KVIQ-10) (66, 71, 74, 75).

##### 3.3.1.4 Vividness – internal visual modality

Four studies explored vividness during MI from a IV modality (65, 66, 74, 75). Assessment tools included the VMIQ-2 (65), and the KVIQ-10 (66, 71, 74, 75).

##### 3.3.1.5 Vividness – external visual modality

Four studies explored vividness during MI from EV modality (65, 68, 73, 79). Assessment tools included the VMIQ original version (68, 73, 79), and the VMIQ-2 (65).

##### 3.3.1.6 Temporal features MI

Fourteen studies explored temporal features (59, 60, 63, 64, 67, 71, 74, 77–80, 82–84), in which they explored mental chronometry (59, 60, 63, 64, 67, 71, 74, 77–80, 82–84), and mental speed (84).

Studies explored these features for imagined TUG (59, 77, 78), imagined linear walk (64, 71, 74, 77, 79, 80, 82–84), arm elevation movements (67, 80), and finger tapping task (60).

#### 3.3.2 MI-execution temporal congruence

MI-execution temporal congruence, through difference or ratios between MI and execution temporal features were assessed in 9 studies, with 5 computing “performance overestimation” measures (59, 62, 64, 78, 82), and 4 “performance underestimation” measures (71, 74, 79, 83). Two studies explored these variables for imagined TUG (59, 78), and 7 explored linear walk (62, 64, 71, 74, 79, 82, 83). No studies explored this variable for UL movements.

#### 3.3.3 Implicit MI

Eight studies explored implicit MI through the HLJ task (61, 69, 70, 72, 76–78, 81).

##### 3.3.3.1 Hand recognition – accuracy

Five studies explored hand recognition ability through the hand laterality judgment (HLJ) in terms of accuracy (61, 70, 72, 76, 81). These studies explored this outcome grouping the results across different hand rotations (70, 72), and analyzing specifically the outcome at 0° (61, 76, 81), 30° (61), 45° (81), 60° (61), 90° (61, 76, 81), 120° (61), 135° (81), 150° (61), and 180° rotation (61, 76, 81).

##### 3.3.3.2 Hand recognition – response time

Five studies explored response time in the HLJ task (61, 69, 70, 76, 81). One study explored response time grouping the results across different hand rotations (70), at 0° (61, 76, 81), 30° (61), 45° (69, 81), 60° (61), 90° (61, 69, 76, 81), 120° (61), 135° (69, 81), 150° (61), and 180° (61, 76, 81).

##### 3.3.3.3 Hand recognition – efficiency

One study explored the HLJ task in terms of efficiency grouping different angular rotations for palm, and back views for medial and lateral rotations (70). This was explored with the inverse efficiency score, a ratio between response time and the rate of correct responses.

### 3.4 Meta-analyses

Based on the criteria for conducting meta-analyses, the authors were only able to conduct meta-analyses for (1) ability to generate MI from KI modality conducting 2 meta-analyses based on age groups; (2) ability to generate MI from visual modality; (3) vividness of MI in KI modality; (4) vividness of MI in IV modality; (5) vividness of MI in EV modality; (6) Temporal features of MI (in terms of mental chronometry) for TUG, and linear walk tasks; and (7) MI-execution temporal congruence (in terms of performance overestimation) for linear walk tasks. The following outcome measures did not fulfill the criteria for conducting meta-analyses (number of available studies): temporal features of MI (in terms of mental chronometry) for UL tasks, hand recognition accuracy, hand recognition response time and hand recognition efficiency. However, forest plots were presented for observing difference tendencies between groups. See Table 3 for the detailed process to select studies in meta-analyses.

**Table 3:** Data availability, extraction and processing for meta-analyses between healthy older and younger adults.

Outcome measure	Eligible studies (k)	Text/Table or Plot (k)	Included in the meta-analysis	
			Extractable (k)	Raw extraction as Mean and SD (k)
Capacity to generate MI – kinesthetic modality – 60-70 years	4 studies: Kanokwan et al., 2019; Robin et al., 2021; Schott, 2013, 2012	Text/Table (4): Kanokwan et al., 2019; Robin et al., 2021; Schott, 2013, 2012	Yes (4): Kanokwan et al., 2019; Robin et al., 2021; Schott, 2013, 2012	Yes (4): Kanokwan et al., 2019; Robin et al., 2021; Schott, 2013, 2012
Capacity to generate MI – kinesthetic modality – 70-82 years	3 studies: Robin et al., 2021; Schott, 2013, 2012	Text/Table (3): Robin et al., 2021; Schott, 2013, 2012	Yes (3): Robin et al., 2021; Schott, 2013, 2012	Yes (3): Robin et al., 2021; Schott, 2013, 2012
Capacity to generate MI – visual modalities	3 studies: Kanokwan et al., 2019; Schott, 2013, 2012	Text/Table (3): Kanokwan et al., 2019; Schott, 2013, 2012	Yes (3): Kanokwan et al., 2019; Schott, 2013, 2012	Yes (3): Kanokwan et al., 2019; Schott, 2013, 2012
Vividness – kinesthetic modality	4 studies: Liu et al., 2019; Malouin et al., 2010; Saimpont et al., 2015, 2012	Text/Table (3): Liu et al., 2019; Saimpont et al., 2015, 2012	Yes (3): Liu et al., 2019; Saimpont et al., 2015, 2012	Yes (1): Liu et al., 2019
				No (2): Saimpont et al., 2015^*^, 2012^*^
		Graphics (1): Malouin et al., 2010	Yes (1): Malouin et al., 2010	No (1): Malouin et al., 2010 ^⁑^
Vividness – internal visual modality	4 studies: Liu et al., 2019; Malouin et al., 2010; Saimpont et al., 2015, 2012	Text/Table (3): Liu et al., 2019; Saimpont et al., 2015, 2012	Yes (3): Liu et al., 2019; Saimpont et al., 2015, 2012	Yes (1): Liu et al., 2019
				No (2): Saimpont et al., 2015^*^, 2012^*^
		Graphics (1): Malouin et al., 2010	Yes (1): Malouin et al., 2010	No (1): Malouin et al., 2010 ^⁑^
Vividness – external visual modality	4 studies: Liu et al., 2019; Mulder et al., 2007; Rulleau et al., 2018; Schott and Munzert, 2007	Text/Table (4): Liu et al., 2019; Mulder et al., 2007; Rulleau et al., 2018; Schott and Munzert, 2007	Yes (4): Liu et al., 2019; Mulder et al., 2007; Rulleau et al., 2018; Schott and Munzert, 2007	Yes (3): Liu et al., 2019; Rulleau et al., 2018; Schott and Munzert, 2007
				No (1): Mulder et al., 2007 ^⁂^
Temporal features of MI (mental chrometry) – TUG	3 studies: Beauchet et al., 2018; Schott, 2013, 2012.	Text/Table (3): Beauchet et al., 2018; Schott, 2013, 2012.	Yes (3): Beauchet et al., 2018; Schott, 2013, 2012.	Yes (3): Beauchet et al., 2018; Schott, 2013, 2012.
Temporal features of MI (mental chrometry) – Linear Walk (5–10 m)	8 studies: Kotegawa et al., 2021; Naveteur et al., 2013; Saimpont et al., 2012; Schott et al., 2012; Schott and Munzert, 2007; Skoura et al., 2005; Watanabe and Tani, 2022; Zhuang et al., 2020	Text/Table (1): Schott et al., 2012	Yes (1): Schott et al., 2012	Yes (1): Schott et al., 2012
		Graphics (5): Kotegawa et al., 2021; Saimpont et al., 2012; Schott and Munzert, 2007; Skoura et al., 2005; Zhuang et al., 2020.	Yes (4): Kotegawa et al., 2021; Saimpont et al., 2012; Skoura et al., 2005; Zhuang et al., 2020	Yes (2): Saimpont et al., 2012; Skoura et al., 2005
				No (2): Kotegawa et al., 2021 ^‡^; Zhuang et al., 2020 ^‡^
			No (1): Schott and Munzert, 2007	–
		Provided by authors (1): Watanabe and Tani, 2022	Yes (1): Watanabe and Tani, 2022	Yes (1): Watanabe and Tani, 2022
		Not available (1): Naveteur et al., 2013	No (1): Naveteur et al., 2013	–
Temporal features of MI (mental chrometry) – UL tasks	2 studies: Mitra et al., 2016; Skoura et al., 2005	Graphics (2): Mitra et al., 2016; Skoura et al., 2005	Yes (2): Mitra et al., 2016; Skoura et al., 2005	Yes (1): Skoura et al., 2005
				No (1): Mitra et al., 2016 ^‡^^,^ ^Ө^
MI-execution temporal congruence (performance overestimation) – Linear Walk (5–10 m)	3 studies of performance overestimation: Dommes et al., 2013; Kotegawa et al., 2021; Watanabe and Tani, 2022	Text/Table (2): Dommes et al., 2013; Watanabe and Tani, 2022	Yes (2): Dommes et al., 2013; Watanabe and Tani, 2022	Yes (2): Dommes et al., 2013; Watanabe and Tani, 2022
		Graphics (1): Kotegawa et al., 2021 (and Kotegawa et al., 2019 for comparison)	Yes (1): Kotegawa et al., 2021 (and Kotegawa et al., 2019 for comparison)	No (1): Kotegawa et al., 2021 ^‡^ (and Kotegawa et al., 2019 for comparison ^‡^)
			Yes (1): Kotegawa et al., 2019	No (1): Kotegawa et al., 2019 ^‡^
	4 studies of performance underestimation: Naveteur et al., 2013; Saimpont et al., 2012; Schott and Munzert, 2007; Zhuang et al., 2020	Text/Table (2): Saimpont et al., 2012; Zhuang et al., 2020	Yes (2): Saimpont et al., 2012; Zhuang et al., 2020	No (2): Saimpont et al., 2012 ^§^; Zhuang et al., 2020 ^‡^^,^ ^§^
		Graphics (1): Schott and Munzert, 2007	No (1): Schott and Munzert, 2007	–
		Not available (1): Naveteur et al., 2013	–	–
Hand recognition – Accuracy (grouped rotations)	2 studies analyzing correct responses: Nagashima et al., 2021; Raimo et al., 2021	Text/Table (1): Nagashima et al., 2021	Yes (1): Nagashima et al., 2021	Yes (1): Nagashima et al., 2021
		Graphics (1): Raimo et al., 2021	Yes (1): Raimo et al., 2021	No (1): Raimo et al., 2021ꭝ
Hand recognition – Accuracy (0° rotation)	2 studies analyzing correct responses: Saimpont et al., 2009; Wang et al., 2020	Graphics (2): Saimpont et al., 2009; Wang et al., 2020	Yes (1): Wang et al., 2020	No (1): Wang et al., 2020 ^‡^
			No (1): Saimpont et al., 2009	–
	1 study analyzing errors: Devlin and Wilson 2010	Graphics (1): Devlin and Wilson 2010	Yes (1): Devlin and Wilson 2010	No (1): Devlin and Wilson 2010 ^†^^,^ ^‡^
Hand recognition – Accuracy (90° rotation)	2 studies analyzing correct responses: Saimpont et al., 2009; Wang et al., 2020	Graphics (2): Saimpont et al., 2009; Wang et al., 2020	Yes (1): Wang et al., 2020	No (1): Wang et al., 2020 ^‡^
			No (1): Saimpont et al., 2009	–
	1 study analyzing errors: Devlin and Wilson 2010	Graphics (1): Devlin and Wilson 2010	Yes (1): Devlin and Wilson 2010	No (1): Devlin and Wilson 2010 ^†^^,^ ^‡^
Hand recognition accuracy in HLJ (180° rotation)	2 studies analyzing correct responses: Saimpont et al., 2009; Wang et al., 2020	Graphics (2): Saimpont et al., 2009; Wang et al., 2020	Yes (1): Wang et al., 2020	No (1): Wang et al., 2020 ^‡^
			No (1): Saimpont et al., 2009	–
	1 study analyzing errors: Devlin and Wilson 2010	Graphics (1): Devlin and Wilson 2010	Yes (1): Devlin and Wilson 2010	No (1): Devlin and Wilson 2010 ^†^^,^ ^‡^
Hand recognition – Response time (grouped rotations)	1 study: Nagashima et al., 2021	Text/Table (1): Nagashima et al., 2021	Yes (1): Nagashima et al., 2021	Yes (1): Nagashima et al., 2021
Hand recognition – Response time (0° rotation)	3 studies: Devlin and Wilson 2010, Saimpont et al., 2009, Wang et al., 2020	Graphics (3): Devlin and Wilson 2010, Saimpont et al., 2009, Wang et al., 2020	Yes (1): Devlin and Wilson 2010	No (1): Devlin and Wilson 2010 ^‡^
			No (2): Saimpont et al., 2009, Wang et al., 2020	–
Hand recognition – Response time (30° rotation)	1 study: Devlin and Wilson 2010	Graphics (1): Devlin and Wilson 2010	Yes (1): Devlin and Wilson 2010	No (1): Devlin and Wilson 2010 ^‡^
Hand recognition – Response time (45° rotation)	2 studies: Muto et al., 2022, Wang et al., 2020	Text and Graphics (1): Muto et al., 2022	No (1): Muto et al., 2022	–
		Graphics (1): Wang et al., 2020	No (1): Wang et al., 2020	–
Hand recognition – Response time (60° rotation)	1 study: Devlin and Wilson 2010	Graphics (1): Devlin and Wilson 2010	Yes (1): Devlin and Wilson 2010	No (1): Devlin and Wilson 2010 ^‡^
Hand recognition – Response time (90° rotation)	4 studies: Devlin and Wilson 2010, Muto et al., 2022, Saimpont et al., 2009, Wang et al., 2020	Text and Graphics (1): Muto et al., 2022	No (1): Muto et al., 2022	–
		Graphics (3): Devlin and Wilson 2010, Saimpont et al., 2009, Wang et al., 2020	Yes (1): Devlin and Wilson 2010	No (1): Devlin and Wilson 2010 ^‡^
			No (2): Saimpont et al., 2009, Wang et al., 2020	–
Hand recognition – Response time (120° rotation)	1 study: Devlin and Wilson 2010	Graphics (1): Devlin and Wilson 2010	Yes (1): Devlin and Wilson 2010	No (1): Devlin and Wilson 2010 ^‡^
Hand recognition – Response time (135° rotation)	2 studies: Muto et al., 2022, Wang et al., 2020	Text and Graphics (1): Muto et al., 2022	No (1): Muto et al., 2022	–
		Graphics (1): Wang et al., 2020	No (1): Wang et al., 2020	–
Hand recognition – Response time (150° rotation)	1 study: Devlin and Wilson 2010	Graphics (1): Devlin and Wilson 2010	Yes (1): Devlin and Wilson 2010	No (1): Devlin and Wilson 2010 ^‡^
Hand recognition – Response time (180° rotation)	3 studies: Devlin and Wilson 2010, Saimpont et al., 2009, Wang et al., 2020	Graphics (3): Devlin and Wilson 2010, Saimpont et al., 2009, Wang et al., 2020	Yes (1): Devlin and Wilson 2010	No (1): Devlin and Wilson 2010 ^‡^
			No (2): Saimpont et al., 2009, Wang et al., 2020	–
Hand recognition – Efficiency (back view medial rotations grouped)	1 study: Nagashima 2021	Graphics (1): Nagashima 2021	Yes (1): Nagashima 2021	No (1): Nagashima 2021 ^‡^
Hand recognition – Efficiency (back view lateral rotations grouped)	1 study: Nagashima 2021	Graphics (1): Nagashima 2021	Yes (1): Nagashima 2021	No (1): Nagashima 2021 ^‡^
Hand recognition – Efficiency (palm view medial rotations grouped)	1 study: Nagashima 2021	Graphics (1): Nagashima 2021	Yes (1): Nagashima 2021	No (1): Nagashima 2021 ^‡^
Hand recognition – Efficiency (palm view lateral rotations grouped)	1 study: Nagashima 2021	Graphics (1): Nagashima 2021	Yes (1): Nagashima 2021	No (1): Nagashima 2021 ^‡^

* In the KVIQ-10 (1–5 points) tool, 1-point is the lowest and 5-points the highest achievable vividness score. Its mean score was transformed into a scale where 1-point would be the highest and 5-points the lowest achievable vividness score (inverted KVIQ-10 scale): $\left[\mathrm{Mean Inverted KVIQ}=5-\mathrm{Mean KVIQ}\right]$ . SD would not be transformed.

⁑ In the KVIQ-10 (10–50 points) tool, 10-points is the lowest and 50-points the highest achievable vividness score. Its mean score was transformed into a scale where 1-point would be the highest and 5-points the lowest achievable vividness score (inverted KVIQ-10 scale). $\left[\mathrm{Mean Inverted KVIQ}=50-\mathrm{Mean KVIQ}\right]$ . SD would not be transformed.

⁂ 95%CI to SD: ￼

‡ SE to SD: ￼

Ө Milliseconds to Seconds for both mean ￼; and SD, ￼

§ Performance underestimation (Perf. Underest.) $\left[\frac{MI\;\mathrm{time}}{\mathrm{Execution Time}}\right]$ , into performance overestimation (Perf. Overest.) $\left[\frac{\mathrm{Execution time}}{MI\;\mathrm{Time}}\right]$ for mean $\left[\mathrm{Mean Perf}.\mathrm{Overerst}\approx \frac{1}{\mathrm{Mean Perf}.\;\mathrm{Underest}\text{.}}\right]$ and SD $\left[SD\;\mathrm{Perf}.\mathrm{Overerst}\approx \frac{SD\;\mathrm{Perf}.\;\mathrm{Underest}}{{\left(\mathrm{Mean Perf}.\;\mathrm{Underest}\right)}^{2}}\right]$ . Simulations were conducted with the function “rnorm,” from the package “compositions” version 2.0.8, in R Software version 4.3.1, with the following syntaxis.

# Install compositions package.

install.packages (“compositions”).

# Activate compositions package.

require (compositions).

# Generate simulated normal distribution data with n, mean, and SD for MI and Execution time.

MI_time = rnorm (*n* = 1,000, mean = 300, sd = 10).

Execution_time = rnorm (*n* = 1,000, mean = 400, sd = 10).

# Generate ratios between MI and Execution time as underestimation and overestimation.

Underestimation = MI_time / Execution_time.

Overestimation = Execution_time / MI_time.

# Report the Mean underestimation and Mean overestimation.

mean (Underestimation).

mean (Overestimation).

# Estimate mean overestimation from mean underestimation data.

1/mean (Underestimation).

# Report the SD of underestimation and SD of overestimation.

sd (Underestimation).

sd (Overestimation).

# Estimate overestimation SD from underestimation SD.

sd (Underestimation)/mean (Underestimation)^2.

ꭝ Median, Q3 and Q1 to mean $\left[\frac{Q_{3}+Mdn+Q1}{3}\right]$ , and SD $\left[\frac{Q_{3}-Q1}{1.35}\right]$ .

† Mean error rate (%) to mean accuracy rate (%): $\left[\mathrm{Mean accuracy rate}=100-\mathrm{Mean error rate}\right]$ .

#### 3.4.1 Ability to generate MI – kinesthetic modality – older adults aged 60–70 years

Four studies explored this outcome measure and were included in the meta-analysis. Healthy older adults aged 60–70 years were compared with healthy younger adults aged 18–30 years (25, 63, 77, 78). Studies presented a methodological quality of 4–6 points.

**Figure 2:**
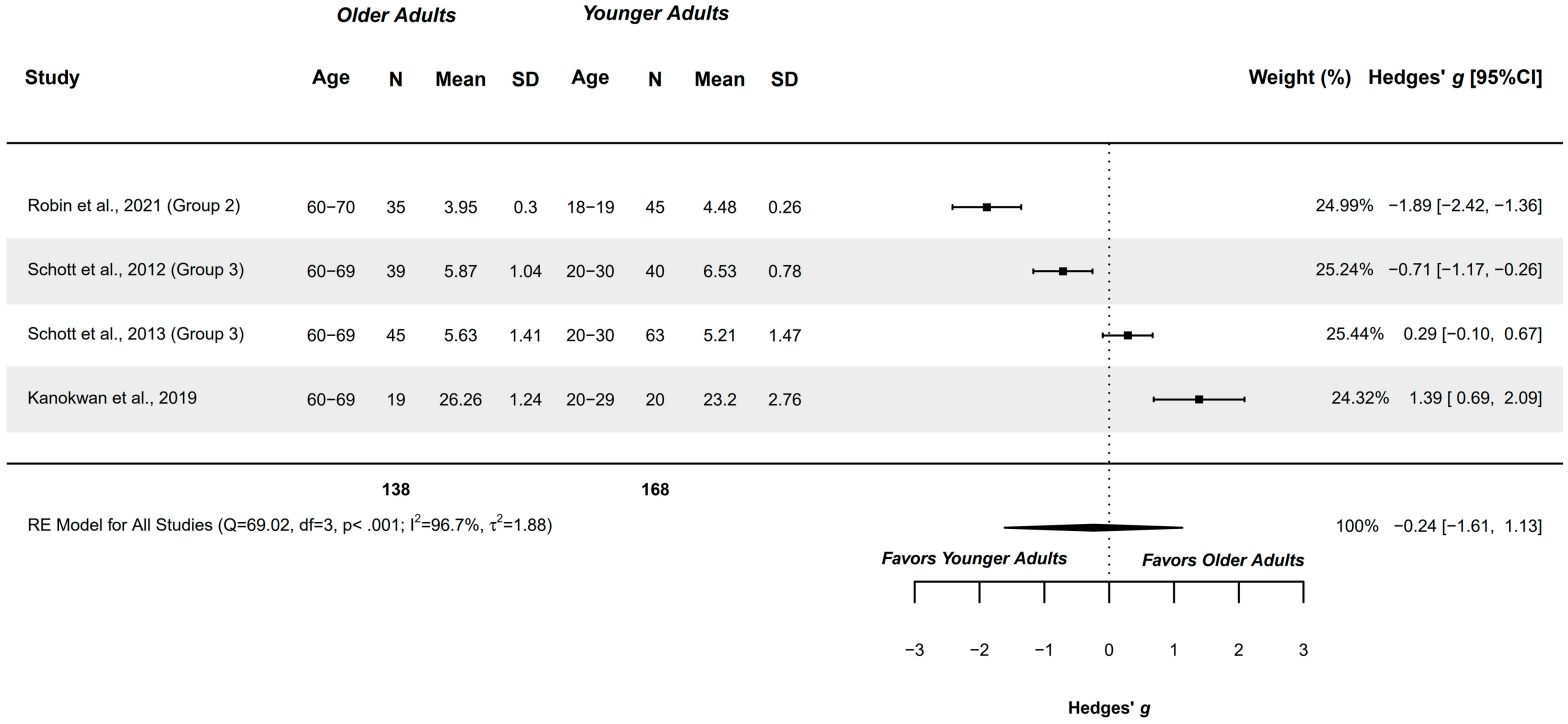
Meta-analysis: Ability to generate MI in kinesthetic modality in healthy older adults aged 60–70 years compared to healthy younger adults aged 18–30 years.

The meta-analysis revealed a non-significant small difference (*g =* −0.240; 95%CI = −1.611, 1.130), with 95%CI showing a large imprecision, considering that the capacity could range between large in favor of older adults, and large in favor of younger adults, preventing stablishing clear conclusions of groups’ difference. Current findings prevent drawing clear conclusions. The heterogeneity was significant (*Q =* 69.017, *p <* 0.001; *I*^2^ = 96.71%; *τ*^2^ = 1.883; see Figure 2). All studies were outliers in the funnel plot. Publication and selection bias were confirmed through asymmetry in the Doi plot (LFK = −1.09), but not with Egger’s regression test (*p =* 0.918). The sensitivity analysis did not reveal a significant influence of any study on the pooled result.

#### 3.4.2 Ability to generate MI – kinesthetic modality – older adults aged 70–82 years

Three studies explored this outcome measure in healthy older adults of 70–82 years, being compared with healthy younger adults aged 18–30 years (25, 77, 78). These studies were included in the meta-analysis, presenting a methodological quality of 4–6 points.

**Figure 3:**
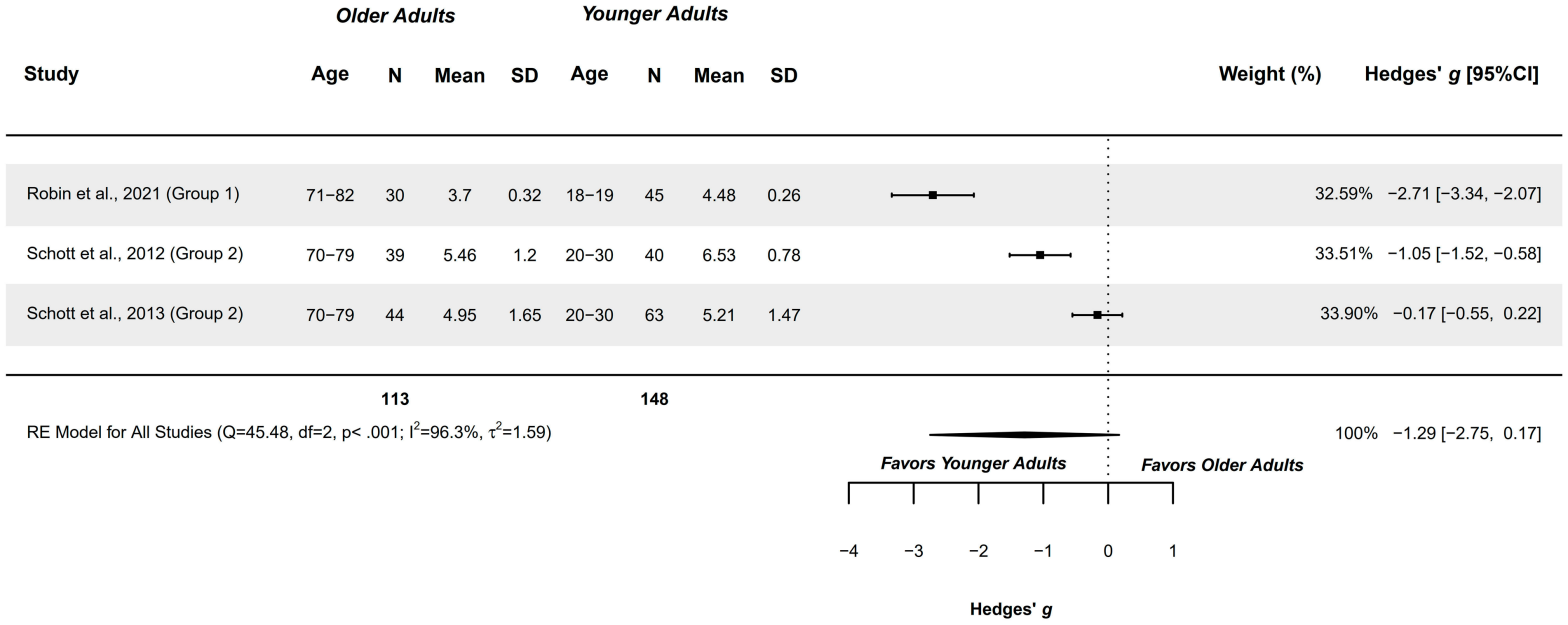
Meta-analysis: Ability to generate MI in kinesthetic modality in healthy older adults aged 70–82 years compared to healthy younger adults aged 18–30 years.

A non-significant difference was observed (*g =* −1.290; 95%CI = −2.748, 0.168), with 95%CI indicating that the capacity could range between large in favor of younger adults, or very small in favor of older adults. An imprecise, but observable tendency can be drawn from these findings in favor of younger adults. The heterogeneity was significant (*Q =* 45.479, *p <* 0.001; *I*^2^ = 96.30%; *τ*^2^ = 1.594; see Figure 3). Two studies were outliers in the funnel plot (25, 78). Publication and selection bias were confirmed through asymmetry in the Doi plot (LFK = −4.12), and with Egger’s regression test (*p =* 0.004).

#### 3.4.3 Ability to generate MI – visual modalities

Three studies explored this outcome, and were included in the meta-analysis, comparing healthy older adults aged 60–69 years with healthy younger adults aged 20–30 years (63, 77, 78). Studies presented a methodological quality of 5–6 points.

**Figure 4:**
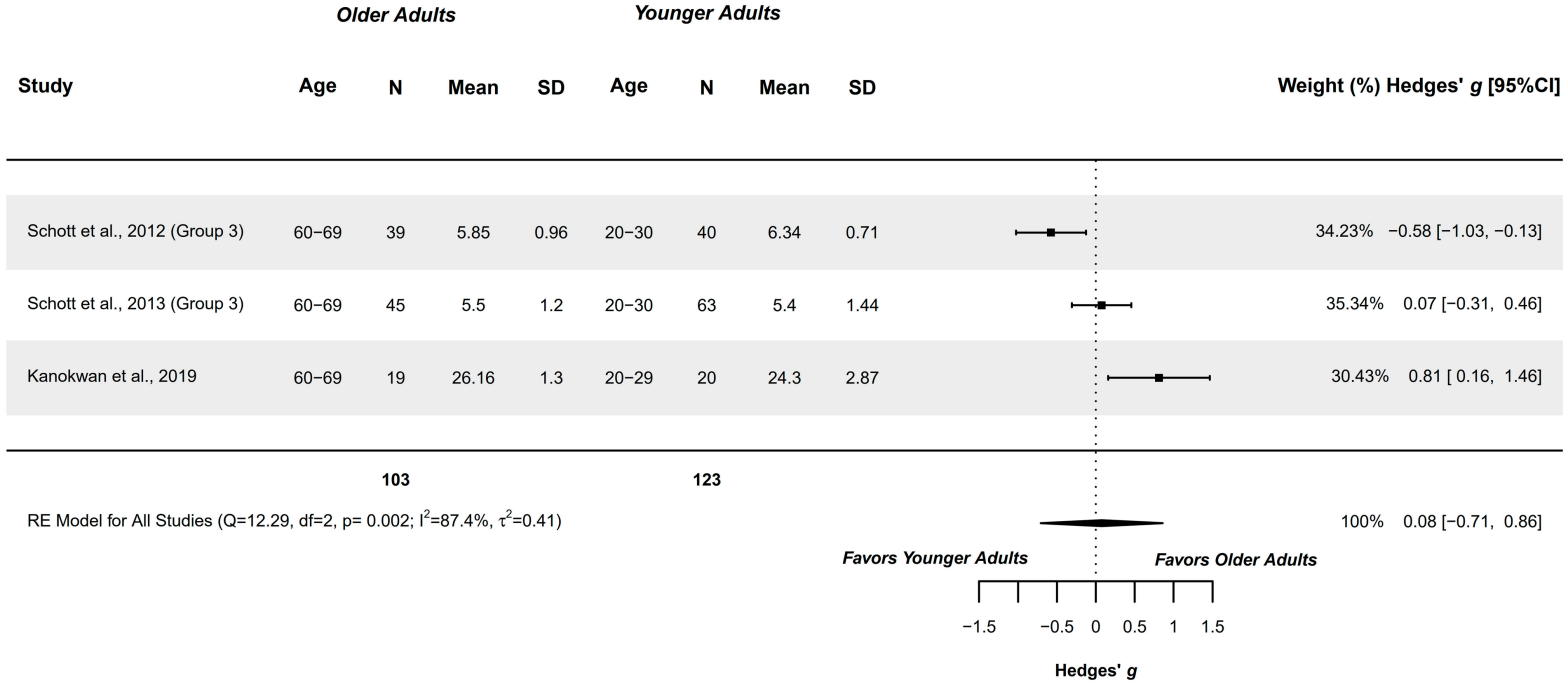
Meta-analysis: Ability to generate MI in visual modalities in healthy older adults aged 60–69 years compared to healthy younger adults aged 20–30 years.

A non-significant trivial difference was obtained (*g =* −0.076; 95%CI = −0.708, 0.859), with 95%CI showing imprecise findings, with the possibility of difference ranging between a moderate difference in favor of younger adults to a large difference in favor of older adults, preventing stablishing clear conclusions of the findings. The heterogeneity was significant (*Q =* 12.294, *p =* 0.002; *I*^2^ = 87.35%; *τ*^2^ = 0.414; see Figure 4). Two studies were outliers in the funnel plot (63, 77). Publication and selection bias confirmed with asymmetry in the Doi plot (LFK = 1.07), and not reaching significance in Egger’s regression test (*p =* 0.628).

#### 3.4.4 Vividness – kinesthetic modality

Four studies explored this outcome measure and were included in the meta-analysis. They compared healthy older adults aged 60–89 years with healthy younger adults aged 18–37 years (65, 66, 74, 75). Studies presented a methodological quality of 5–6 points.

**Figure 5:**
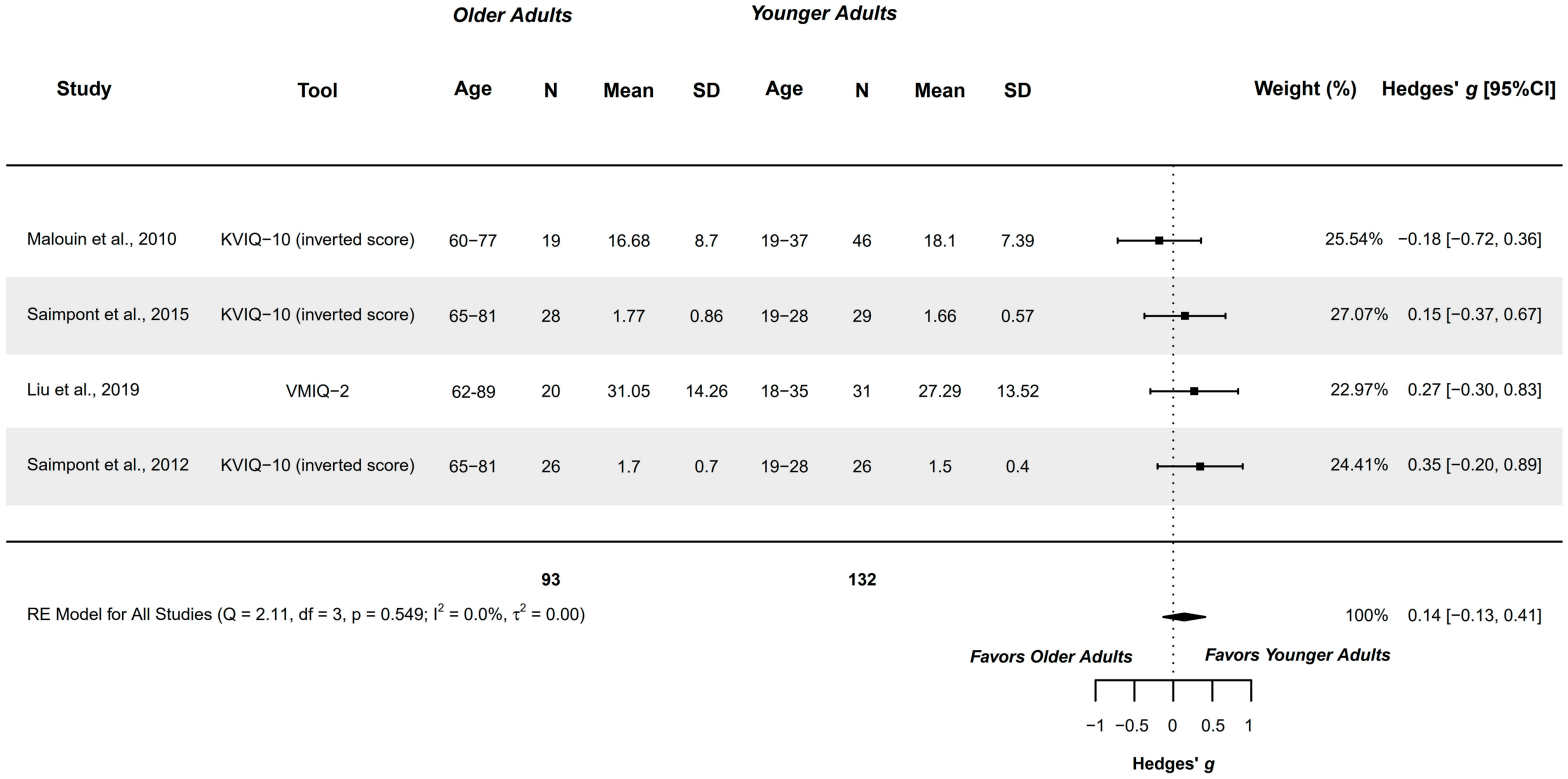
Meta-analysis: Vividness of MI in kinesthetic modality in healthy older adults aged 60–89 years compared to healthy younger adults aged 18–37 years.

A non-significant trivial difference was obtained (*g =* 0.140; 95%CI = −0.130, 0.411), with 95%CI indicating that the difference range between very small in favor of older adults, to small in favor of younger adults. Therefore, this capacity could be similar between groups. Heterogeneity was not significant (*Q =* 2.114, *p =* 0.549; *I*^2^ = 0%; *τ*^2^ = 0; see Figure 5). No outliers were identified in the funnel plot. Publication and selection bias was absent, as observed in the Doi plot (LFK = 0.09) and Egger’s regression test (*p =* 0.562). The sensitivity analysis did not reveal a significant influence of any study on the pooled result.

#### 3.4.5 Vividness – internal visual modality

Four studies explored this variable and were included in the meta-analysis. Studies compared healthy older adults aged 60–89 years with healthy younger adults aged 18–37 years (65, 66, 74, 75). Studies presented a methodological quality of 5–6 points.

**Figure 6:**
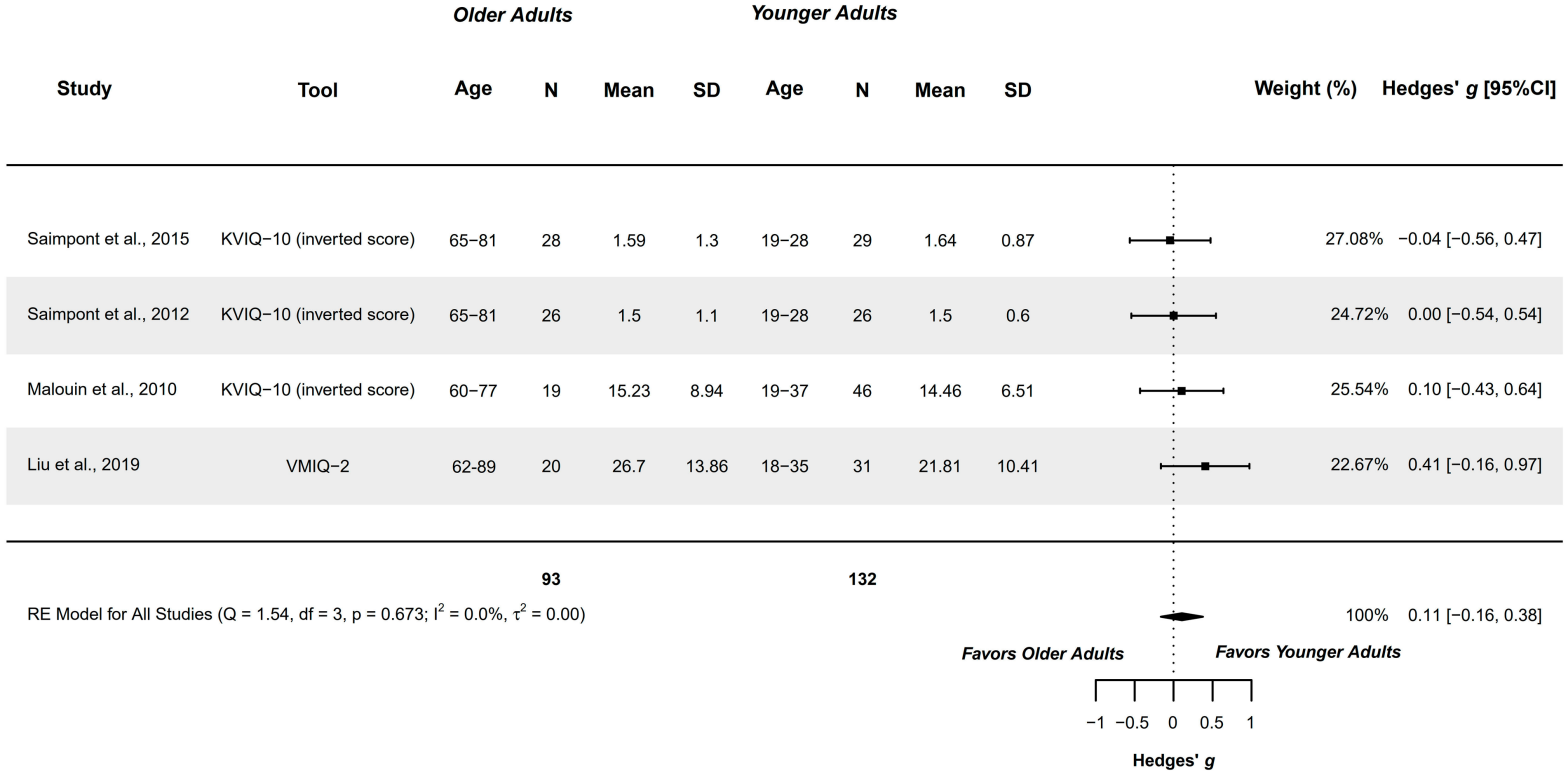
Meta-analysis: Vividness of MI in internal visual modality in healthy older adults aged 60–89 years compared to healthy younger adults aged 18–37 years.

A non-significant trivial difference was obtained (*g =* 0.107; 95%CI = −0.164, 0.377), with 95%CI indicating that the difference could range between very small in favor of older adults, to small in favor of younger adults. Therefore, this capacity could be similar between groups. Heterogeneity was not significant (*Q =* 1.541, *p =* 0.673; *I*^2^ = 0%; *τ*^2^ = 0; see Figure 6). No outliers were identified in the funnel plot. Publication and selection bias were confirmed with asymmetry in the Doi plot (LFK = 1.75), but not with Egger’s regression test (*p =* 0.111). The sensitivity analysis did not reveal a significant influence of any study on the pooled result.

#### 3.4.6 Vividness – external visual modality

Four studies explored this variable and were included in the meta-analysis. They analyzed healthy older adults aged 62–93 years compared with healthy younger adults aged 18–35 years (65, 68, 73, 79). Studies presented a methodological quality of 1–5 points.

**Figure 7:**
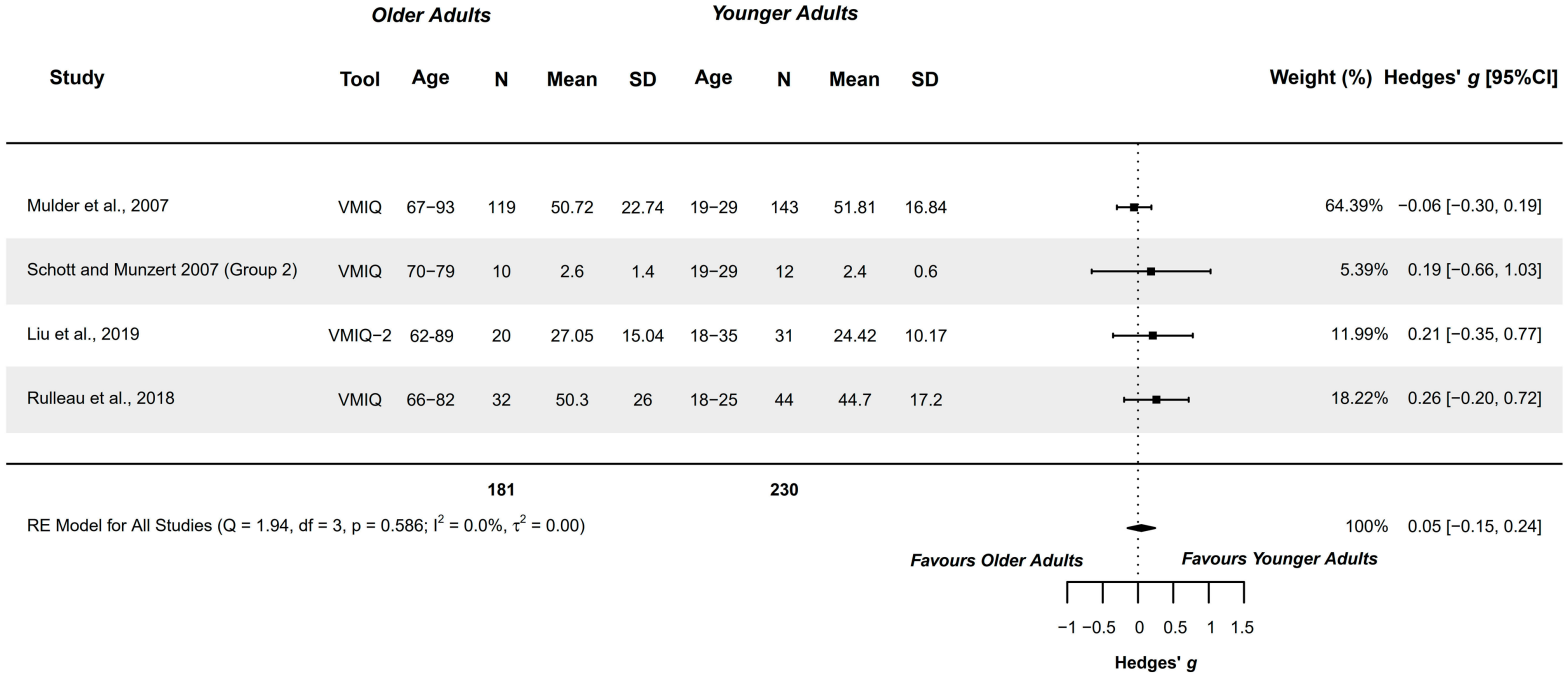
Meta-analysis: Vividness of MI in external visual modality in healthy older adults aged 62–93 years compared to healthy younger adults aged 18–35 years.

A trivial non-significant difference was obtained (*g =* 0.047; 95%CI = −0.148, 0.242), with 95%CI indicating that the difference could range between very small in favor of older adults, to small in favor of younger adults. Therefore, this capacity could be similar between groups. Heterogeneity was not significant (*Q =* 1.936, *p =* 0.586; *I*^2^ = 0%; *τ*^2^ = 0; see Figure 7). No outliers were identified in the funnel plot. Publication and selection bias were confirmed through asymmetry in the Doi plot (LFK = 5.83), but not through Egger’s regression test (*p =* 0.178). The sensitivity analysis did not reveal a significant influence of any study on the pooled result.

#### 3.4.7 Temporal features of MI (mental chronometry) – timed up and go test

Three studies exploring this outcome measure, in terms of mental chronometry (time), were included in the meta-analysis. Healthy older adults aged 70–87 years were compared with healthy younger adults aged 20–58 years (59, 77, 78). Studies presented a methodological quality of 6 points.

**Figure 8:**
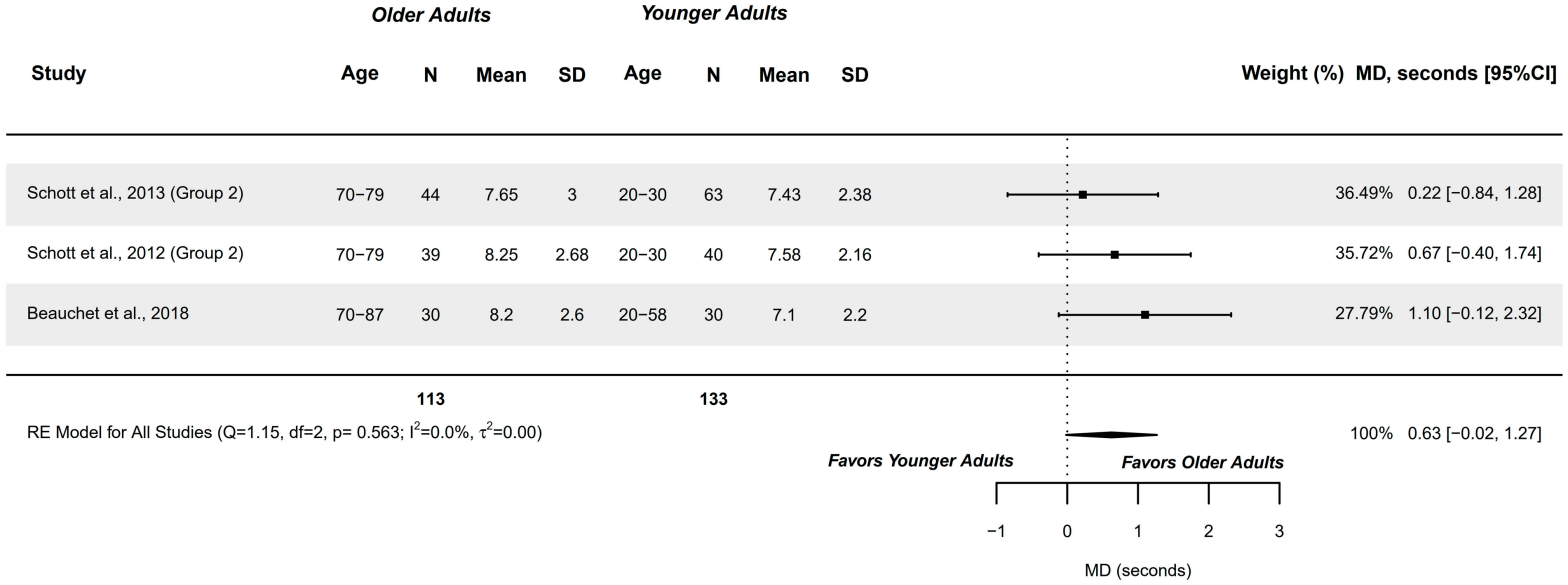
Meta-analysis: Mental chronometry in Timed-Up and Go test in healthy older adults aged 70–87 years compared to healthy younger adults aged 20–58 years.

A non-significant moderate difference was observed (MD, seconds = 0.625; 95%CI = −0.017, 1.268), with 95%CI indicating that the capacity could range between a similar between groups, to small difference in favor of older adults. An imprecise, but observable tendency can be drawn from these findings with older adults tending to require greater time. Heterogeneity was not significant (*Q =* 1.147, *p =* 0.563; *I*^2^ = 0%; *τ*^2^ = 0; see Figure 8). No outliers were identified in the funnel plot. Publication and selection bias were absent, with observed symmetry in the Doi plot (LFK = 0.42) and absent in Egger’s regression test (*p =* 0.321).

#### 3.4.8 Temporal features of MI (mental chronometry) – linear walk (5–10 m)

Five studies were meta-analyzed (64, 74, 80, 82, 83) from the eight studies exploring this variable (64, 71, 74, 77, 79, 80, 82, 83). Included studies compared healthy older adults aged 60–82 years with healthy younger adults aged 18–30 years, presenting a methodological quality of 1–6 points.

**Figure 9:**
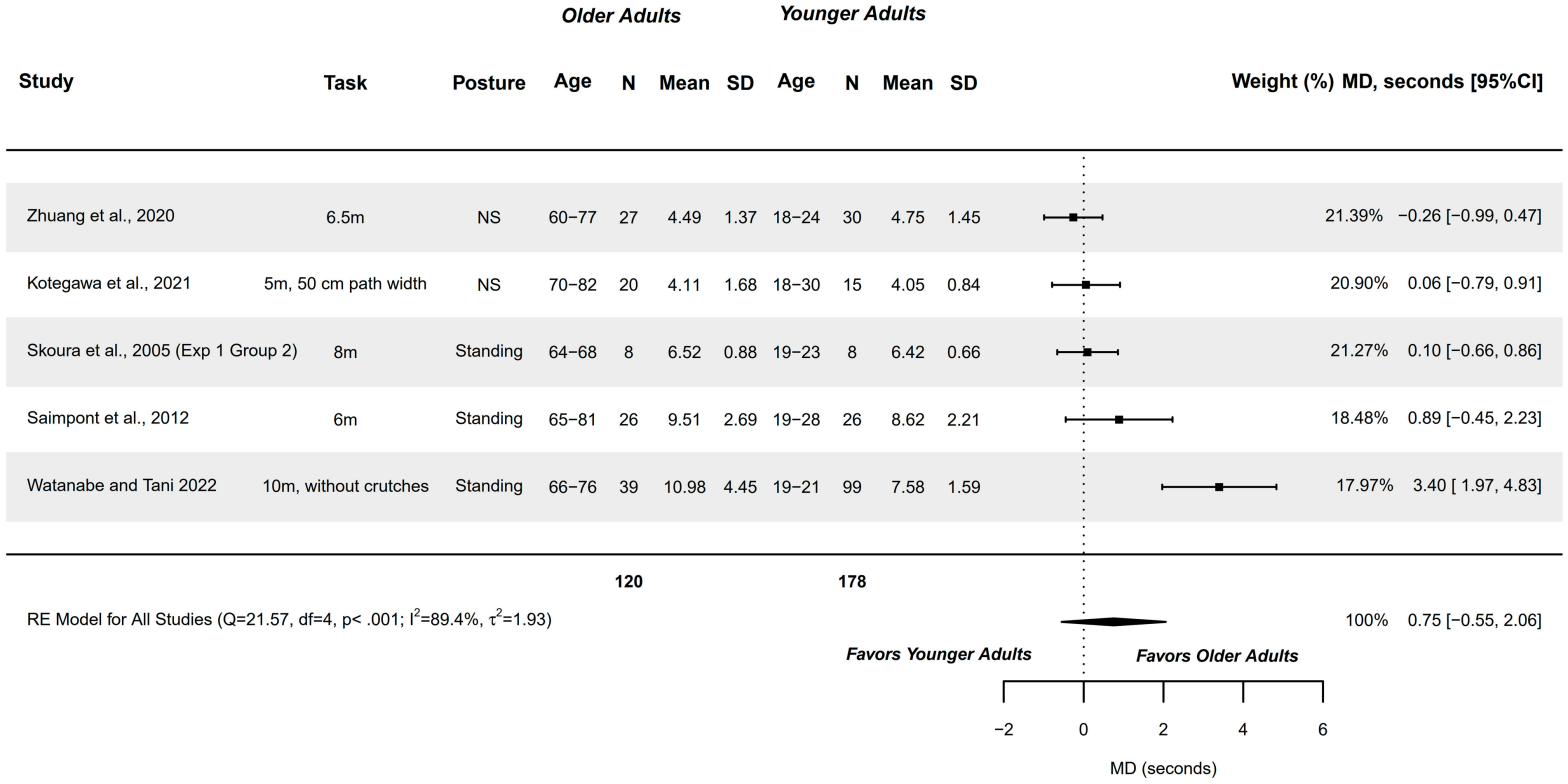
Meta-analysis: Mental chronometry in Linear Walk (5-10 m) in healthy older adults aged 60–82 years compared to healthy younger adults aged 18–30 years.

A non-significantly moderate difference was observed (MD, seconds = 0.754; 95%CI = −0.552, 2.059), with 95%CI indicating that differences could range between trivial to relevant differences in favor of older adults. Although these findings were imprecise, an observable tendency could be stablished from these findings, with older adults tending to require greater times. Heterogeneity was significant (*Q =* 21.574, *p <* 0.001; *I*^2^ = 89.39%; *τ*^2^ = 1.935; see Figure 9). Two outliers were identified in the funnel plot (82, 83). Publication and selection bias were confirmed with asymmetry in the Doi plot (LFK = 3.53), but not through Egger’s regression test (*p =* 0.054). The sensitivity analysis did not reveal a significant influence of any study on the pooled result.

#### 3.4.9 Temporal features of MI (mental chronometry) – UL tasks

**Figure 10:**
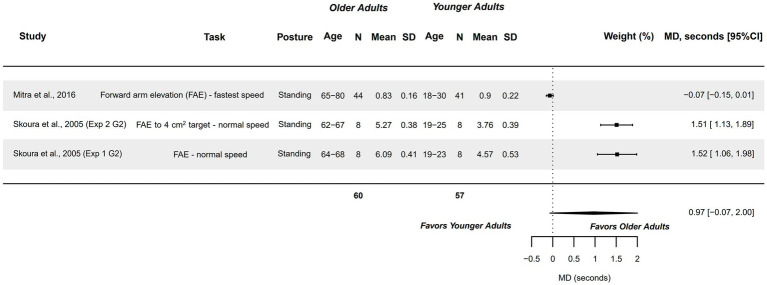
Visual forest plot: Mental chronometry in UL tasks (forward arm elevation task) in healthy older adults aged 62–80 years compared to healthy younger adults aged 18–30 years.

Two studies explored this variable in forward arm elevation task, and were analyzed visually through a forest plot, as they did not fulfill meta-analysis criteria (67, 80). They compared a sample of healthy older adults (62–80 years) with healthy younger adults (18–30 years). Studies presented a methodological quality of 4–5 points. An observable but not significant tendency was detected with older adults presenting greater mental chronometry time than younger adults (see Figure 10).

#### 3.4.10 MI-execution temporal congruence (performance overestimation) – linear walk (5–10 m)

Fron the seven studies exploring MI-execution temporal congruence, only five were meta-analyzed (62, 64, 74, 82, 83). Performance underestimation measures were transformed to overestimation measures for inclusion in the meta-analysis. Studies compared healthy older adults (60–82 years) with healthy younger adults (18–35 years) presenting a methodological quality of 1–6 points.

**Figure 11:**
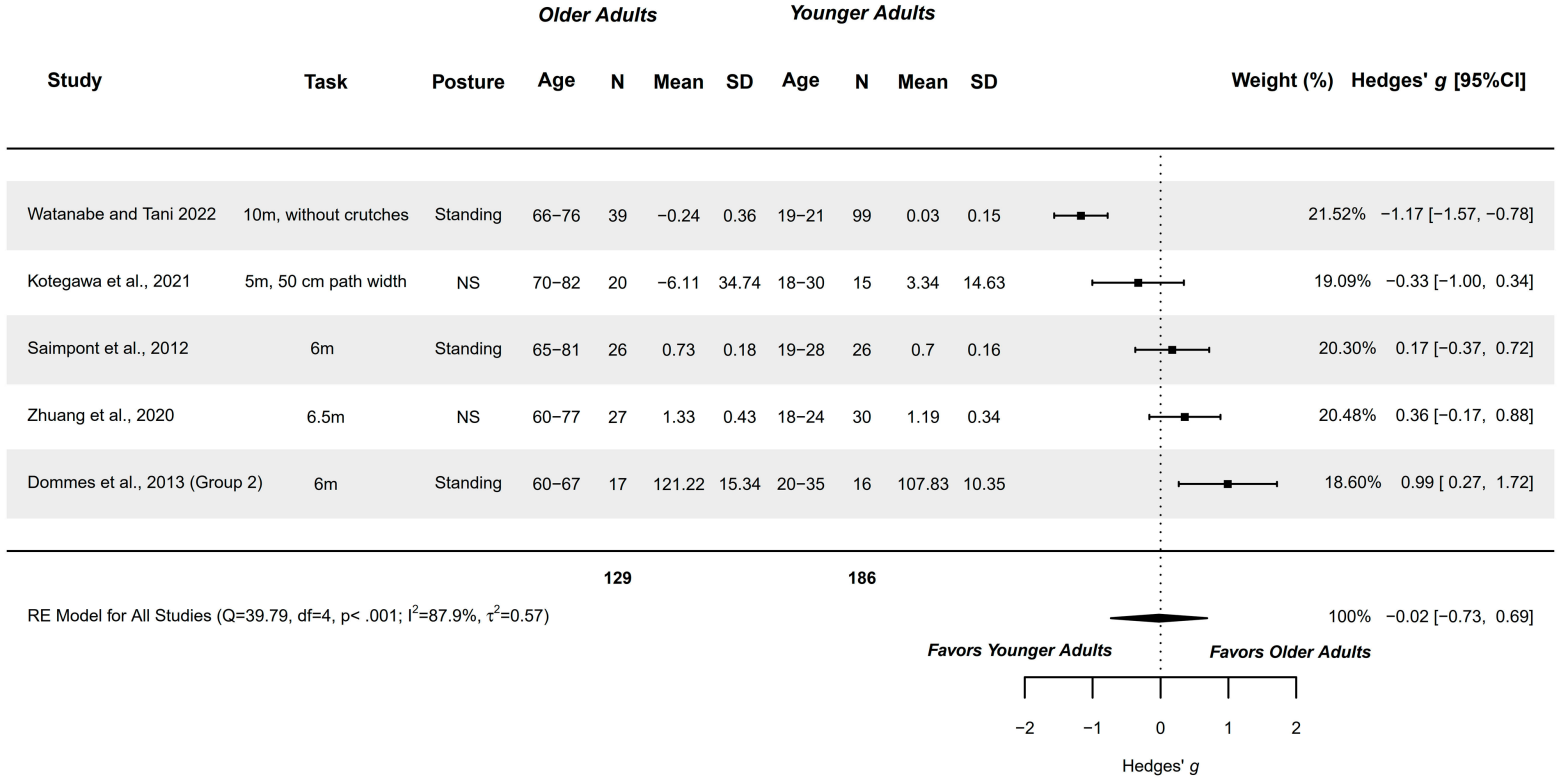
Meta-analysis: MI-execution temporal congruence (performance overestimation) in Linear Walk (5–10 m) in healthy older adults aged 60–82 years compared to healthy younger adults aged 18–35 years.

A non-significant trivial difference was obtained (*g =* −0.022; 95%CI = −0.731, 0.687), with 95%CI indicating imprecise findings, with overestimations in linear walk ranging from moderate in favor of younger adults to a moderate difference in favor of older adults. The heterogeneity was significant (*Q =* 39.788; *p <* 0.001; *I*^2^ = 87.88%; *τ*^2^ = 0.568; see Figure 11). Two studies were outliers in the funnel plot (62, 82). Publication and selection bias were confirmed with asymmetry in the Doi plot (LFK = 2.57), but not through Egger’s regression test (*p =* 0.107). The sensitivity analysis did not reveal a significant influence of any study on the pooled result.

#### 3.4.11 Hand recognition – accuracy

Two studies analyzed hand recognition accuracy (70, 72) and were included in the forest plot visual analysis, as they did not fulfill meta-analysis criteria. These studies presented a methodological quality score of 5–6 points. Specific rotations were explored visually in the forest plots with only 2 studies (61, 81) from the originally 3 available studies (61, 76, 81). They explored accuracy in 0°, 30°, 45°, 60°, 90°, 120°, 135°, 150°, and 180°, presenting a methodological quality of 1–2 points.

**Figure 12:**
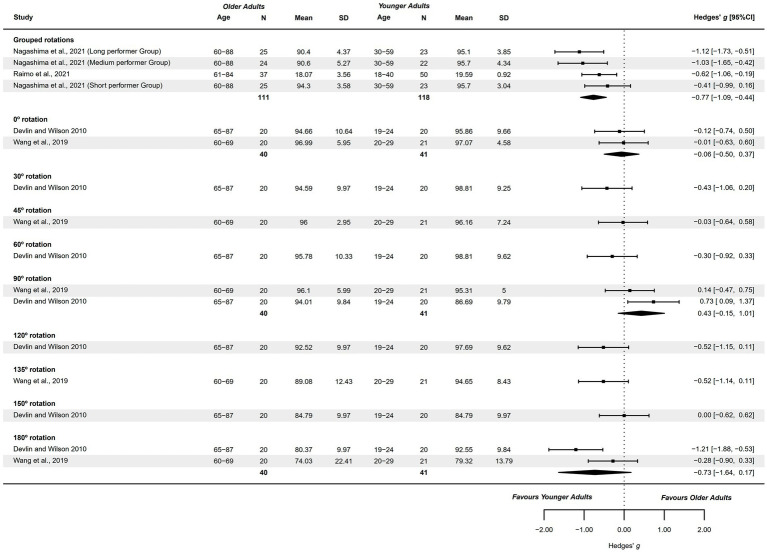
Visual forest plot: Implicit MI – Hand recognition accuracy in hand laterality judgement task in healthy older adults compared to healthy younger adults.

An observable and significant tendency was detected for accuracy in HLJ tasks grouping rotations, with younger adults presenting greater accuracy. This difference was not relevant in specific HLJ rotations. Accuracy at 0°, 45° and 150° no differences were observable, tendencies for younger adults presenting greater accuracy was observable at 30°, 60°, 120°, 135°, and 180°. A tendency for greater accuracy in older adults was observed at 90° (see Figure 12).

#### 3.4.12 Hand recognition – response time

Only one study exploring response time in HLJ task grouping rotations was finally included for visual analysis in the forest plot (70). The study presented a methodological quality of 5 points. Specific rotations was originally explored in 4 studies (61, 69, 76, 81), of which only 1 was included for visual analysis in the forest plot (61), analyzing specific rotations at 0°, 30°, 60°, 90°, 120°, 150°, and 180°. This study presented 2 points of methodological quality.

**Figure 13:**
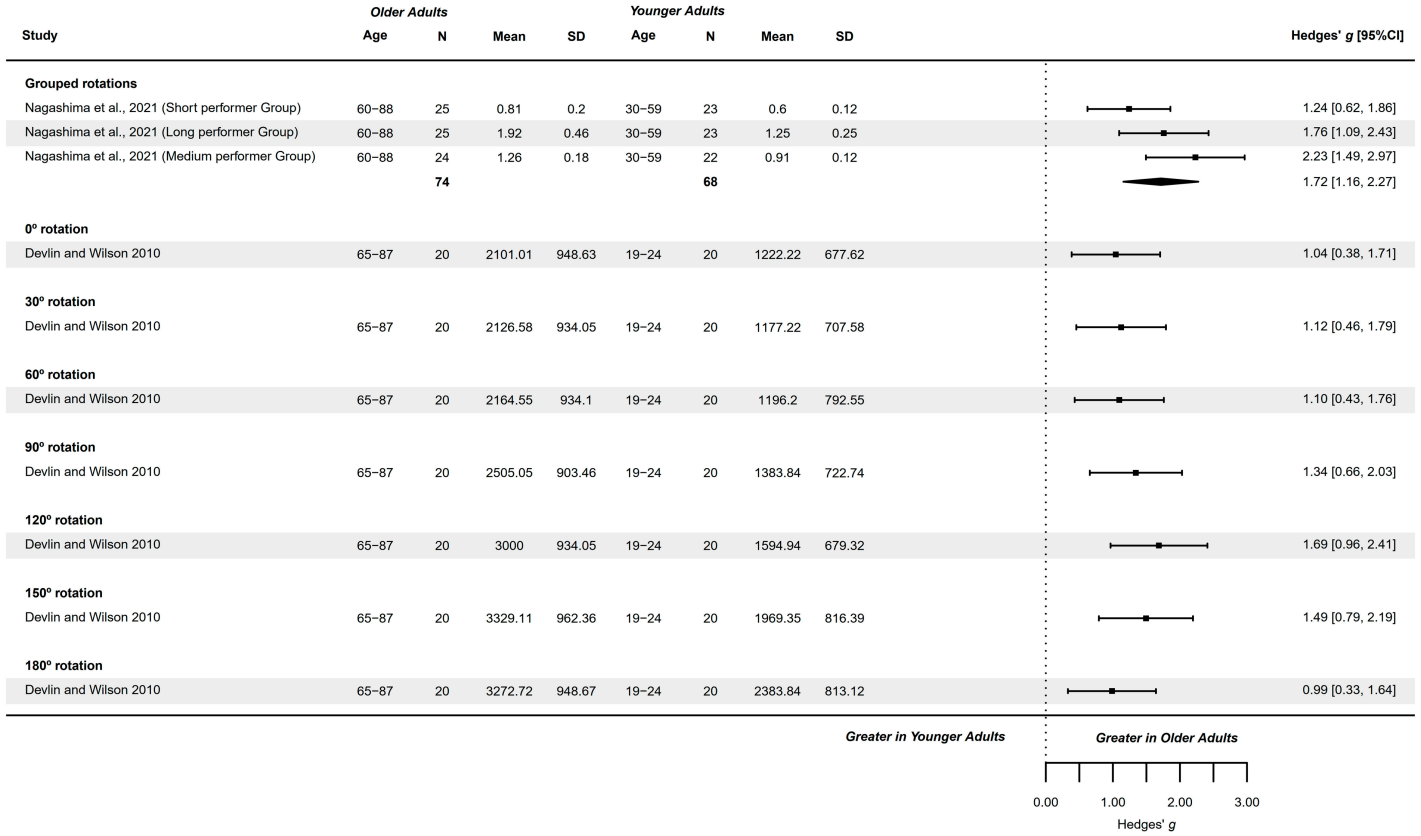
Visual forest plot: Implicit MI – Hand recognition response time in hand laterality judgement task in healthy older adults compared to healthy younger adults.

An observable and significant tendency was detected for response time in HLJ tasks with older adults presenting greater response time, analyzing grouped rotations and individual specific rotations (see Figure 13).

#### 3.4.13 Hand recognition – efficiency

One study explored this variable and was analyzed visually in the forest plot, as the outcome measure did not fulfill meta-analysis criteria (70). The study explored efficiency in hand recognition for back-view and palm-view medial and lateral rotations, presenting a methodological quality of 5 points.

**Figure 14:**
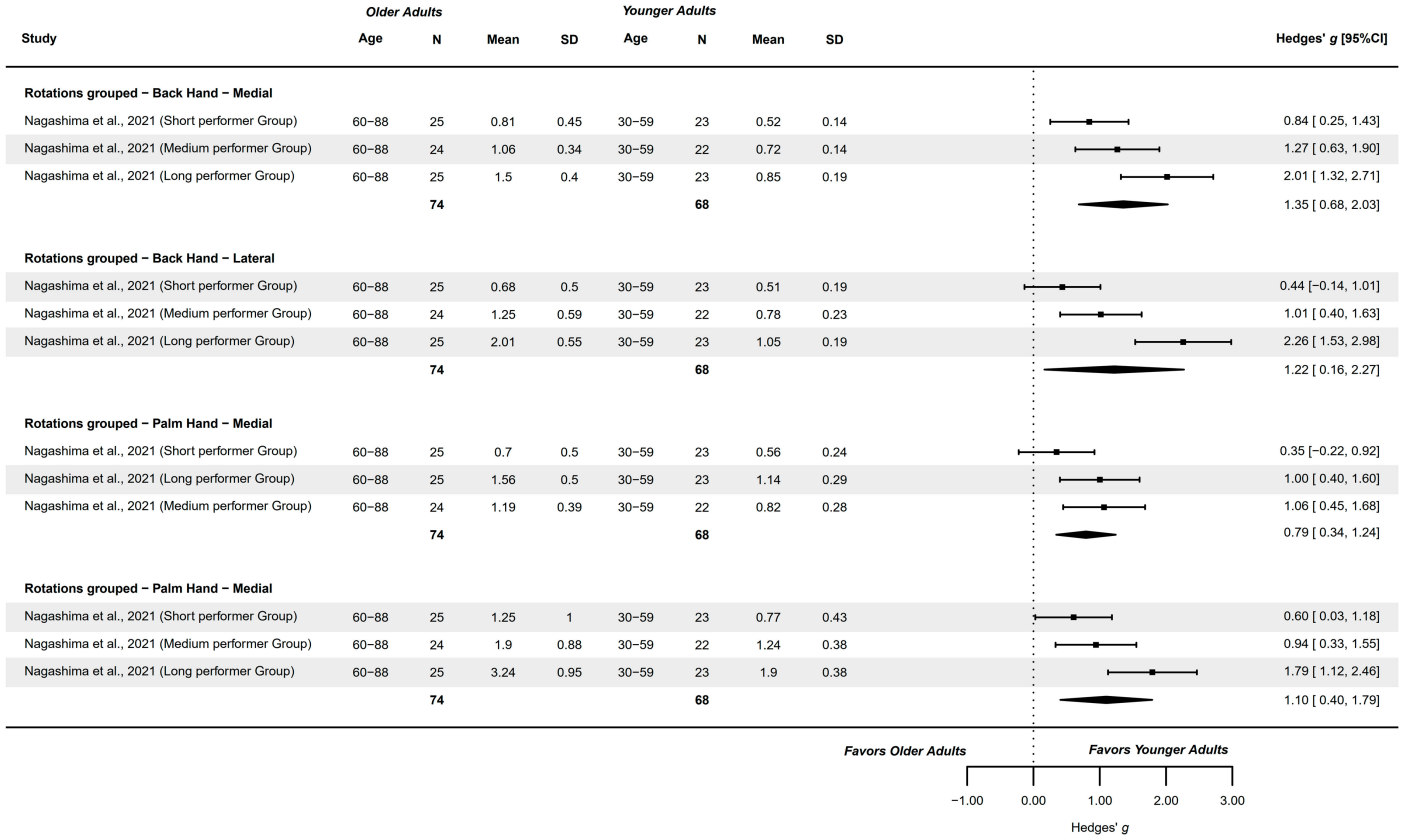
Visual forest plot: Implicit MI – Hand recognition efficiency in hand laterality judgement task in healthy older adults compared to healthy younger adults.

An observable and significant tendency was detected for efficiency in HLJ tasks with younger adults presenting greater efficiency across views (back and palm) and medial and lateral rotations (see Figure 14).

Meta-analysis summary results are shown in Table 4. Funnel and Doi plots are presented in Supplementary material.

**Table 4:** Summary information of all meta-analyses performed for MI capacities between healthy older and younger adults.

Meta–analyzed outcome measures												
Quantitative synthesis												Discussion
Studies (*p. comp*)		Method. quality	OA age (n)	YA age (n)	Difference (95%CI)		Heterog.	F. outliers *(p. comp)*	Pub. and Sel. bias	Leave–One–Out		Authors’ conclusions
										Influence (k)	Extraction results	
Capacity to generate MI – kinaesthetic modality												
4 *(4)*		4–6	60–70 years (*n* = 138)	18–30 years (*n* = 168)	Hedges’ *g*	−0.24 (−1.61, 1.13)	Significant	Yes *(4)*	Significant	NS	–	OA ≈ YA, but imprecise
3 *(3)*		4–6	70–82 years (*n* = 113)	18–30 years (*n* = 148)	Hedges’ *g*	−1.29 (−2.75, 0.17)	Significant	Yes *(2)*	Significant	–	–	OA ≈ YA, tending to OA < YA
Capacity to generate MI – visual modalities												
3 *(3)*		5–6	60–69 years (*n* = 103)	20–30 years (*n* = 123)	Hedges’ *g*	−0.08 (−0.71, 0.86)	Significant	Yes *(2)*	Significant	–	–	OA ≈ YA, but imprecise
Vividness – kinaesthetic modality												
4 *(4)*		5–6	60–89 years (*n* = 93)	18–37 years (*n* = 132)	Hedges’ *g*	0.14 (−0.13, 0.41)	NS	No	NS	NS	–	OA ≈ YA, and precise
Vividness – internal visual modality												
4 *(4)*		5–6	60–89 years (*n* = 93)	18–37 years (*n* = 132)	Hedges’ *g*	0.11 (−0.16, 0.38)	NS	No	Significant	NS	–	OA ≈ YA, and precise
Vividness – external visual modality												
4 *(4)*		1–5	62–93 years (*n* = 181)	18–35 years (*n* = 230)	Hedges’ *g*	0.05 (−0.15, 0.24)	NS	No	Significant	NS	–	OA ≈ YA, and precise
Temporal features of MI (mental chronometry) – TUG												
3 *(3)*		6	70–87 years (*n* = 113)	20–58 years (*n* = 133)	MD (s)	0.63 (−0.02, 1.27)	NS	No	NS	–	–	OA ≈ YA, tending to OA > YA
Temporal features of MI (mental chronometry) – Linear Walk (5–10 m)												
5 *(5)*		1–6	60–82 years (*n* = 120)	18–30 years (*n* = 178)	MD (s)	0.75 (−0.55, 2.06)	Significant	Yes *(2)*	Significant	NS	–	OA ≈ YA, tending to OA > YA
MI–execution temporal congruence (performance overestimation) – Linear Walk (5–10 m)												
5 *(5)*		1–6	60–82 years (*n* = 129)	18–35 years (*n* = 186)	Hedges’ *g*	−0.02 (−0.73, 0.69)	Significant	Yes *(2)*	Significant	NS	–	OA ≈ YA, but imprecise
Visually analyzed outcome measures												
Temporal features of MI (mental chronometry) – UL tasks												
2 *(3)*		4–5	62–80 years (*n* = 60)	18–30 years (*n* = 57)	MD (s)	0.97 (−0.07, 2.00)	n/a	n/a	n/a	n/a	–	OA ≈ YA, tending to OA > YA
Hand recognition – accuracy												
Grouping rot.	2 *(4)*	5–6	60–88 years (*n* = 111)	18–59 years (*n* = 118)	Hedges’ *g*	−0.77 (−1.09, −0.44)	n/a	n/a	n/a	n/a	–	OA < YA
0° rot.	2 *(2)*	1–2	60–87 years (*n* = 40)	19–29 years (*n* = 41)	Hedges’ *g*	−0.06 (−0.50, 0.37)	n/a	n/a	n/a	n/a	–	OA ≈ YA
30° rot.	1 *(1)*	2	65–69 years (*n* = 20)	19–24 years (*n* = 20)	Hedges’ *g*	−0.43 (−1.06, 0.20)	n/a	n/a	n/a	n/a	–	OA < YA
45° rot.	1 *(1)*	1	60–69 years (*n* = 20)	20–29 years (*n* = 21)	Hedges’ *g*	−0.03 (−0.64, 0.58)	n/a	n/a	n/a	n/a	–	OA ≈ YA
60° rot.	1 *(1)*	2	65–87 years (*n* = 20)	19–24 years (*n* = 20)	Hedges’ *g*	−0.30 (−0.92, 0.33)	n/a	n/a	n/a	n/a	–	OA < YA
90° rot.	2 *(2)*	1–2	60–87 years (*n* = 40)	19–29 years (*n* = 41)	Hedges’ *g*	0.43 (−0.15, 1.01)	n/a	n/a	n/a	n/a	–	OA > YA
120° rot.	1 *(1)*	2	65–87 years (*n* = 20)	19–24 years (*n* = 20)	Hedges’ *g*	−0.52 (−1.15, 0.11)	n/a	n/a	n/a	n/a	–	OA < YA
135° rot.	1 *(1)*	1	65–69 years (*n* = 20)	20–29 years (*n* = 21)	Hedges’ *g*	−0.52 (−1.14, 0.11)	n/a	n/a	n/a	n/a	–	OA < YA
150° rot.	1 *(1)*	2	65–87 years (*n* = 20)	19–24 years (*n* = 20)	Hedges’ *g*	0.00 (−0.62, 0.62)	n/a	n/a	n/a	n/a	–	OA ≈ YA
180° rot.	2 *(2)*	1–2	60–87 years (*n* = 111)	19–29 years (*n* = 118)	Hedges’ *g*	−0.73 (−1.64, 0.17)	n/a	n/a	n/a	n/a	–	OA < YA
Hand recognition – response time												
Grouping rot.	1 *(3)*	5	60–88 years (*n* = 74)	30–59 years (*n* = 68)	Hedges’ *g*	1.72 (1.16, 2.27)	n/a	n/a	n/a	n/a	–	OA > YA
0° rot.	1 *(1)*	2	65–87 years (*n* = 20)	19–24 years (*n* = 20)	Hedges’ *g*	1.04 (0.38, 1.71)	n/a	n/a	n/a	n/a	–	OA > YA
30° rot.	1 *(1)*	2	65–87 years (*n* = 20)	19–24 years (*n* = 20)	Hedges’ *g*	1.12 (0.46, 1.79)	n/a	n/a	n/a	n/a	–	OA > YA
60° rot.	1 *(1)*	2	65–87 years (*n* = 20)	19–24 years (*n* = 20)	Hedges’ *g*	1.10 (0.43, 1.76)	n/a	n/a	n/a	n/a	–	OA > YA
90° rot.	1 *(1)*	2	65–87 years (*n* = 20)	19–24 years (*n* = 20)	Hedges’ *g*	1.34 (0.66, 2.03)	n/a	n/a	n/a	n/a	–	OA > YA
120° rot.	1 *(1)*	2	65–87 years (*n* = 20)	19–24 years (*n* = 20)	Hedges’ *g*	1.69 (0.96, 2.41)	n/a	n/a	n/a	n/a	–	OA > YA
150° rot.	1 *(1)*	2	65–87 years (*n* = 20)	19–24 years (*n* = 20)	Hedges’ *g*	1.49 (0.79, 2.19)	n/a	n/a	n/a	n/a	–	OA > YA
180° rot.	1 *(1)*	2	65–87 years (*n* = 20)	19–24 years (*n* = 20)	Hedges’ *g*	0.99 (0.33, 1.64)	n/a	n/a	n/a	n/a	–	OA > YA
Hand recognition – efficiency												
Back view – Medial rot.	1 *(3)*	5	60–88 years (*n* = 74)	30–59 years (*n* = 68)	Hedges’ *g*	1.35 (0.68, 2.03)	n/a	n/a	n/a	n/a	–	OA < YA
Back view – Lateral rot.	1 *(3)*	5	60–88 years (*n* = 74)	30–59 years (*n* = 68)	Hedges’ *g*	1.22 (0.16, 2.27)	n/a	n/a	n/a	n/a	–	OA < YA
Palm view – Medial rot.	1 *(3)*	5	60–88 years (*n* = 74)	30–59 years (*n* = 68)	Hedges’ *g*	0.79 (0.34, 1.24)	n/a	n/a	n/a	n/a	–	OA < YA
Palm view – Lateral rot.	1 *(3)*	5	60–88 years (*n* = 74)	30–59 years (*n* = 68)	Hedges’ *g*	1.10 (0.40, 1.79)	n/a	n/a	n/a	n/a	–	OA < YA

Comp., comparisons; F. outliers, funnel plot outliers; LL, lower limb; MC, mental chronometry; MD, mean difference; Method., methodological; Mod-good, moderate-to-good; n/a, not analyzed; NOS, Newcastle Ottawa Scale; OA, older adults; P. comp., pairwise comparisons; Pub. and Sel. bias, publication and selection bias; Seated knee ext., seated knee extensions; TUG, timed-up-and-go test; UL, upper limb; YA, younger adults.

## 4 Discussion

The purpose of this meta-analysis was to evaluate the differences in MI abilities between older and younger adults. MI assessments included the ability to generate KI and visual MI, the vividness of MI across KI, IV, and EV modalities, mental chronometry, the synchrony of MI and execution time, and hand recognition accuracy, response time and efficiency.

Discrepant results were observed across variables. Results for the capacity of generating MI were mainly imprecise. However, the most consistent results were pooled from the MI vividness measure, with meta-analyses for KI, IV and EV showing with precision that older and younger adults presented similar vividness. Mental chronometry for TUG and linear walk, showed a tendency for older adults to require greater times. Performance overestimation in linear walk presented imprecise results. Hand recognition accuracy was diminished in older adults (grouping rotations), their response time was greater, added to a lower efficiency than younger adults.

### 4.1 Capacity for generating kinesthetic and visual MI

Kinesthetic MI entails the mental simulation of movement without its actual execution, focusing on the haptic sensations experienced during real movement, such as tactile, proprioceptive, and KI feedback (85, 86). A theory has been proposed suggesting KI MI is rooted in the internal activation of anticipatory representations of the action’s effects. This mechanism could potentially facilitate enhanced motor performance through an internal emulation of the action, obviating the need for physical execution (87).

KI MI is considered a complex process, intimately linked to prior movement experience (88) and has been proposed to be more complex than visual MI. This difference might be attributed to the requirement of reactivating multiple perceptions that are typically present during physical movement, perceptions that are ordinarily not consciously attended. Additionally, these perceptions demands a high level of cognitive availability (89).

Two meta-analyses were conducted for the capacity of geniting kinesthetic MI, analyzing two age groups. Their results were mainly imprecise, not being able to stablish clear conclusions about the difference between older and younger adults. However a tendency for presenting a lower capacity was observed in the meta-analysis which included elder older adults (70–82 years).

Additionally, one meta-analysis was performed with older adults of 60–70 years for the ability to generate visual MI, also presenting a relevant imprecision.

Selection criteria for meta-analyses were well-stablished including homogeneous populations, and outcome measures. The population tested comprised healthy older adults (with some studies establishing cognitively healthy cut-offs), which would explain the absence of differences between groups. For example, pathological populations such as patients with spinal cord injuries present greater difficulties for the construction of both KI sensations and visual images during MI compared with healthy individuals (90).

We acknowledge the imprecision of the results on the between-study variability. There could be probably cultural differences that stress the differences between studies, as observed from heterogeneity results and the presence of outliers in the funnel plot. In fact, some studies detected that older adults presented a lower capacity, others revealed similar capacities, and another a higher capacity. Further studies should explore this concern, potentially changing the actual findings.

### 4.2 Vividness of MI

In this meta-analysis, no relevant differences were found in the evaluation of MI vividness in KI, IV, and EV modalities, between younger (18–37 years) and older (60–89 years) adults. We consider that these meta-analyses were the most consistent and precise, with 4 studies included in each meta-analysis, with a methodological quality of 5–6 points in JBI, and with a marked absence of heterogeneity. These results were not changed with leave-one-out analysis. Therefore, clear conclusions can be extracted from these results. However, the reader should be concerned that there were a low amount of studies meta-analyzed, so the present findings could be changed in the future.

High vividness in MI ensures that the imagery retains a strong resemblance to actual movement. It has been studied that vivid motor images enhances neural networks responsible for movement control, facilitating motor learning and brain plasticity (91). Vividness can be preserved under certain conditions even in the context of disease, as healthy and early-stage patients with Parkinson’s disease present similar vividness in (92).

Higher vividness has been associated to lower brain activation compared to those of poor imagers, which could be due to the compensatory activation of executive regions with potential to drive the imagery process (93).

A recent study conducted in a population of 18–60 years reported that the vividness of visual images significantly decreases with aging (94), and this aspect has also been observed in the older adult population in a study which, due to inclusion criteria, was not part of the meta-analysis (95).

However, vividness represents an additional cognitive dimension in MI and does not directly imply an influence on motor performance (96). Previous studies have suggested that the level of imagery vividness is not precisely associated with various aspects related to motor performance (95, 97) or with the ability to generate specific MI (95). Some factors may influence vividness preservation in aging, such as familiarity with the task, task-specific focus and the engagement of compensatory neural mechanisms (98–100).

Looking ahead, it is advisable for future research to focus on determining whether the vividness of MI among young and older adults varies depending on the complexity of the movement or in relation to other factors such as the difference between movements of the upper and lower limbs.

### 4.3 Mental chronometry in timed up and go, linear walk, and forward arm elevation task

The meta-analyses were imprecise; however, observable tendencies were identified across mental chronometry in TUG, linear walk (5–10 m), and forward arm elevation tasks, with older adults requiring greater time.

No significant heterogeneity was observed in the TUG meta-analysis, whereas it was present in the linear walk. This was probably derived from the variability of linear walk distances assessed between studies, ranging from 5 to 10 m. In fact, the study of Watanabe and Tani (82) assessing mental chronometry in 10 m walk task was a relevant outlier, potentially generating the observed heterogeneity. Authors should point out that the differences in mental chronometry could be compatible with an increase in execution time to perform that action, as a result of the process of aging.

Another point contributing into the observed heterogeneity is the difficulty of the imagined task. The evaluated tasks appeared to produce similar imagination times between older and younger adults, with a tendency to present greater differences with larger imagined linear walk distances. In fact, other studies have also reported trends that mental chronometry begins to differ between older and younger adults with greater age (79), and when the difficulty of the task increases, for example, with higher linear walk distances (19–20 m) (79, 83). Furthermore, prior evidence has suggested that brain activity varies in MI processes depending on the task’s difficulty or complexity (101), and MI capacity is known to be less affected in simple tasks than in complex tasks (102).

Therefore, the meta-analysis results should be interpreted with caution, as these influential variables may change the direction of results. Although an observable tendency is being observed in the present meta-analyses, it is probable that these values vary with greater age and more difficult tasks.

### 4.4 MI-execution temporal congruence (performance overestimation) in linear walk tasks

Performance over/underestimation referred to the process in which an individual imagined a task with an improved or reduced performance, respectively, to the actual performance of its execution. It is expected that individuals present similar temporal features during MI and execution; nevertheless, the process of aging and the difficulty of the task can imply a tendency to over/underestimate their performance. The meta-analysis results were mainly imprecise, avoiding the establishment of a clear direction of the results. The meta-analysis explored older adults (60–82 years) with younger adults (18–35 years) in linear walk tasks of 5–10 m, with 95%CI indicating that either older or younger adults could present large differences in over/underestimation of their performance. A great heterogeneity was observed between studies, accompanied with the presence of outliers in the funnel plot. As mentioned previously, it could be derived from the between-study variability of meta-analyzed tasks, with one outlier being the 10 m linear walk task (82).

Other studies have observed that MI-execution temporal congruence is affected by age (79, 103) and the complexity of the task (79, 82, 83).

The assessment of over/underestimation exposes the quality of movement planification, affecting the efficiency and safety of movement (104). Several studies have been conducted in older and younger adults, exploring their mental chronometry for crossing a street. This is a relevant situation in which both a greater over/underestimation of their performance could lead to an accident. Not only is performance over/underestimation influencing this process, but additionally, the reaction time to make the decision to cross the street. Although younger and older adults can both over/underestimate their performance, previous research has suggested that older adults greatly overestimate their performance compared with younger adults, leading to unsafe crossing decisions (62, 105).

Additionally, greater performance overestimations are present in older adults with fear of falling, additionally explaining the increased risk of falls or recurrent falls in this population (106, 107). Further research should explore the observed between-study heterogeneity as possible confounding factors, such as the cultural context, difficulty of the task (linear walk distance), and older ages could moderate these results.

### 4.5 Implicit MI through hand recognition

Implicit MI involves an internal process of mentally project and manipulate body schema (23). This process is implicit to any mental representation of movement, and participates simultaneously with explicit MI. In fact, both explicit and implicit MI activate similar neural networks associated with the somatosensory and motor regions (108).

Implicit MI was analyzed in terms of accuracy, response time, and efficiency on the ability to solve HLJ task. These variables did not fulfill criteria for meta-analysis, however, group differences were explored visually analyzing forest plots. Accuracy in HLJ revealed relevant results, with older adults presenting an observable reduction to accurately solve the task when grouping rotations. However, we hypothesize that this was not observed with specific rotations due to a lack of studies. Nevertheless, significant tendencies were observed in HLJ response time, with grouped, and individual rotations showing that older adults presented greater response time than younger adults. This was also observed with a reduced efficiency in the task.

Caution should be taken when extrapolating these results due to the limited number of studies analyzed. Nonetheless, these findings appear to be consistent with previous literature, which indicates that response time tends to increase with healthy aging (109). This increase is likely due to greater difficulty in processing stimuli and preparing movement rather than from an avoidance to respond (110).

Several investigations have examined whether HLJ performance is based on the ability to manipulate the body schema, or essentially on the ability to mentally rotate images. For such purpose, studies have compared the ability to identify rotated body images and unrelated body images, such as letters. Muto et al. (69), provided significant results addressing this question through response time performance. Older adults showed longer response times as a function of medial/lateral rotations of hand and foot images compared to younger adults, with response times increasing further with age. In contrast, older and younger adults exhibited similar response times for clockwise/counterclockwise rotations of letters. The same pattern occurred for the combination of medial/lateral and angular rotations of hand and foot images, while no differences were found between groups in response times for the interaction of clockwise/counterclockwise and angular rotation of letters.

### 4.6 Clinical implications

The results of the present meta-analysis project significant clinical implications, mainly derived from the findings on vividness and mental chronometry. These meta-analyses showed, respectively, a preservation of the vividness during MI, and a tendency to present greater times in mental chronometry in TUG, linear walk and upper limb tasks. Additionally, observable tendencies were identified for the ability to mentally manipulate body schema, with older adults presenting lower accuracy, longer response time and reduced efficiency. These observed changes may be attributed to healthy aging. No clear conclusions can be drawn from the ability to generate MI or MI-execution temporal congruence. We acknowledge that the observed heterogeneity, limiting a precise conclusion, on the ability to generate MI and MI-Execution temporal congruence, may be derive from sampling differences across studies, as robust selection criteria were employed for inclusion in meta-analyses.

While vividness during MI appears to remain preserved, the overall quality of MI may decline due to age-related reductions in visuospatial and kinesthetic working memory (66). Furthermore, the representation of the body schema seems impaired, alongside increased task imagination times. These changes align with the typical decrease in physical performance associated with aging. Rehabilitation efforts aimed at improving vividness could potentially enhance both working memory and functional performance (66, 111).

Exploring MI capacities in healthy aging is essential for determining whether older adults are suitable candidates for interventions based on MI. In this context, the ability to generate MI plays a critical role in the successful implementation of MI-based interventions. Although no clear conclusions could be drawn this outcome, this ability can be improved with MI training (112). Similarly, MI-execution temporal congruence can also be enhanced, as recent research indicates that the inclusion of external feedback improves the temporal synchrony between MI and execution (113). Although the variation of this outcome with aging remains unclear, these findings indicate that it is possible to develop improvements on it. In the case of implicit MI ability, the inclusion of its training, can produce improvements in postural balance and postural control (114).

MI have been proposed as useful therapeutic tools for physical and functional rehabilitation in older adults, showing specific benefits for strength development (34). Current evidence, though of low to very-low quality, supports the use of MI to improve balance, gait speed, and lower limb function in this population (115). Furthermore, MI training has demonstrated potential benefits for cognitive functions. Improvements have been reported in patients with stroke (116, 117), multiple sclerosis (118), Parkinson’s disease (119, 120), healthy middle-aged adults (121), healthy older adults (122) and older adults individuals with cognitive impairment (123–125).

MI training represents a safe and relatively simple strategy for reducing the risk of falls and other mobility-related incidents. For instance, MI interventions could be particularly beneficial in enhancing the estimation of body movements. This improvement could help older adults in daily activities, such as street-crossing, where misjudging crossing time may pose significant risks (126).

Researchers and clinicians should consider that MI abilities may vary based on an individual’s age and the complexity of the imagined task. Tailoring interventions to these factors may optimize outcomes and ensure the effective application of MI in rehabilitation programs.

### 4.7 Limitations

This meta-analysis has notable limitations that warrant consideration. First, the small number of studies included in some of the meta-analyses significantly limits the certainty of the results. For example, while the meta-analysis of the vividness of MI were the most consistent, they included only four studies each, raising concerns about the stability of these results. Similarly, the meta-analysis of ability to generate MI and MI-execution temporal congruence would have benefited from additional studies to reduce imprecision.

Second, the heterogeneity observed across meta-analyses may arise from variations in participant sampling, including differences in participant origins, sampling methods, and case–control matching techniques to control for covariates. Additional factors, such as variations in measurement tools and the difficulty levels of the tasks assessed, may also contribute to inconsistencies in the results.

Another important consideration for further research is addressing the influence of covariates, such as physical functioning, spatial representation ability or cognitive functioning, which may have influenced the outcomes. This could be managed through specific recruitment methods, like pairing older and younger adults based on these covariates, and statistically adjusting for these differences in the final analysis. This would help clarifying the real relationship between aging and MI capacities.

## 5 Conclusion

The vividness of MI in KI, IV and EV modalities seem to be preserved in older adults. Additionally, older adults tend to require more time for mental chronometry tasks in TUG, linear walk, and upper limb movements. Implicit MI as assessed through HLJ task indicates that older adults exhibit lower accuracy, greater response times, resulting in decreased efficiency. The ability to generate MI in KI and visual modalities yielded imprecise results, and no clear conclusions could be either drawn regarding MI-execution temporal congruence due to imprecision. Further research is needed, which could potentially modify the current results.

## Data Availability

The original contributions presented in the study are included in the article/Supplementary material, further inquiries can be directed to the corresponding author.
