# Supplementary material for: Exploring the impact of aging on motor imagery abilities: a systematic review with meta-analysis
Source: Front Public Health. 2025 Jan 23;12:1405791. doi: 10.3389/fpubh.2024.1405791 (PMC11801019; doi:10.3389/fpubh.2024.1405791)

**Supplementary Material.**

**Index:**

- Summary table of search engines, databases and search equations.
- Meta-analysis
  - Capacity to generate MI – kinesthetic modality – older adults aged 60-70 years
    - Funnel and Doi plots
  - Capacity to generate MI – kinesthetic modality – older adults aged 70-82 years
    - Funnel and Doi plots
  - Capacity to generate MI - visual modalities
    - Funnel and Doi plots
  - Vividness - kinesthetic modality
    - Funnel and Doi plots
  - Vividness – internal visual modality
    - Funnel and Doi plots
  - Vividness - external visual modality
    - Funnel and Doi plots
  - Temporal features of MI (mental chronometry) - Timed Up and Go test
    - Funnel and Doi plots
  - Temporal features of MI (mental chronometry) - Linear walk (5-10 m)
    - Funnel and Doi plots
  - MI-execution temporal congruence (performance overestimation) - Linear walk (5-10 m)
    - Funnel and Doi plots

Summary table of search engines, databases and search equations.

| **Search Engine** | **Databases** | **Searches (nº)** | **Equation** | **Date** | **Registries (n)** |
| --- | --- | --- | --- | --- | --- |
|  |  |  |  |  |  |
| PubMed | MEDLINE | Nº1 | ("Aged"[Mesh] OR "Geriatrics"[Mesh] OR "Geriatric Assessment"[Mesh] OR “old”[Title/Abstract] OR “older”[Title/Abstract] OR “older adult*”[Title/Abstract] OR “elder*”[Title/Abstract] OR “aging”[Title/Abstract] OR “ageing”[Title/Abstract] OR “aged”[Title/Abstract] OR “geriatr*”[Title/Abstract] OR “gerontology”[Title/Abstract]) AND ("Healthy Aging"[Mesh] OR "Healthy Volunteers"[Mesh] OR “healthy”[Title/Abstract] OR "Parkinson Disease"[Mesh] OR "Parkinson Disease, Secondary"[Mesh] OR "parkinson*"[Title/Abstract] OR "Stroke"[Mesh] OR "stroke"[Title/Abstract] OR "Frail Elderly"[Mesh] OR "frail*"[Title/Abstract] OR "Musculoskeletal Diseases"[Mesh] OR "Orthopedic Procedures"[Mesh] OR "Orthopedics"[Mesh] OR "Musculoskeletal Pain"[Mesh] OR “musculoskeletal disease*”[Title/Abstract] OR “musculoskeletal disorder*”[Title/Abstract] OR “orthopedic surgery”[Title/Abstract] OR “joint surgery”[Title/Abstract] OR "musculoskeletal pain"[Title/Abstract] OR “arthralgia”[Title/Abstract] OR “joint pain”[Title/Abstract] OR “myalgia”[Title/Abstract]) AND (“motor imagery”[Title/Abstract] OR “movement imagery”[Title/Abstract] OR “mental imagery”[Title/Abstract] OR “visual imagery”[Title/Abstract] OR “kinesthetic imagery”[Title/Abstract] OR “kinaesthetic imagery”[Title/Abstract] OR “visuospatial imagery”[Title/Abstract] OR “guided imagery”[Title/Abstract] OR “skill imagery”[Title/Abstract] OR “anticipatory planning”[Title/Abstract] OR “anticipatory motor planning”[Title/Abstract] OR “anticipatory action planning”[Title/Abstract] OR “movement planning”[Title/Abstract] OR “action planning”[Title/Abstract] OR “task planning”[Title/Abstract] OR “mental practice”[Title/Abstract] OR “mental rehearsal”[Title/Abstract] OR “mental training”[Title/Abstract] OR “mental task”[Title/Abstract] OR “motor imagination”[Title/Abstract] OR “movement imagination”[Title/Abstract] OR “imagined movement”[Title/Abstract] OR “action imagination”[Title/Abstract] OR “imagined action”[Title/Abstract] OR “imagined task”[Title/Abstract] OR “mental chronometry”[Title/Abstract] OR “imagery duration”[Title/Abstract] OR “imagery time”[Title/Abstract] OR “laterality judgement”[Title/Abstract] OR “laterality task”[Title/Abstract] OR “left right judgement”[Title/Abstract] OR “body recognition”[Title/Abstract]) | 4^th^ Jan, 2023 | 660 |
|  |  | Nº2 | ("Alzheimer Disease"[Mesh] OR "Alzheimer*"[Title/Abstract]) AND ("motor imagery"[Title/Abstract] OR "movement imagery"[Title/Abstract] OR "mental imagery"[Title/Abstract] OR "visual imagery"[Title/Abstract] OR "kinesthetic imagery"[Title/Abstract] OR "kinaesthetic imagery"[Title/Abstract] OR "visuospatial imagery"[Title/Abstract] OR "guided imagery"[Title/Abstract] OR "skill imagery"[Title/Abstract] OR "anticipatory planning"[Title/Abstract] OR "anticipatory motor planning"[Title/Abstract] OR "anticipatory action planning"[Title/Abstract] OR "movement planning"[Title/Abstract] OR "action planning"[Title/Abstract] OR "task planning"[Title/Abstract] OR "mental practice"[Title/Abstract] OR "mental rehearsal"[Title/Abstract] OR "mental training"[Title/Abstract] OR "mental task"[Title/Abstract] OR "motor imagination"[Title/Abstract] OR "movement imagination"[Title/Abstract] OR "imagined movement"[Title/Abstract] OR "action imagination"[Title/Abstract] OR "imagined action"[Title/Abstract] OR "imagined task"[Title/Abstract] OR "mental chronometry"[Title/Abstract] OR "imagery duration"[Title/Abstract] OR "imagery time"[Title/Abstract] OR "laterality judgement"[Title/Abstract] OR "laterality task"[Title/Abstract] OR "left right judgement"[Title/Abstract] OR "body recognition"[Title/Abstract]) | 4^th^ Jan, 2023 | 63 |
| EBSCO | APA PsycInfo; APA PsycArticles; APA PsycBooks; PSICODOC; APA PsycTherapy; Psychology and Behavioral Sciences Collection; SPORTDiscus with Full Text; CINAHL Complete; Teacher Reference Center; Humanities International Complete; Library, Information Science & Technology Abstracts; Political Science Complete; GreenFILE; Historical Abstracts with Full Text; European Views of the Americas: 1493 to 1750; eBook Collection (EBSCOhost); Anthropology Plus; OpenDissertations; EBSCO eClassics Collection (EBSCOhost); MEDLINE; Academic Search Premier; Education Source; ERIC; Audiobook Collection (EBSCOhost); eBook Open Access (OA) Collection (EBSCOhost); eBook Academic Collection (EBSCOhost); | Nº1 | ((AB “older adult*” OR AB “elder*”) AND (AB “healthy” OR AB “parkinson” OR AB “parkinson disease” OR AB “stroke” OR AB “frail*” OR AB “musculoskeletal disease” OR AB “orthopedic procedure” OR AB “orthopedics” OR AB “musculoskeletal pain” OR AB “musculoskeletal disorder” OR AB “orthopedic surgery” OR AB “joint surgery” OR AB “arthralgia” OR AB “joint pain” OR AB “myalgia”) AND (AB “motor imagery” OR AB “movement imagery” OR AB “mental imagery” OR AB “visual imagery” OR AB “kinesthetic imagery” OR AB “kinaesthetic imagery” OR AB “visuospatial imagery” OR AB “guided imagery” OR AB “skill imagery” OR AB “anticipatory planning” OR AB “anticipatory motor planning” OR AB “anticipatory action planning” OR AB “movement planning” OR AB “action planning” OR AB “task planning” OR AB “mental practice” OR AB “mental rehearsal” OR AB “mental training” OR AB “mental task” OR AB “motor imagination” OR AB “movement imagination” OR AB “imagined movement” OR AB “action imagination” OR AB “imagined action” OR AB “imagined task” OR AB “mental chronometry” OR AB “imagery duration” OR AB “imagery time” OR AB “laterality judgement” OR AB “laterality task” OR AB “body recognition”)) OR ((TI “older adult*” OR TI “elder*”) AND (TI “healthy” OR TI “parkinson” OR TI “parkinson disease” OR TI “stroke” OR TI “frail*” OR TI “musculoskeletal disease” OR TI “orthopedic procedure” OR TI “orthopedics” OR TI “musculoskeletal pain” OR TI “musculoskeletal disorder” OR TI “orthopedic surgery” OR TI “joint surgery” OR TI “arthralgia” OR TI “joint pain” OR TI “myalgia”) AND (TI “motor imagery” OR TI “movement imagery” OR TI “mental imagery” OR TI “visual imagery” OR TI “kinesthetic imagery” OR TI “kinaesthetic imagery” OR TI “visuospatial imagery” OR TI “guided imagery” OR TI “skill imagery” OR TI “anticipatory planning” OR TI “anticipatory motor planning” OR TI “anticipatory action planning” OR TI “movement planning” OR TI “action planning” OR TI “task planning” OR TI “mental practice” OR TI “mental rehearsal” OR TI “mental training” OR TI “mental task” OR TI “motor imagination” OR TI “movement imagination” OR TI “imagined movement” OR TI “action imagination” OR TI “imagined action” OR TI “imagined task” OR TI “mental chronometry” OR TI “imagery duration” OR TI “imagery time” OR TI “laterality judgement” OR TI “laterality task” OR TI “body recognition”)) | 4^th^ Jan, 2023 | 138 |
|  |  | Nº2 | ((AB “Alzheimer” OR AB “alzheimer disease”) AND (AB “motor imagery” OR AB “movement imagery” OR AB “mental imagery” OR AB “visual imagery” OR AB “kinesthetic imagery” OR AB “kinaesthetic imagery” OR AB “visuospatial imagery” OR AB “guided imagery” OR AB “skill imagery” OR AB “anticipatory planning” OR AB “anticipatory motor planning” OR AB “anticipatory action planning” OR AB “movement planning” OR AB “action planning” OR AB “task planning” OR AB “mental practice” OR AB “mental rehearsal” OR AB “mental training” OR AB “mental task” OR AB “motor imagination” OR AB “movement imagination” OR AB “imagined movement” OR AB “action imagination” OR AB “imagined action” OR AB “imagined task” OR AB “mental chronometry” OR AB “imagery duration” OR AB “imagery time” OR AB “laterality judgement” OR AB “laterality task” OR AB “body recognition”)) OR ((TI “Alzheimer” OR TI “alzheimer disease”) AND (TI “motor imagery” OR TI “movement imagery” OR TI “mental imagery” OR TI “visual imagery” OR TI “kinesthetic imagery” OR TI “kinaesthetic imagery” OR TI “visuospatial imagery” OR TI “guided imagery” OR TI “skill imagery” OR TI “anticipatory planning” OR TI “anticipatory motor planning” OR TI “anticipatory action planning” OR TI “movement planning” OR TI “action planning” OR TI “task planning” OR TI “mental practice” OR TI “mental rehearsal” OR TI “mental training” OR TI “mental task” OR TI “motor imagination” OR TI “movement imagination” OR TI “imagined movement” OR TI “action imagination” OR TI “imagined action” OR TI “imagined task” OR TI “mental chronometry” OR TI “imagery duration” OR TI “imagery time” OR TI “laterality judgement” OR TI “laterality task” OR TI “body recognition”)) | 4^th^ Jan, 2023 | 20 |
| Web of Science | Web of Science Core Collection; Current Contents Connect; Derwent Innovations Index; KCI-Korean Journal Database; MEDLINE; SciELO Citation Index. | Nº1 | (TS=("Aged") OR TS=("Geriatric*") OR TS=("Geriatric Assessment") OR AB=(“old”) OR AB=(“older”) OR AB=(“older adult*”) OR AB=(“elder*”) OR AB=(“aging”) OR AB=(“ageing”) OR AB=(“aged”) OR AB=(“geriatr*”) OR AB=(“gerontology”)) AND (TS=("Healthy Aging") OR TS=("Healthy Volunteers") OR AB=(“healthy”) OR TS=(“Parkinson*”) OR AB=(“parkinson*”) OR TS=(“Stroke”) OR AB=(“stroke”) OR TS=(“Frail*”) OR AB=(“frail*”) OR TS=(“Musculoskeletal diseases”) OR TS=(“Orthopedic Procedures”) OR TS=(“Orthopedics”) OR TS=(“Musculoskeletal Pain”) OR AB=(“musculoskeletal disease*”) OR AB=(“musculoskeletal disorder*”) OR AB=(“orthopedic surgery”) OR AB=(“joint surgery”) OR AB=(“musculoskeletal pain*”) OR AB=(“arthralgia”) OR AB=(“joint pain”) OR AB=(“myalgia”)) AND (AB=(“motor imagery”) OR AB=(“movement imagery”) OR AB=(“mental imagery”) OR AB=(“visual imagery”) OR AB=(“kinesthetic imagery”) OR AB=(“kinaesthetic imagery”) OR AB=(“visuospatial imagery”) OR AB=(“guided imagery”) OR AB=(“skill imagery”) OR AB=(“anticipatory planning”) OR AB=(“anticipatory motor planning”) OR AB=(“anticipatory action planning”) OR AB=(“movement planning”) OR AB=(“action planning”) OR AB=(“task planning”) OR AB=(“mental practice”) OR AB=(“mental rehearsal”) OR AB=(“mental training”) OR AB=(“mental task”) OR AB=(“motor imagination”) OR AB=(“movement imagination”) OR AB=(“imagined movement”) OR AB=(“action imagination”) OR AB=(“imagined action”) OR AB=(“imagined task”) OR AB=(“mental chronometry”) OR AB=(“imagery duration”) OR AB=(“imagery time”) OR AB=(“laterality judgement”) OR AB=(“laterality task”) OR AB=(“left right judgement”) OR AB=(“body recognition”)) | 4^th^ Jan, 2023 | 978 |
|  |  | Nº2 | (TS=("Alzheimer Disease") OR AB=(“Alzheimer”)) AND (AB=(“motor imagery”) OR AB=(“movement imagery”) OR AB=(“mental imagery”) OR AB=(“visual imagery”) OR AB=(“kinesthetic imagery”) OR AB=(“kinaesthetic imagery”) OR AB=(“visuospatial imagery”) OR AB=(“guided imagery”) OR AB=(“skill imagery”) OR AB=(“anticipatory planning”) OR AB=(“anticipatory motor planning”) OR AB=(“anticipatory action planning”) OR AB=(“movement planning”) OR AB=(“action planning”) OR AB=(“task planning”) OR AB=(“mental practice”) OR AB=(“mental rehearsal”) OR AB=(“mental training”) OR AB=(“mental task”) OR AB=(“motor imagination”) OR AB=(“movement imagination”) OR AB=(“imagined movement”) OR AB=(“action imagination”) OR AB=(“imagined action”) OR AB=(“imagined task”) OR AB=(“mental chronometry”) OR AB=(“imagery duration”) OR AB=(“imagery time”) OR AB=(“laterality judgement”) OR AB=(“laterality task”) OR AB=(“left right judgement”) OR AB=(“body recognition”)) | 4^th^ Jan, 2023 | 67 |
| ScienceDirect | ScienceDirect | Nº1 | ("older adult" OR "elder") AND (“motor imagery” OR “anticipatory motor planning” OR “imagined movement” OR “mental chronometry” OR “laterality judgement” OR “laterality task” OR “body recognition”)  *Filter – Article Type: Research Articles.* | 4^th^ Jan, 2023 | 240 |
|  |  | Nº2 | "alzheimer" AND (“motor imagery” OR “anticipatory motor planning” OR “imagined movement” OR “mental chronometry” OR “laterality judgement” OR “laterality task” OR “body recognition”)  *Filter – Article Type: Research Articles.* | 4^th^ Jan, 2023 | 183 |
| Scopus | Scopus | Nº1 | (TITLE-ABS-KEY ( "older adult*" ) OR TITLE-ABS-KEY ( "elder*" ) ) AND ( TITLE-ABS-KEY ( "motor imagery" ) OR TITLE-ABS-KEY ( "anticipatory motor planning" ) OR TITLE-ABS-KEY ( "imagined movement" ) OR TITLE-ABS-KEY ( "mental chronometry" ) OR TITLE-ABS-KEY ( "laterality judgement" ) OR TITLE-ABS-KEY ( "laterality task" ) OR TITLE-ABS-KEY ( "body recognition" ) ) | 18^th^ Jan, 2023 | 184 |
|  |  | Nº2 | TITLE-ABS-KEY ( "alzheimer" ) AND ( TITLE-ABS-KEY ( "motor imagery" ) OR TITLE-ABS-KEY ( "anticipatory motor planning" ) OR TITLE-ABS-KEY ( "imagined movement" ) OR TITLE-ABS-KEY ( "mental chronometry" ) OR TITLE-ABS-KEY ( "laterality judgement" ) OR TITLE-ABS-KEY ( "laterality task" ) OR TITLE-ABS-KEY ( "body recognition" ) ) | 18^th^ Jan, 2023 | 14 |
| SciELO | SciELO | Nº1 | ((older adult*) OR (elder*)) AND ((healthy) OR (parkinson) OR (parkinson disease) OR (stroke) OR (frail*) OR (musculoskeletal disease) OR (orthopedic procedure) OR (orthopedics) OR (musculoskeletal pain) OR (musculoskeletal disorder) OR (orthopedic surgery) OR (joint surgery) OR (arthralgia) OR (joint pain) OR (myalgia)) AND ((motor imagery) OR (movement imagery) OR (mental imagery) OR (visual imagery) OR (kinesthetic imagery) OR (kinaesthetic imagery) OR (visuospatial imagery) OR (guided imagery) OR (skill imagery) OR (anticipatory planning) OR (anticipatory motor planning) OR (anticipatory action planning) OR (movement planning) OR (action planning) OR (task planning) OR (mental practice) OR (mental rehearsal) OR (mental training) OR (mental task) OR (motor imagination) OR (movement imagination) OR (imagined movement) OR (action imagination) OR (imagined action) OR (imagined task) OR (mental chronometry) OR (imagery duration) OR (imagery time) OR (laterality judgement) OR (laterality task) OR (left right judgement) OR (body recognition)) | 4^th^ Jan, 2023 | 31 |
|  |  | Nº2 | ((Alzheimer disease) OR (Alzheimer)) AND ((motor imagery) OR (movement imagery) OR (mental imagery) OR (visual imagery) OR (kinesthetic imagery) OR (kinaesthetic imagery) OR (visuospatial imagery) OR (guided imagery) OR (skill imagery) OR (anticipatory planning) OR (anticipatory motor planning) OR (anticipatory action planning) OR (movement planning) OR (action planning) OR (task planning) OR (mental practice) OR (mental rehearsal) OR (mental training) OR (mental task) OR (motor imagination) OR (movement imagination) OR (imagined movement) OR (action imagination) OR (imagined action) OR (imagined task) OR (mental chronometry) OR (imagery duration) OR (imagery time) OR (laterality judgement) OR (laterality task) OR (left right judgement) OR (body recognition)) | 4^th^ Jan, 2023 | 21 |
| Google Scholar | - | Nº1 | (“older adult*” OR “elder*”) AND (“motor imagery” OR “anticipatory motor planning” OR “imagined movement” OR “mental chronometry” OR “laterality judgement” OR “laterality task” OR “body recognition”)  *Filter: Not including citations.*  *Number of registries extracted: The first 100.* | 4^th^ Jan, 2023 | 100 |
|  |  | Nº2 | (“Adulto mayor” OR “adultos mayores” OR “anciano*”) AND (“imaginería motora” OR “planificación motora anticipatoria” OR “movimiento imaginado” OR “cronometría mental” OR “juicio de lateralidad” OR “prueba de lateralidad” OR “reconocimiento corporal”)  *Filter: Not including citations.*  *Number of registries extracted: The first 100.* | 4^th^ Jan, 2023 | 100 |
|  |  | Nº3 | “Alzheimer” AND (“motor imagery” OR “anticipatory motor planning” OR “imagined movement” OR “mental chronometry” OR “laterality judgement” OR “laterality task” OR “body recognition”)  *Filter: Not including citations.*  *Number of registries extracted: The first 100.* | 4^th^ Jan, 2023 | 100 |
|  |  | Nº4 | “alzheimer” AND (“imaginería motora” OR “planificación motora anticipatoria” OR “movimiento imaginado” OR “cronometría mental” OR “juicio de lateralidad” OR “prueba de lateralidad” OR “reconocimiento corporal”)  *Filter: Not including citations.*  *Number of registries extracted: The first 100.* | 4^th^ Jan, 2023 | 100 |

**Capacity to generate MI – kinesthetic modality – older adults aged 60-70 years. Funnel and Doi plots.**


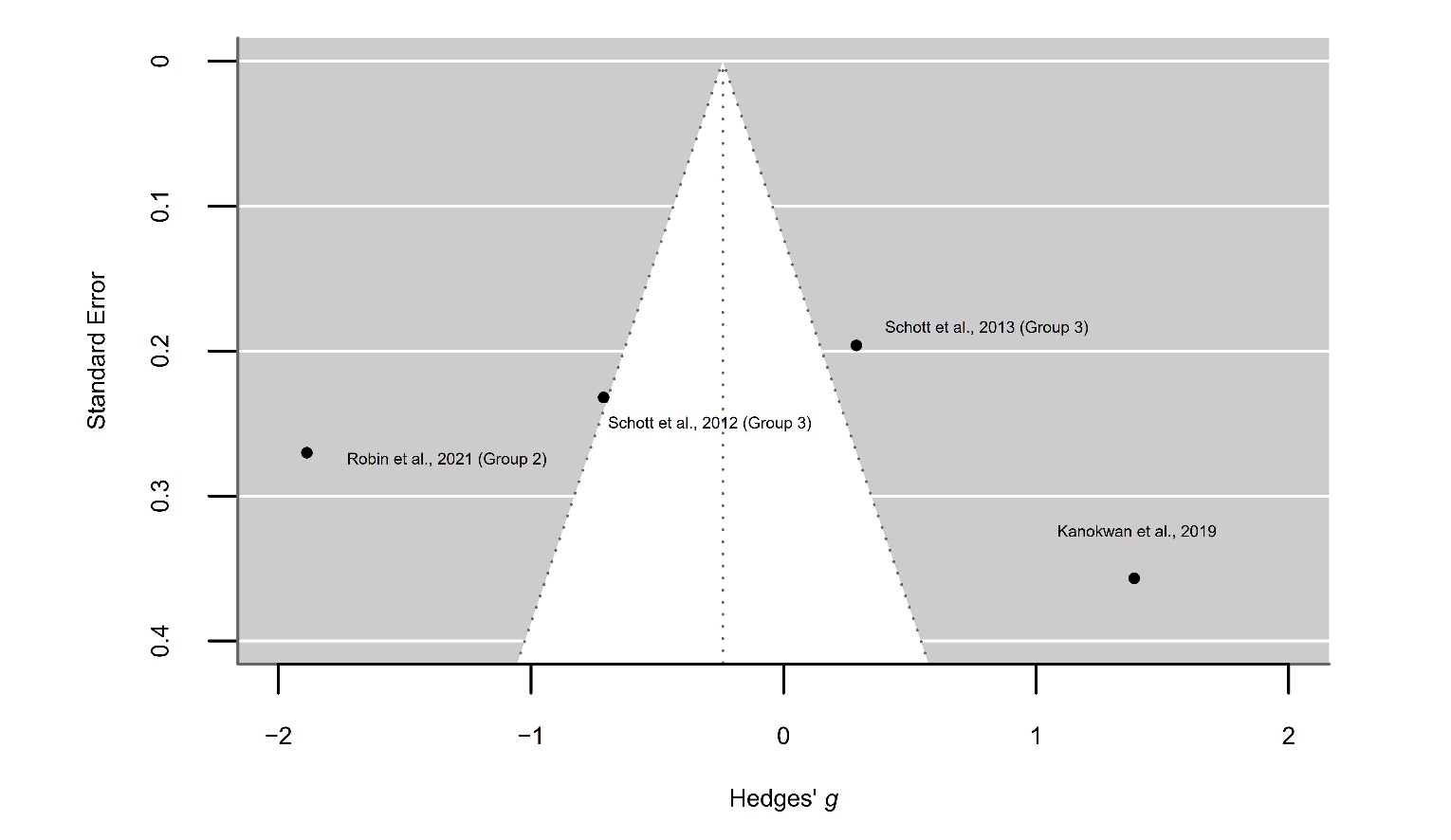

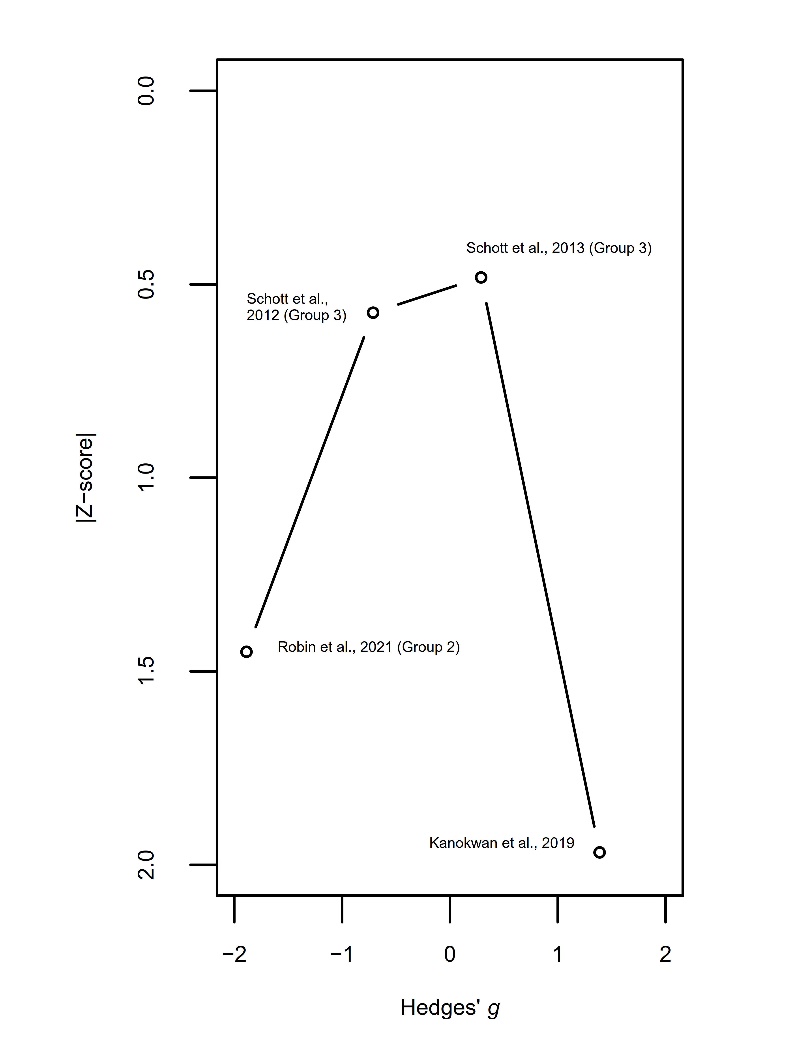


**Capacity to generate MI – kinesthetic modality – older adults aged 70-82 years. Funnel and Doi plots.**


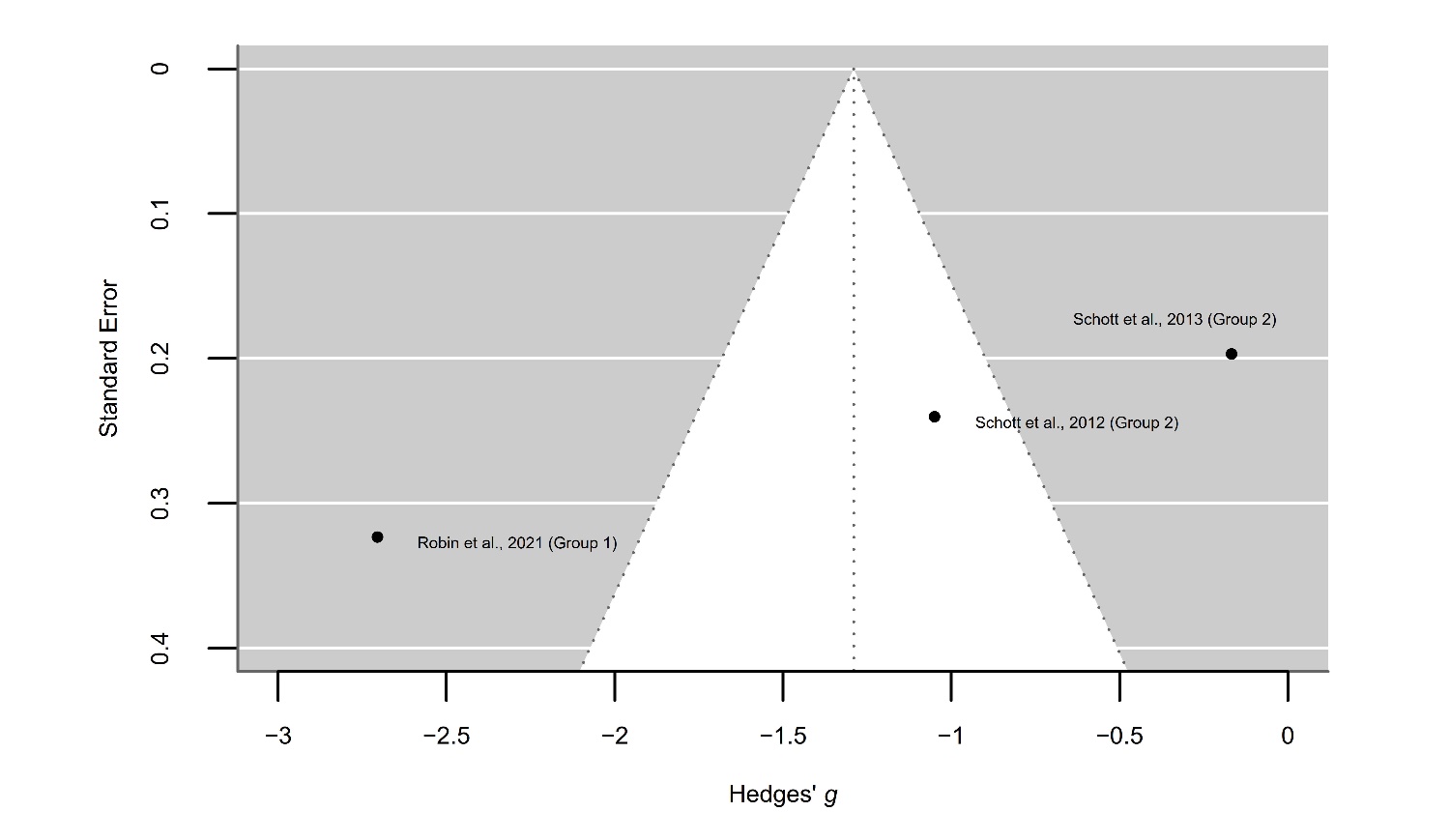

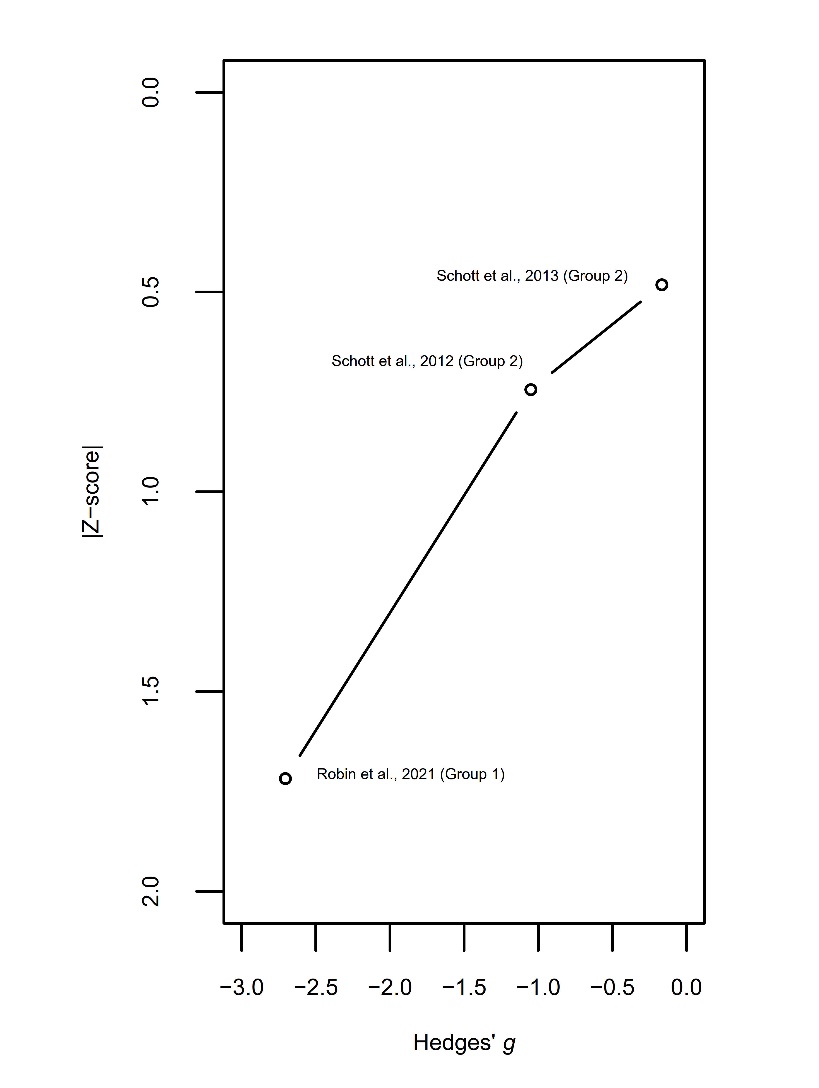


**Capacity to generate MI - visual modalities. Funnel and Doi plots.**


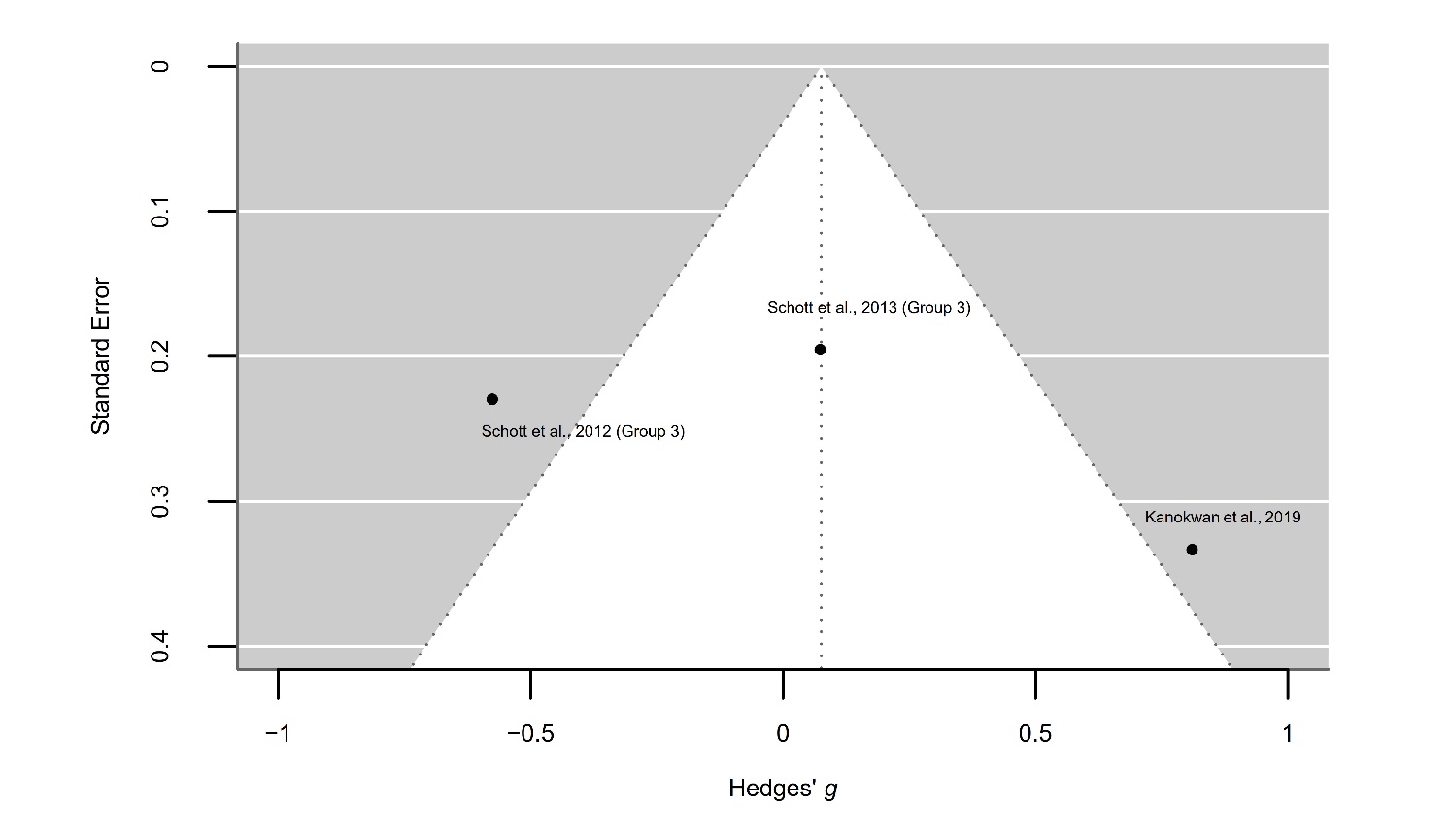

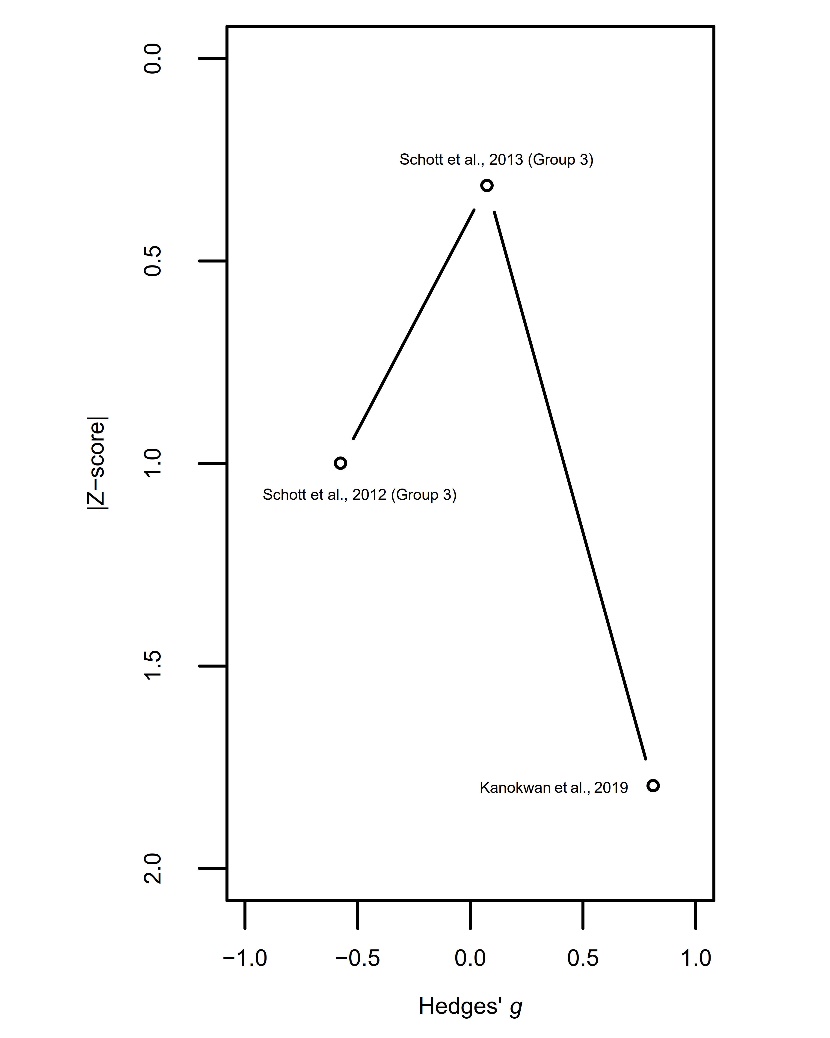


**Vividness - kinesthetic modality. Funnel and Doi plots.**


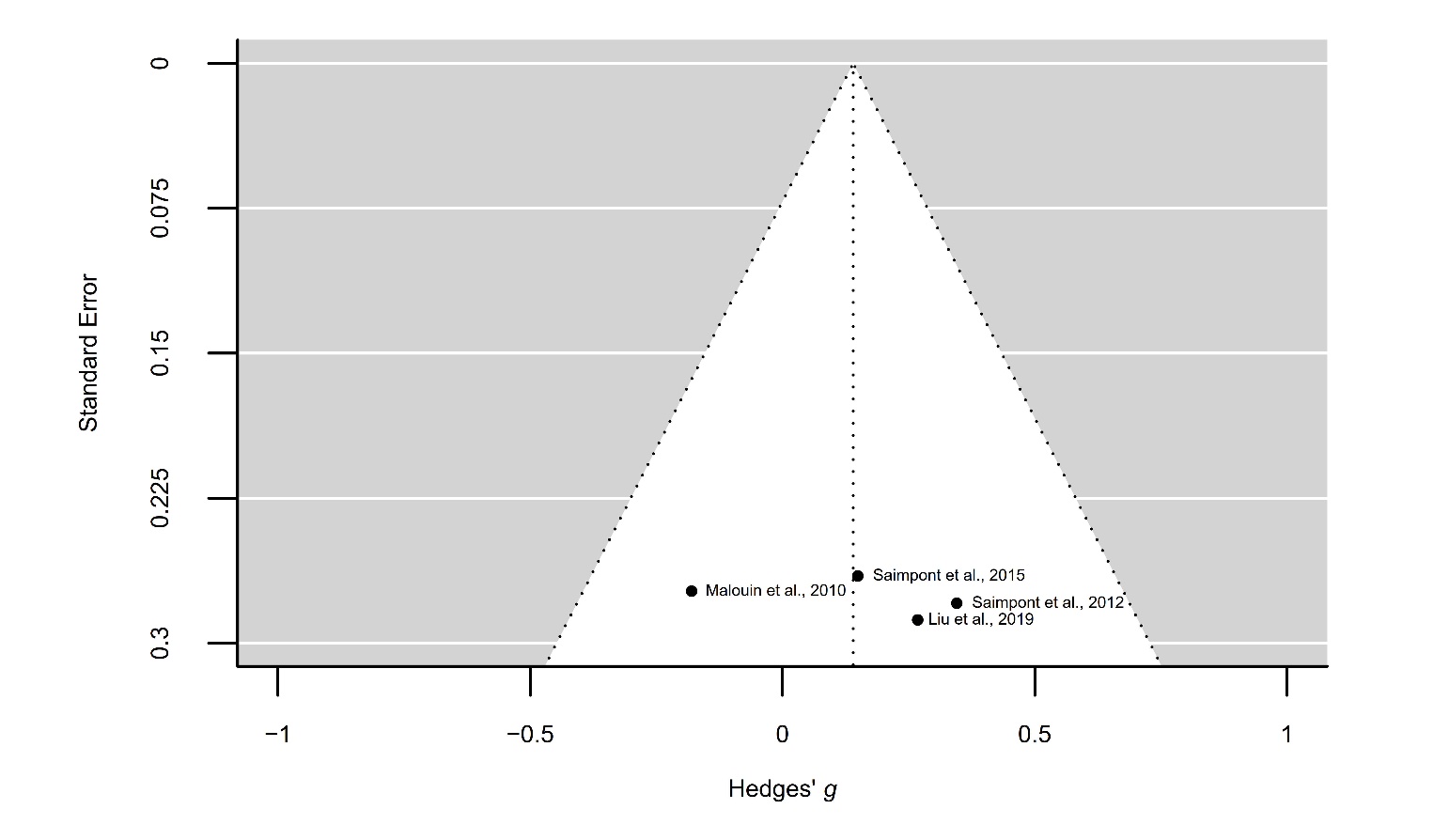

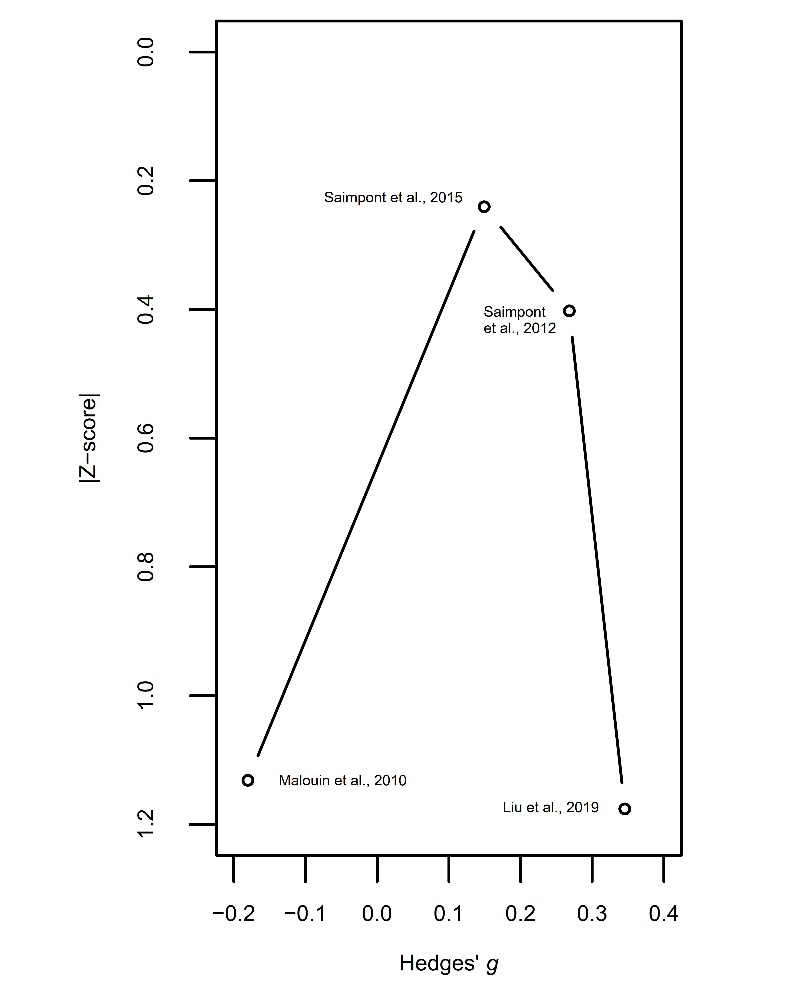


**Vividness – internal visual modality. Funnel and Doi plots.**


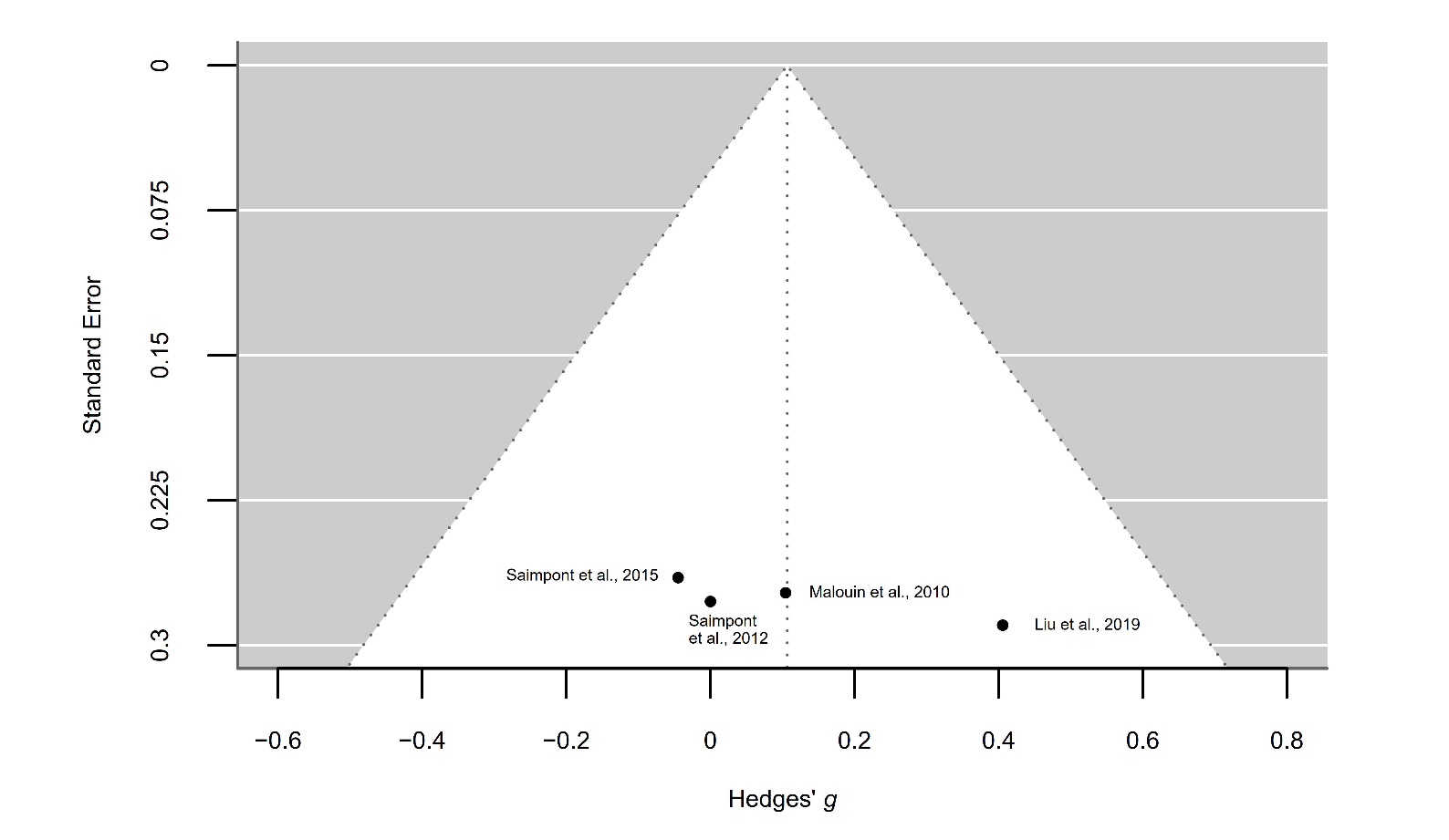

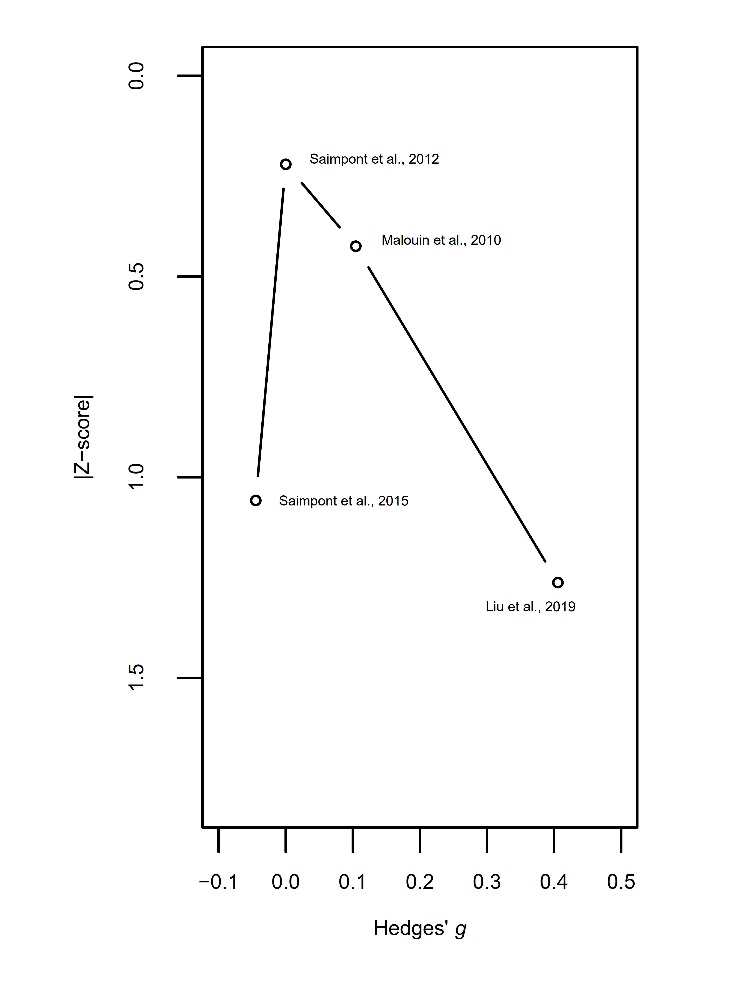


**Vividness - external visual modality. Funnel and Doi plots.**


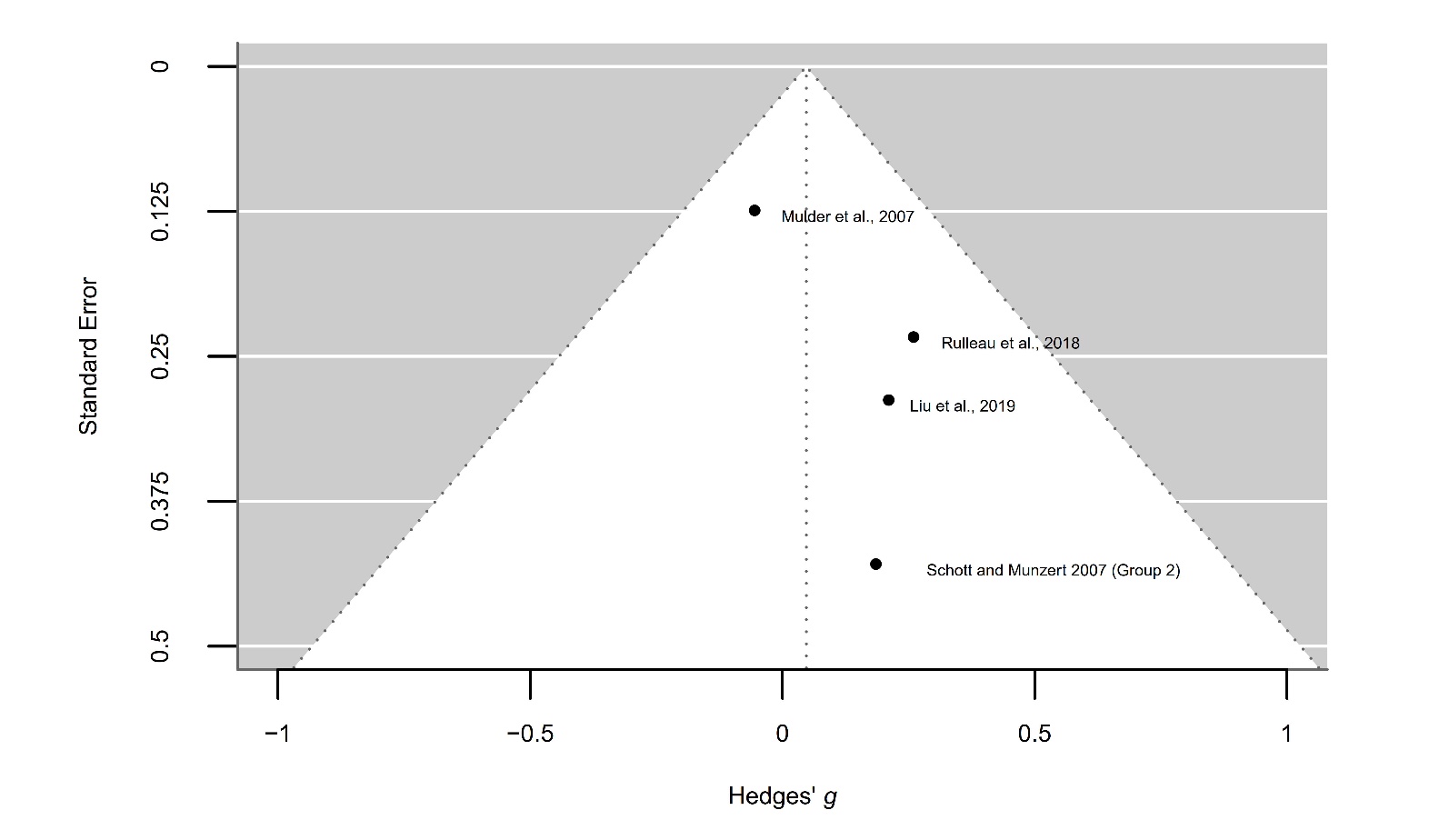

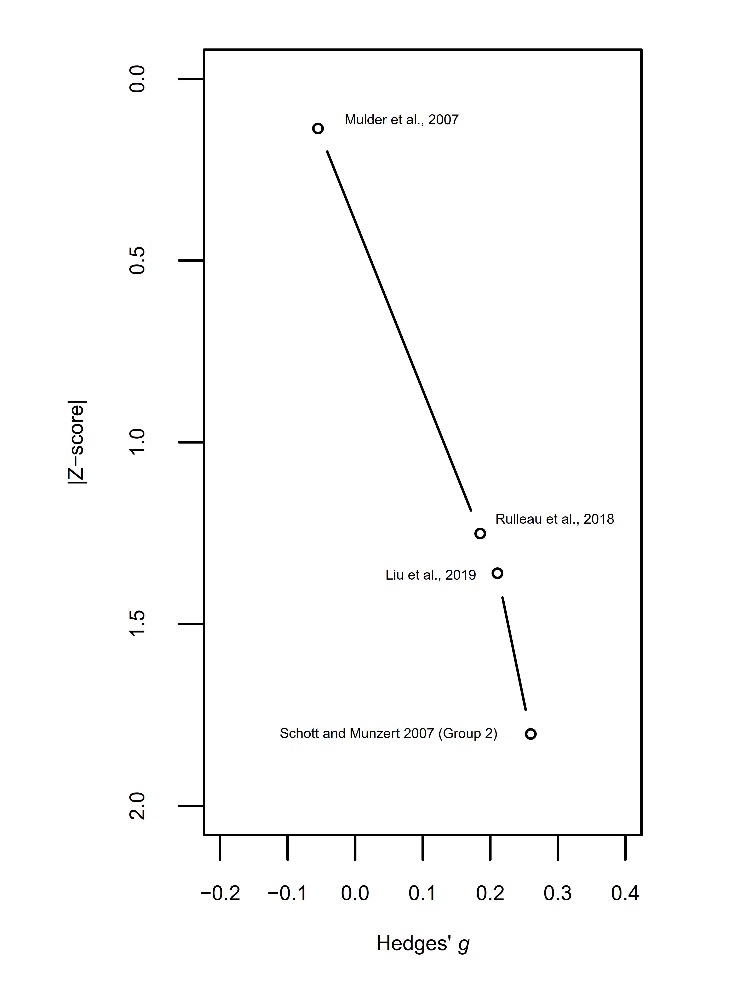


**Temporal features of MI (mental chronometry) - Timed Up and Go test. Funnel and Doi plots.**


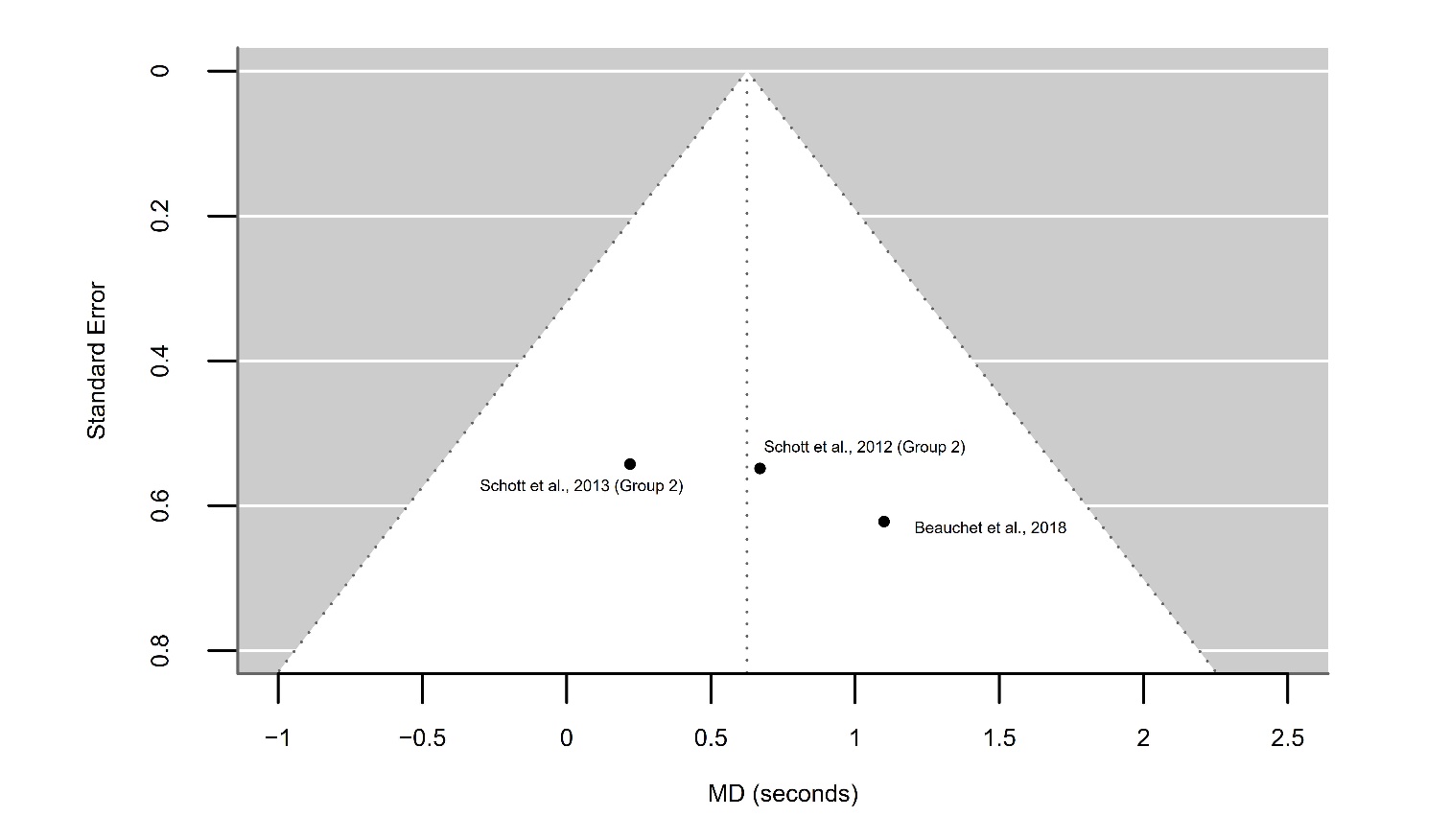

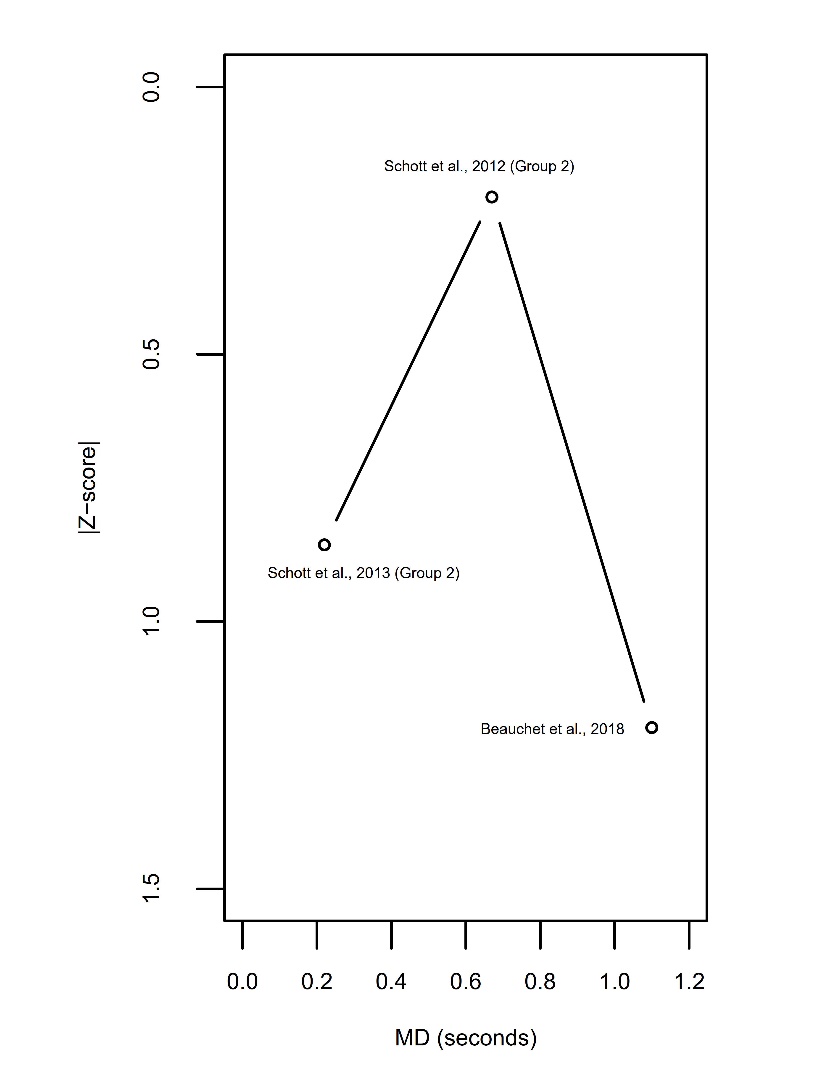


**Temporal features of MI (mental chronometry) - Linear walk (5-10 m). Funnel and Doi plots.**


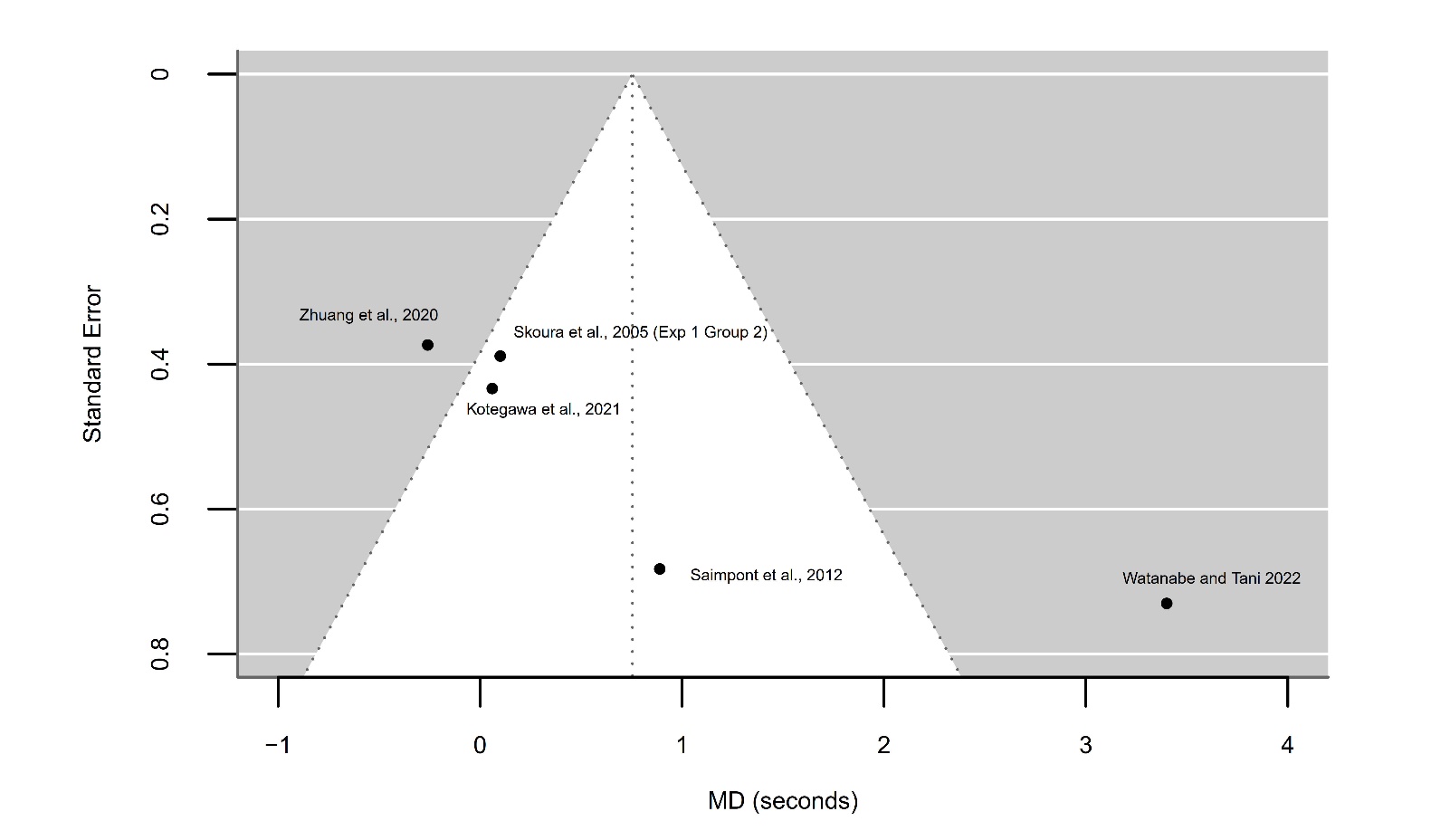

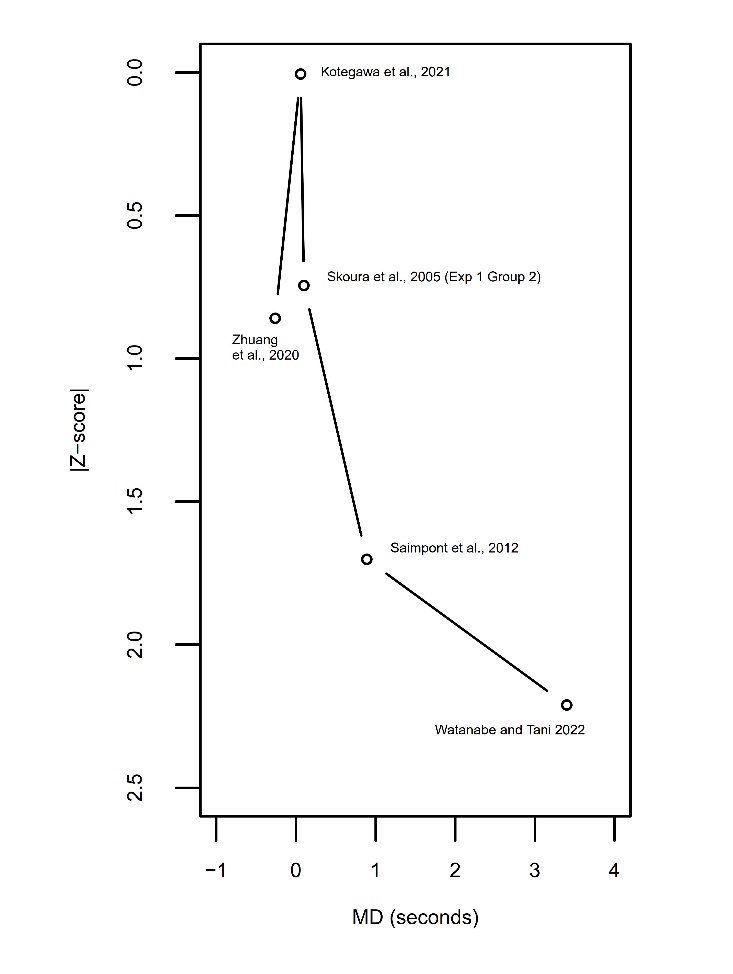


**MI-execution temporal congruence (performance overestimation) - Linear walk (5-10 m). Funnel and Doi plots.**


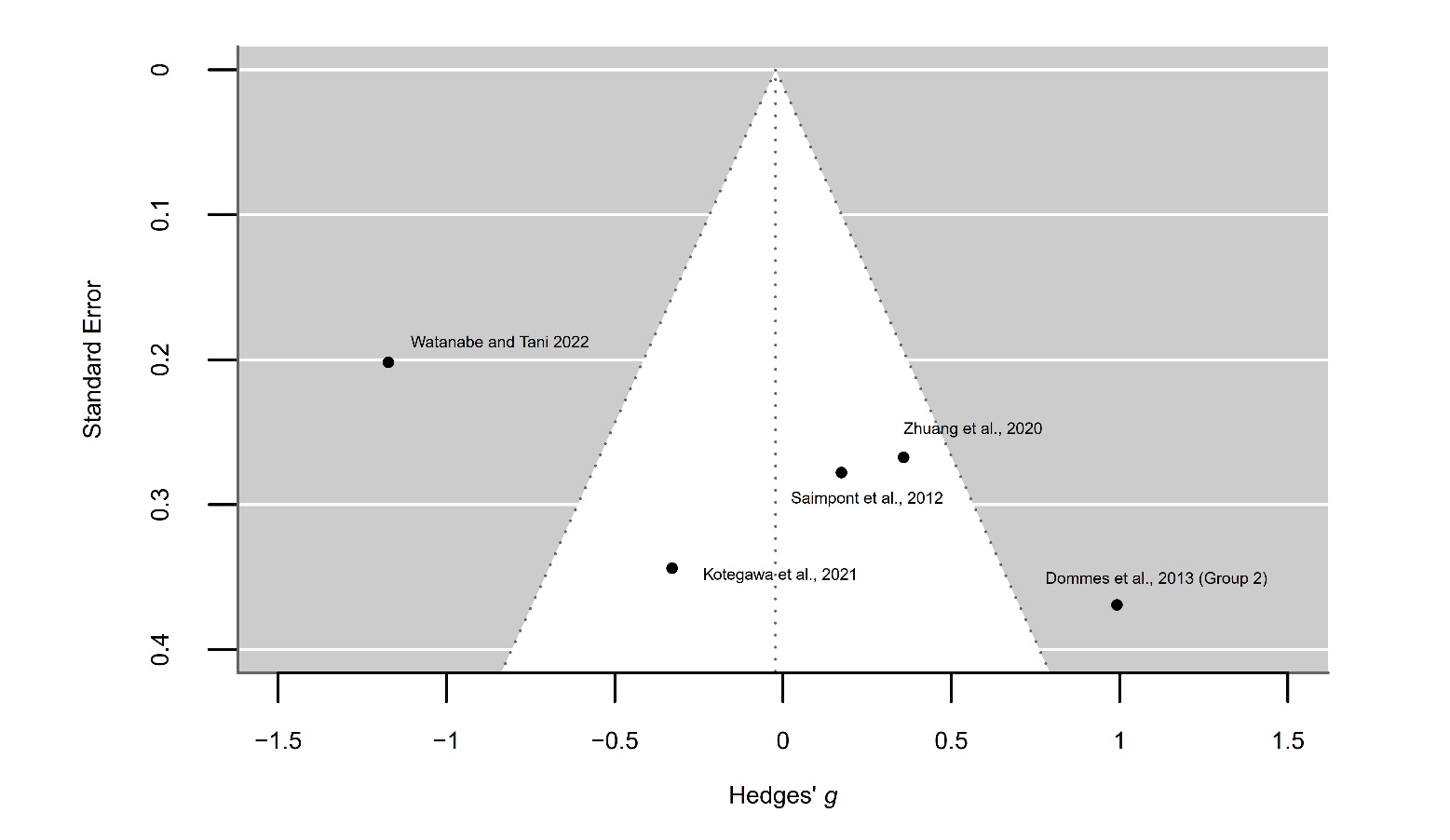

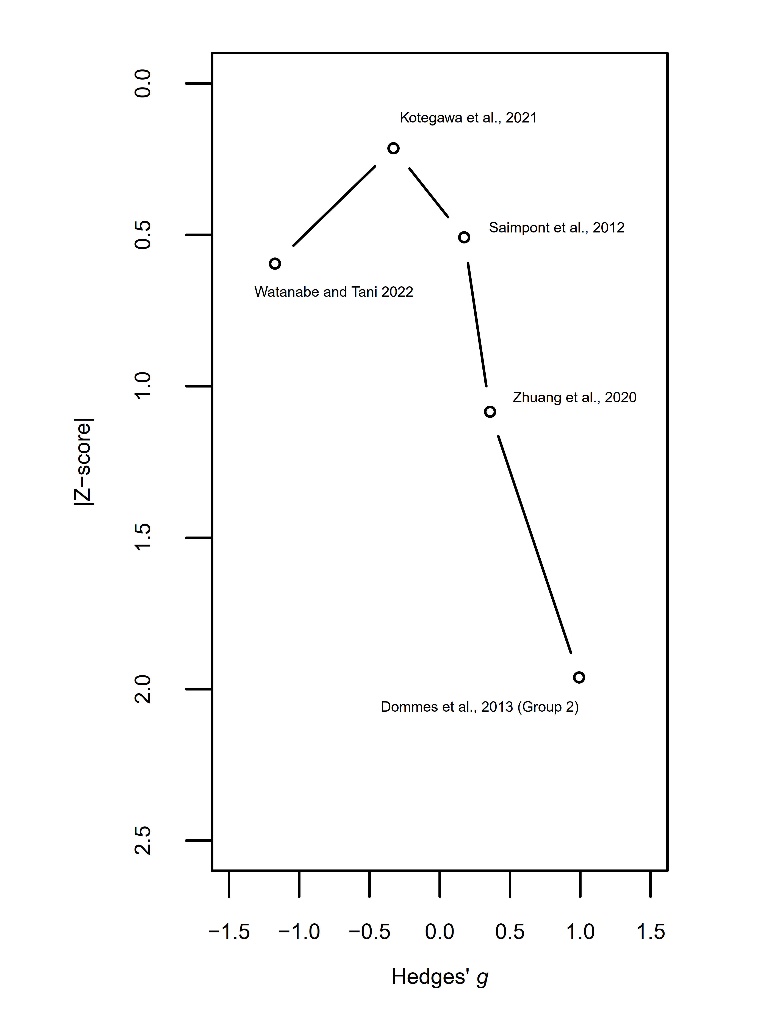

Supplement: Supplementary file 1 [file Supplementary_file_1.docx]
